# Repurposing of Drugs for SARS-CoV-2 Using Inverse Docking Fingerprints

**DOI:** 10.3389/fchem.2021.757826

**Published:** 2021-12-28

**Authors:** Marko Jukič, Katarina Kores, Dušanka Janežič, Urban Bren

**Affiliations:** ^1^ Laboratory of Physical Chemistry and Chemical Thermodynamics, Faculty of Chemistry and Chemical Engineering, University of Maribor, Maribor, Slovenia; ^2^ Faculty of Mathematics, Natural Sciences and Information Technologies, University of Primorska, Koper, Slovenia

**Keywords:** COVID-19, SARS-CoV-2, compound repurposing, protease inhibitors, 3CL^pro^, main protease, inverse docking, fingerprints

## Abstract

Severe acute respiratory syndrome coronavirus 2 or SARS-CoV-2 is a virus that belongs to the *Coronaviridae* family. This group of viruses commonly causes colds but possesses a tremendous pathogenic potential. In humans, an outbreak of SARS caused by the SARS-CoV virus was first reported in 2003, followed by 2012 when the Middle East respiratory syndrome coronavirus (MERS-CoV) led to an outbreak of Middle East respiratory syndrome (MERS). Moreover, COVID-19 represents a serious socioeconomic and global health problem that has already claimed more than four million lives. To date, there are only a handful of therapeutic options to combat this disease, and only a single direct-acting antiviral, the conditionally approved remdesivir. Since there is an urgent need for active drugs against SARS-CoV-2, the strategy of drug repurposing represents one of the fastest ways to achieve this goal. An *in silico* drug repurposing study using two methods was conducted. A structure-based virtual screening of the FDA-approved drug database on SARS-CoV-2 main protease was performed, and the 11 highest-scoring compounds with known 3CL^pro^ activity were identified while the methodology was used to report further 11 potential and completely novel 3CL^pro^ inhibitors. Then, inverse molecular docking was performed on the entire viral protein database as well as on the *Coronaviridae* family protein subset to examine the hit compounds in detail. Instead of target fishing, inverse docking fingerprints were generated for each hit compound as well as for the five most frequently reported and direct-acting repurposed drugs that served as controls. In this way, the target-hitting space was examined and compared and we can support the further biological evaluation of all 11 newly reported hits on SARS-CoV-2 3CL^pro^ as well as recommend further in-depth studies on antihelminthic class member compounds. The authors acknowledge the general usefulness of this approach for a full-fledged inverse docking fingerprint screening in the future.

## Introduction

Severe acute respiratory syndrome coronavirus 2 or SARS-CoV-2 is a virus that belongs to the *Coronaviridae* family and is named after the crown serrations on its surface (Kahn, and McIntosh, 2005; Cui et al., 2019). It is a single-stranded positive sense RNA (+ssRNA) virus (Gorbalenya et al., 2020; Zhu N. et al., 2020). This group of viruses commonly causes colds but has tremendous pathogenic potential. In humans, an outbreak of SARS (severe acute respiratory syndrome) caused by the SARS-CoV virus was first reported in mainland China and Hong Kong in 2003, followed by 2012, when the Middle East respiratory syndrome coronavirus (MERS-CoV) led to an outbreak of Middle East respiratory syndrome (MERS) in Saudi Arabia, mainland China, United Arab Emirates, and the Republic of Korea (Hilgenfeld and Peiris, 2013; DeWit et al., 2016). More recently, swine acute diarrhea syndrome coronavirus (SADS-CoV) causing severe acute porcine diarrhea syndrome has also been described with high porcine pathogenicity on top of a variety of documented coronaviruses in other animals (Yang et al., 2020; Lin et al., 2021).

The emergence of the COVID-19 disease caused by the SARS-CoV-2 pathogen was reported in major media in December 2019 to have originated in Wuhan, Hubei, China (Wu et al., 2020), and spread worldwide in the first months of 2020, causing a pandemic of the COVID-19 disease (Li et al., 2020; Wang, et al., 2020). COVID-19 is a serious socioeconomic and global health problem that has claimed more than 4,294,225 lives at the time of writing this article (Nicola et al., 2020). Indeed, the majority of cases present with only mild symptoms, while a variable percentage (0.2%–5%) of patients progress to pneumonia and multiorgan failure, which can lead to death, especially without medical assistance at the secondary healthcare level (O’Driscoll et al., 2021; Malik et al., 2021). The medical and academic communities, as well as the pharmaceutical industry, have responded immediately with intensive research campaigns aimed primarily at uncovering pathogenicity mechanisms, researching new drugs and developing vaccines, accompanied by new social guidelines and the dissemination of information and good hygiene practices by the relevant authorities (Fry et al., 2020; Meier et al., 2020). Registered SARS-CoV-2 vaccines are available (Chen C. Z. et al., 2020; Amanat and Krammer, 2020), and they represent the forefront in battle against COVID-19, but the high viral mutation rate, which can lead to structural changes in key viral proteins, may render available vaccines ineffective (Naqvi et al., 2020). In late 2020, a novel SARS-CoV-2 alpha variant (B.1.1.7; Volz et al., 2021) and a beta variant (B.1.351; Tegally et al., 2020) were reported, followed by a gamma variant (P.1; Faria et al., 2021) and a new SARS-CoV-2 variant delta/delta+ in 2021 (B.1.617/AY.1), causing new infections and reinfections that are slowly spreading throughout the world (Moelling, 2021; Roy, et al., 2021). We can respond by developing novel vaccines, but even with novel technologies such as mRNA, the response time is substantial (Badgujar et al., 2020; Verbeke et al., 2021). Therefore, the development of other therapeutic options and novel drug approaches are essential for the future control of coronavirus infections (Kaddoura et al., 2020; Pooladanda et al., 2020; Sarkar et al., 2020).

**FIGURE 1:**
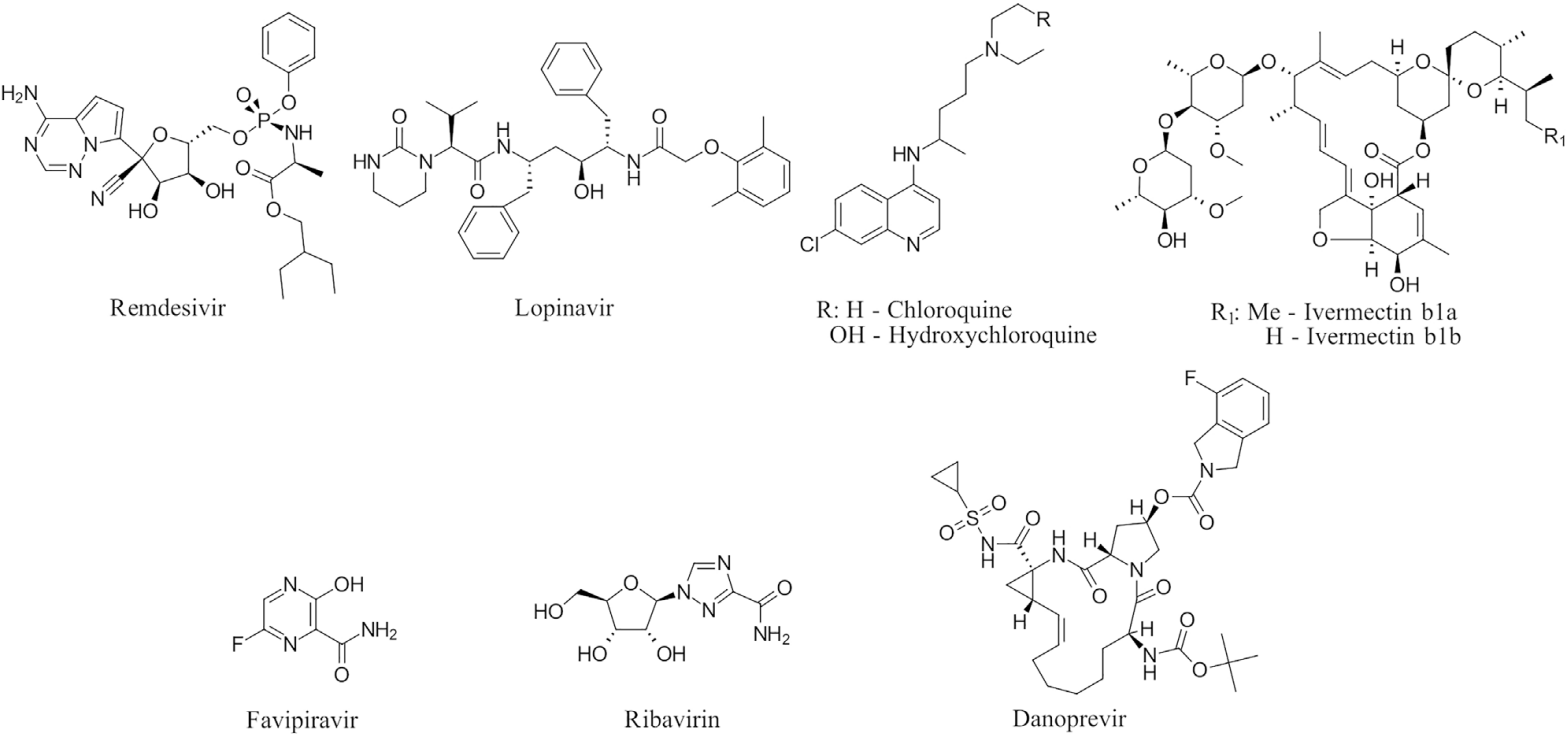
Commonly repurposed drugs against SARS-CoV-2 with proposed direct action (ritonavir is mainly a pharmacokinetic modifier for its partner in combination therapy).

To date, there are only a handful of therapeutic options to combat this disease, with only one direct-acting antiviral, remdesivir, conditionally approved in Taiwan, followed by a rapid succession of conditional approvals in the EU and Canada. Following these conditional approvals, an emergency approval for remdesivir (a prodrug of GS-441524) was granted in the US and Japan in May 2020 (Lamb, 2020). There is a tremendous research effort underway to develop novel drugs (Jin et al., 2020; Günther et al., 2021; Jukič et al., 2020), but given the immediate need for active compounds against SARS-CoV-2, the strategy of drug repurposing represents one of the fastest options toward this goal (Dotolo et al., 2021; Singh et al., 2020; Gatti and De Ponti, 2021). Most notably, the most commonly reported and direct-acting repurposed drugs include the antiviral agents favipiravir (Coomes and Haghbayan, 2020), lopinavir (dynamic)–ritonavir (kinetic) (Ye et al., 2020), ribavirin (Khalili et al., 2020), interferons (Zhou et al., 2020), the anthelmintic ivermectin (Schmith et al., 2020), and the antimalarials chloroquine (Cortegiani et al., 2020) or hydroxychloroquine (Meo et al., 2020), all shown in Figure 1. Multiple reviews on this subject beyond the scope of this article are available to the reader (Saha et al., 2020; Sourimant et al., 2021). Similar to remdesivir, favipiravir is a viral RdRp inhibitor, ribavirin inhibits IMPDH2, and lopinavir together with ivermectin inhibits viral 3CL^pro^. Chloroquine/hydroxychloroquine is thought to modulate viral endosome maturation and interact with sigma receptors (Abate et al., 2020; De et al., 2021). Other targeting approaches such as ACE2−RBD interaction have also been examined for drug repurposing (Hanson et al., 2020; Wei et al., 2020; de Oliveira et al., 2021) and compound sets evaluated on cell lines *in vitro* (Chen W. H. et al., 2020; Riva et al., 2020; Bakowski et al., 2021). Especially on examination of sigma receptor ligands, a key observation was made where phospholipidosis was a shared mechanism underlying the antiviral activity of many repurposed drugs (hydroxychloroquine, azithromycin, amiodarone). Mehanistically, this disrupts lysosomal lipid catabolism and trafficking and results in an *in vitro* correlation between drug-induced phospholipidosis and antiviral activity disrupting the clear mechanism-based design decisions (Tummino et al., 2021). This is especially evident in amphiphilic compounds and depends on the physicochemical properties (cLogP ≥ 3 and pKa ≥ 7.4) of drugs. Therefore, in this work, this was especially considered and compounds flagged in order to focus on molecules with therapeutic potential.

**FIGURE 2:**
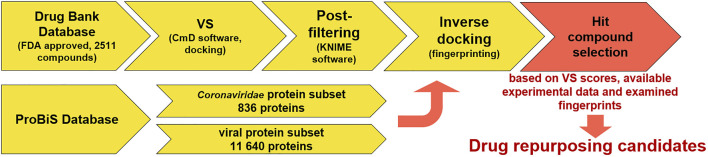
Virtual screening coupled to inverse docking protocol for drug repurposing. The first step is database selection; next is the VS campaign followed by a filtering step (filter off pains) with a final inverse docking fingerprint calculation to examine the target space where the compounds produce favorable binding poses (to this end, the ProBiS database was employed to conduct two experiments in parallel—one with all viral proteins and the other with focused *Coronaviridae* proteins). Hit selection was performed based on VS scores and prior available experimental data (ChEMBL) along with examination of inverse docking fingerprints.

In the context of anti-coronavirus therapeutics—all repurposing approaches with a large body of conflicting data—we sought to conduct a transparent *in silico* drug repurposing study using known FDA-approved drugs on a well-described SARS-CoV-2 target 3CL^pro^ or M^pro^ (Zhu W. et al., 2020). This target is a first choice for repurposing campaigns due to its extensive experimental support (Gordon et al., 2020), available crystallographic data, and good biological evaluation data (Anand et al., 2003; Chiou et al., 2021; Osipiuk et al., 2021). The protease is an attractive target as it plays a central role in the viral life cycle by processing the viral polyproteins pp1a and pp1ab at multiple distinct cleavage sites and complementary reports on repurposing research are available, further contextualizing the work herein (Kuzikov et al., 2021). We proceeded to compute the inverse docking fingerprints to provide a focused outlook on a typical repurposing scenario and propose a consensus on the identified hit compounds. The work reveals a rarely studied *in silico* selectivity, and the authors have not overlooked the usefulness of this approach for future inverse docking fingerprint screening experiments to identify compounds that behave similarly across a large number of targets or explore the interactome (Sadegh, et al., 2020; Figure 2).

The inverse docking procedure has already been used as a tool for drug repurposing before with positive results (Kharkar et al., 2014; Wang et al., 2019). As an example, it revealed the potential new targets for tanshinone IIA used in the treatment of acute promyelocytic leukemia (Chen, 2014). More recently, Ribone et al. (2021) applied inverse docking using multiple scoring functions for target proposal on SARS-CoV-2-repurposed drugs and discovered new potential targets for drugs with experimentally determined activity against SARS-CoV-2.

## Materials and methods

### Database preparation

The compounds were obtained from the Drug Bank Database as a subset of 2,511 drugs in sdf format (Wishart et al., 2018). The database was further expanded to 4,711 entries with calculation of tautomeric structures and ionization at pH 7.4 with further standard structure preparation steps such as enumeration of undefined chiral centers, removal of structural defects, and 3D structure minimization with optimization (using the OPLS3e force field) toward the final 3D conformation. For this work, the LigPrep tool from Schrödinger (Release Schrodinger 2020-4, Schrödinger, LLC, New York, NY, United States, 2020) was used. The exact parameters were as follows: ligprep -bff 16 -i 2 -ph 7.4 -pht 1.0 -s 8 -orig_file -orig_file_index 1 -isd input -osd output (Shelley et al., 2007; Greenwood et al., 2010).

Ivermectin B1a/B1b and selamectin were modeled using the Avogadro chemical editor (Hanwell et al., 2012) and then optimized using Gaussian 16 (Frisch et al., 2016) in conjunction with the B3LYP method and the 6-31G(d) basis set, in the absence of a suitable 3D structure. The 3D structures of all other molecules used were obtained from the Drug Bank Database, ionized using LigPrep Ioniser software (Release Schrodinger 2020-4, Schrödinger, LLC, New York, NY, United States, 2020) at pH 7.4 ± 1 and their geometry optimized using the RDKit geometry optimization node in KNIME software.

### Target preparation

We chose a well-described SARS-CoV-2 3CL^pro^ complex with PDB ID: 6Y7M and a resolution of 1.9 Å (Zhang et al., 2020). The complex contains {tert}-butyl ∼{N}-[1-[(2∼{S})-3-cyclohexyl-1-[[(2∼{S},3∼{R})-4-(cyclopropylamino)-3-oxidanyl-4-oxidanylidene-1-[(3∼{R})-2-oxidanylidene-3,4-dihydropyrrol-3-yl]butan-2-yl]amino]-1-oxidanylidene-propan-2-yl]-2-oxidanylidene-pyridin-3-yl]carbamate (OEW), a peptide-like covalent inhibitor (MW = 585.69 g/mol). As described previously (Jukič et al., 2021), this complex comprises the peptidomimetic inhibitor OEW, which occupies all major pockets at the active site of the enzyme, leaving the S1 pocket accessible and the enzyme in the active conformation. After superposition with a reference structure (PDB ID:6LU7), the catalytic binding pocket was defined around Cys145 (Jin et al., 2020). The covalent OEW bond was cleaved, the small molecule removed, and the Cys145 amino-acid residue regenerated (open-source PyMOL, version 2.1; DeLano, 2002). The target was prepared using the protein preparation module of Schrödinger Small-Molecule Discovery Suite (Release Schrödinger 2020-4, Schrödinger, LLC, New York, NY, United States, 2020). Missing hydrogen atoms were added, the H-bond network was optimized using the PROPKA tool at pH 7.4, waters were removed, and restrained minimization was performed with convergence of heavy atoms toward 0.3 Å. Finally, a docking receptor (Morley and Afshar, 2004) was generated with the docking package CmDock (https://gitlab.com/Jukic/cmdock/) using the program cmcavity. The reference ligand method was used to calculate the cavity (receptor definition), where we used the OEW-cleaved regenerated ligand as a reference and a sphere of 7 Å around the ligand to calculate the docking volume. We calculated a total docking volume of 3,106.25 A3 and included the calculated cavity (Cavity #1) in the definition of the docking receptor. The parameters of cavity #1 were size of 24,850 points, min = (−33.5, −53.5, −8.5), max = (−13, −26.5, 12), center = (−24.4138, −38.9632, −0.179235), and extent = (20.5, 27, 20.5) Å (Figure4A).

### Structure-based virtual screening

In the virtual screening experiment, we performed molecular docking using CmDock (CmD) software (https://gitlab.com/Jukic/cmdock/; Ruiz-Carmona et al., 2014). The inputs were the FDA-approved drug database precalculated by LigPrep and the prepared receptor (cavity #1) as described in the previous section. First, we performed a redocking experiment in which we successfully redocked a regenerated OEW reference ligand (PDB ID: 6Y7M) and obtained the binding conformation of the crystal complex with a root mean square deviation (RMSD) of 1.34 Å. The CmD parameters were the standard docking protocol (dock.prm) with 100 runs, no constraints, and no score filters. Using the same exhaustive docking protocol, we performed the virtual screening experiment with the 4,711 FDA-approved drugs and analyzed the docking results using KNIME software (Fillbrunn et al., 2017). The minimum docking score was −37.2, the maximum was 8.3, and the mean was -11.7 with a standard deviation of 5.8 after exhaustive docking with CmDock at the prepared receptor binding site (cavity #1).

### Inverse docking

We applied an inverse molecular docking approach to 11,640 viral protein structures, including 836 protein structures from the taxonomic lineage of *Coronaviridae*. Small molecule-binding sites were identified and prepared for inverse molecular docking using the ProBiS-Dock system (Depolli et al., 2013; Konc et al., 2021). By reducing the size of the docking space and focusing on the binding sites, the time and complexity of inverse molecular docking are reduced. The creation of the ProBiS docking database, which served as a template for our viral database, is further described in Štular et al. (2016) and Konc et al. (2021) and has already been successfully used for mechanistic insights into the side effects of troglitazone and rosiglitazone (Kores et al., 2021).

The inverse docking CANDOCK algorithm (Fine et al., 2020) applies a hierarchical approach to small-molecule reconstruction from the atomic lattice using generalized statistical potential functions and graph theory. The docking scores represent an approximation to the relative free energies of binding and have arbitrary units. The algorithm works in several consecutive steps. First, a small molecule is taken and broken into fragments. Then, the fragments are docked into protein-binding sites from the database using knowledge-based scoring methods. Then, the best-docked fragments are selected and linked using a fast maximum clique algorithm (Konc et al., 2021). In the course of the reconstruction, iterative dynamics is used for better placement of the ligand in the binding cavity. In a second step, the conformation optimization procedure is performed (Štular et al., 2016; Fine et al., 2020; Kores et al., 2021). Validation of the algorithm has been extensively reported beforehand (Furlan et al., 2018; Kores et al., 2019; Fine et al., 2020; Kores et al., 2021). To sum up the validation procedures, we employed multiple methods. First is the redocking procedure, reported by Kores et al. (2019) and Kores et al. (2021). In this procedure, the known structures from the Protein Data Bank (PDB) with co-crystallized ligands are taken, and the ligand redocking is preformed to show that the used docking algorithm can produce similar poses with the highest docking score to the native (co-crystallized) ones. This was done for resveratrol (Kores et al., 2019) and also for troglitazone and rosiglitazone (Kores et al., 2021), where good agreement between predicted and experimentally determined poses was shown based on the RMSD of atomic positions. The second method combines the calculation of receiver operating characteristics (ROC), enrichment, and predictiveness curves (PC). The usage was reported by Furlan et al. (2018), Fine et al. (2020), and Kores et al. (2021). Here the experimentally confirmed protein targets from the ChEMBL database for ligands were used to determine if the CANDOCK protocol produced similar binding targets. This analysis was done for curcumin (Furlan et al., 2018) and troglitazone and rosiglitazone (Kores et al., 2021), where it showed that the CANDOCK protocol is expected to provide a good agreement with experiments. Top scoring hits identified by our methodology demonstrate also an experimental activity on 3CL^pro^ (CHEMBL4495582, CHEMBL4495583). Therefore, our proposed protocol and the workflow are suitable for identification of novel potential actives on the studied target.

### Inverse docking fingerprinting

**FIGURE 3:**
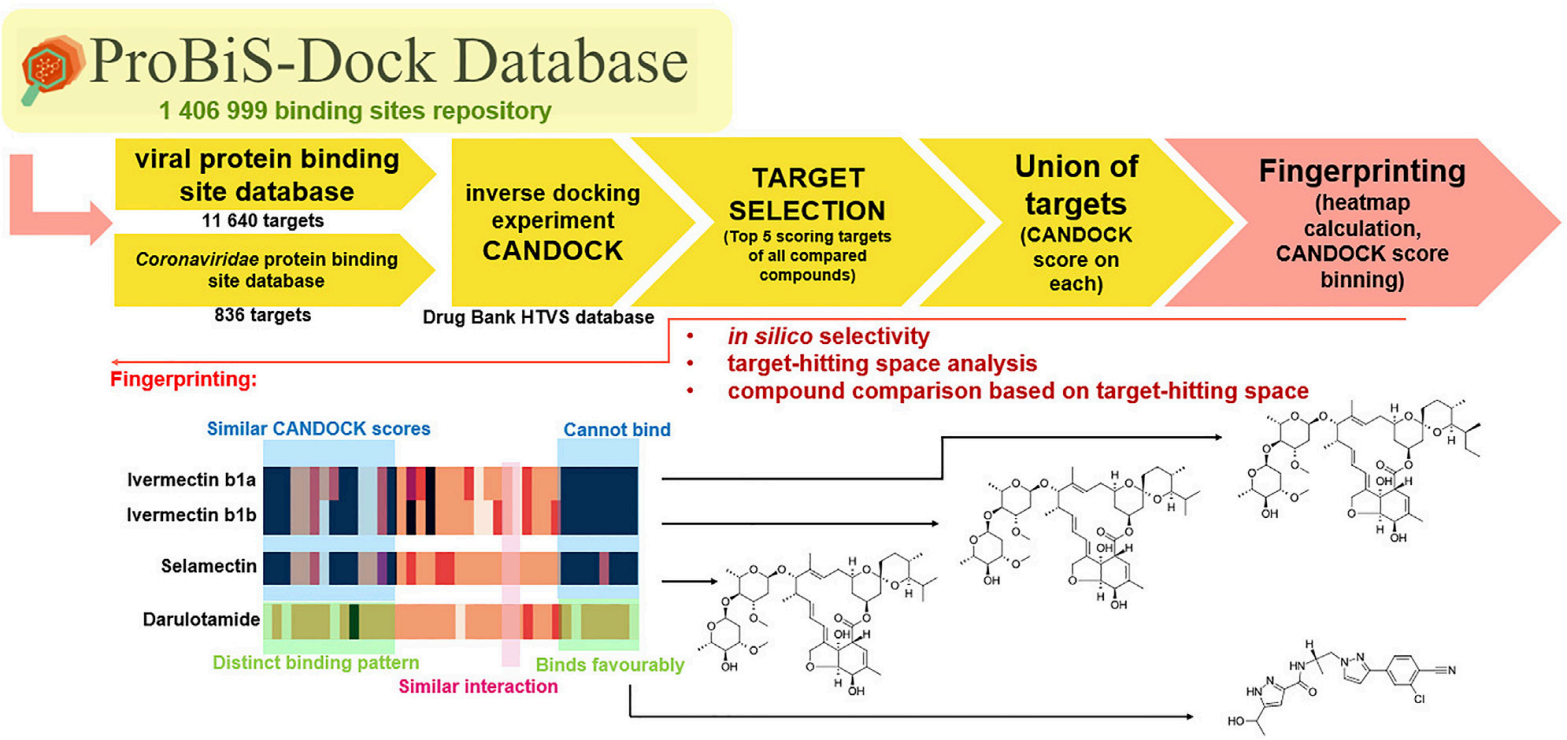
Inverse docking fingerprinting protocol and example of fingerprint comparison. Fingerprinting using heatmaps is useful for determining the similar binding patterns and find targets where compounds bind favorably. The darker the color in the fingerprint heatmaps, the less favorable the binding of the compound into target.

We used five reference compounds, namely, chloroquine and hydroxychloroquine, ivermectin B1a and B1b, and selamectin, which are commonly reused drugs as control compounds (Figure 1). In addition, the 11 compounds that scored highest in the virtual screen (Table 1), all with previously reported activity on 3CL^pro^, and 11 compounds from our virtual screening experiment with the highest score that had not previously been reported to have activity on 3CL^pro^ (CHEMBL4495582, CHEMBL4495583; Table 2) were examined. Using the CANDOCK inverse docking protocol, we elaborated their binding potential against two previously created databases of binding sites (viral protein target database—11,640 targets as well as 836 Coronaviridae protein targets) with a total of more than 325,000 individual docking experiments (Figure 3).

**TABLE 1:** Identified top-scoring drugs in the virtual screening repurposing experiment on the SARS-CoV-2 main protease 3CL^pro^ with previously reported activity on 3CL^pro^ (CHEMBL4495582, CHEMBL4495583).

No.	Structure	Mr (g/mol)	Name (INN)^a^	CmDock docking score^b^	Classification ^c^	Phospholipidosis potential *in silico* ^d^
1	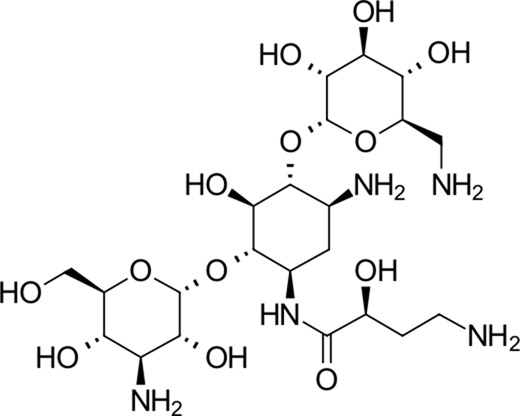	585.6	Amikacin^a^	−37.2	Aminoglycoside antibacterial	No
2	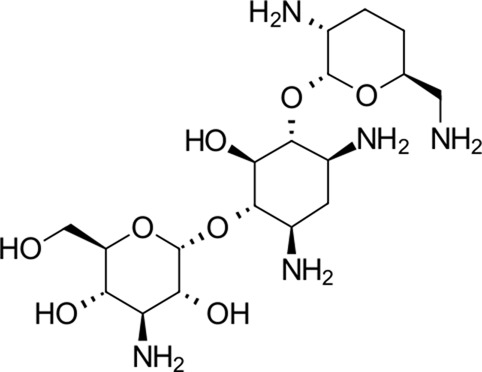	451.5	Dibekacin^a^	−34.5	Aminoglycoside antibacterial	No
3	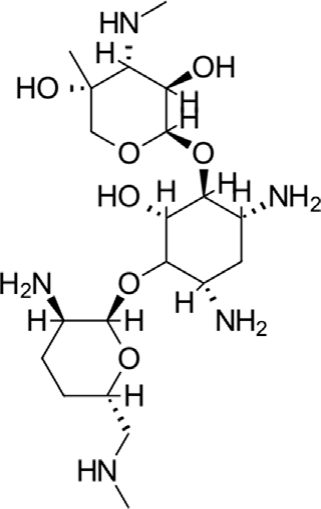	463.6	Micronomicin^a^	−32.3	Aminoglycoside antibacterial	No
4	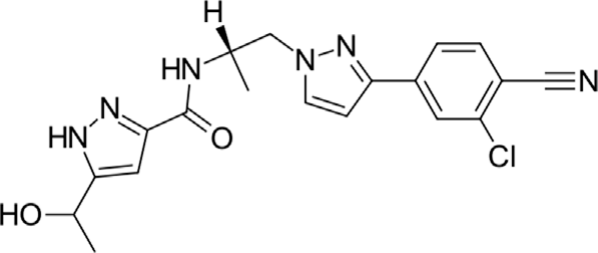	398.9	Darolutamide	−30.2	Anti-androgen (androgen receptor antagonist)	No
5	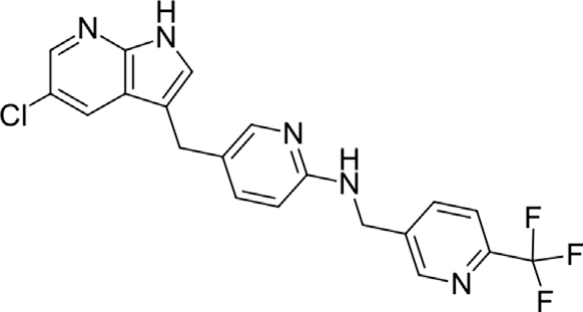	417.8	Pexidartinib	−28.4	Antitumor agent (selective CSF1R inhibitor)	CAD (Slog P = 5.3, RDKit, pKa > 7.4); not a recorded phospholipidosis inducer
6	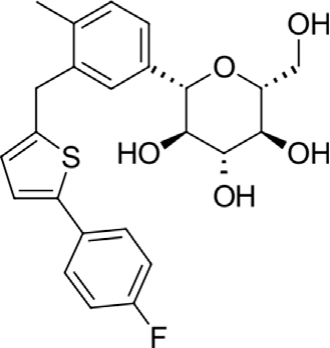	444.5	Canagliflozin	−27.9	Antidiabetic (SGLT2) inhibitor)	No
7	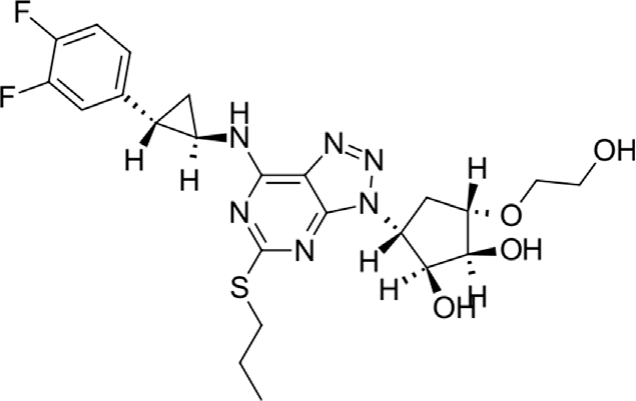	522.6	Ticagrelor	−27.7	Antithrombotic (P2Y12 platelet inhibitor)	No
8	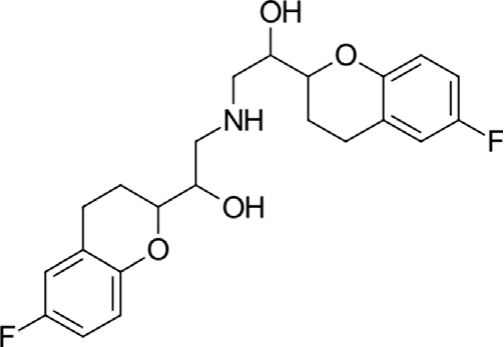	405.4	Nebivolol	−27.5	Beta blocker	No
9	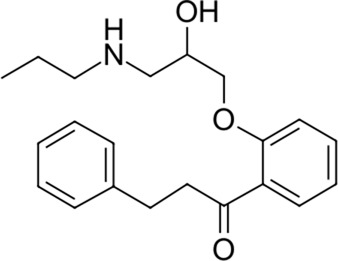	341.4	Propafenone	−27.4	Class 1C antiarrhythmic agent	No
10	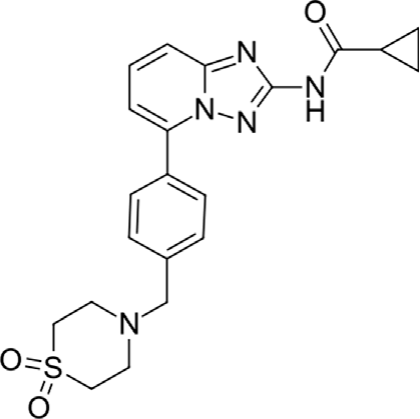	425.5	Filgotinib	−27.3	Antirheumatic (JAK 1 selective inhibitor)	No
11	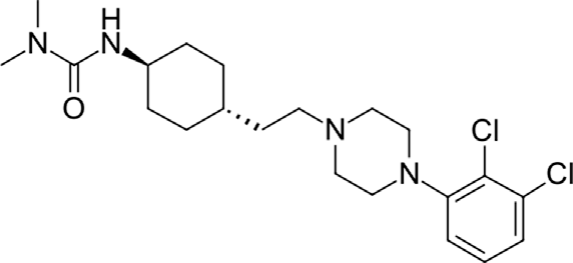	427.4	Cariprazine	−26.4	Atypical antipsychotic (D2 and 5-HT1A modulator)	No

a Flagged compounds are antibacterials and should be treated with care.

b Docking scores in kJ/mol.

c Classification resting on ATC, codes.

d As per Tummino et al.

**TABLE 2:** Identified novel drugs in the repurposing experiment with no prior reported 3CL^pro^ activity data.

No.	Structure	Mr (g/mol)	Name (INN)	CmDock docking score ^a^	Classification ^b^	Phospholipidosis potential *in silico* ^c^
12	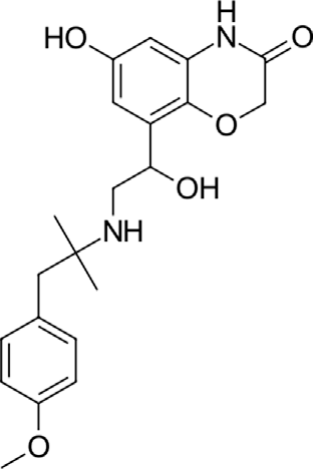	386.5	Olodaterol	−25.8	Beta2-adrenergic agonist	No
13	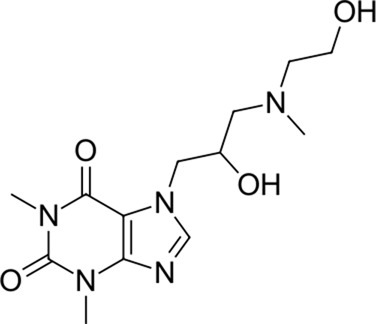	311.3	Xanthinol	−24.5	Vasodilatator	No
14	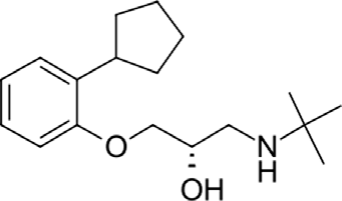	291.4	Penbutolol	−24.4	Beta-adrenergic antagonist	No
15	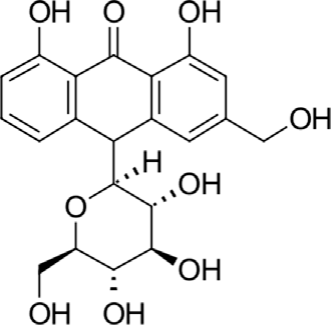	418.4	Alloin	−24.1	Not classified, exp.	No
16	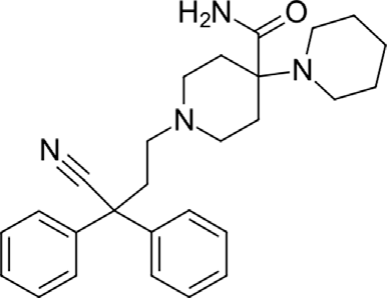	430.6	Piritramide	−23.9	Synthetic opioid	No
17	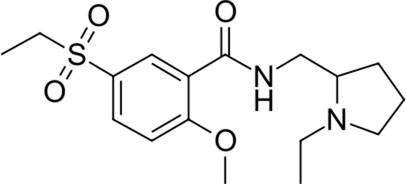	354.5	Sultopride	−23.8	Neuroleptic	No
18	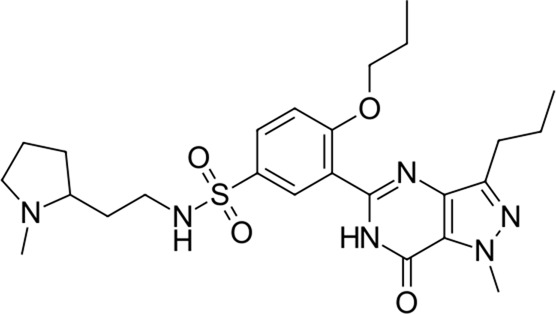	516.7	Udenafil	−23.4	PDE5 inhibitor	No
19	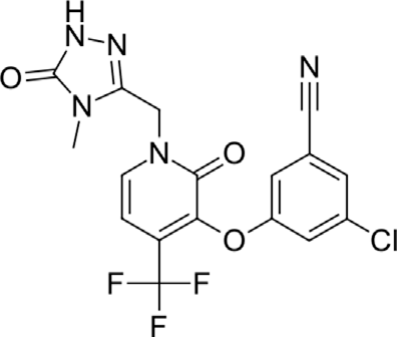	425.7	Doravirine	−22.9	Non-nucleoside reverse transcriptase inhibitor	No
20	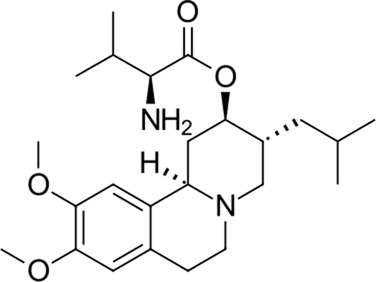	418.6	Valbenazine	−22.7	Monoamine transporter 2 inhibitor	No
21	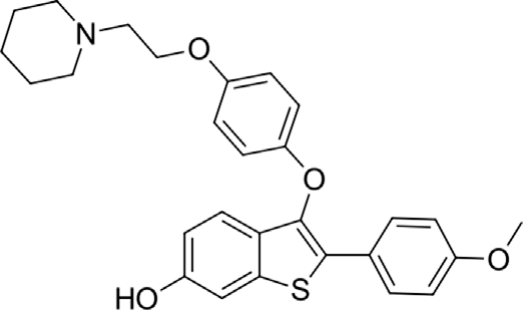	475.6	Arzoxifene	−22.7	Not classified, exp.	CAD (SlogP = 5.5, RDKit, pKa> 7.4); not a recorded phospholipidosis inducer
22	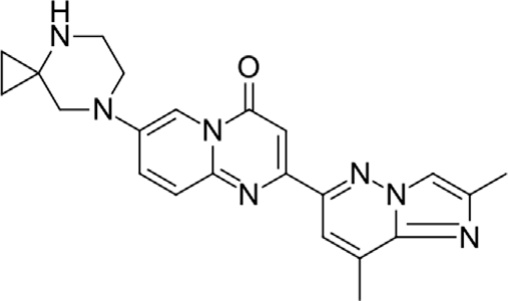	401.5	Risdiplam	−22.4	mRNA splicing modifier	No

a Docking scores in kJ/mol.

b Classification resting on ATC, codes.

c As per Tummino et al.

From the calculated conformations of all 27 compounds, we generated ranked lists of CANDOCK docking scores with all targets from the viral protein database or with targets from the *Coronaviridae* family of proteins (Figure 3). The fingerprint for a single molecule was then generated using the union of the 10 highest-scoring targets from all molecules in the study to obtain comparable fingerprints of equal length. The highest-scoring targets were carefully examined for organisms and protein families. The lists of CANDOCK scores obtained in this way can be used as fingerprints for each compound, and the results are presented as heatmaps for analysis. The authors acknowledge the limitations of the inverse docking method in accurately identifying targets (yet the method is validated as referenced) but postulate that this approach can be used to compare a range of compounds and infer on their structural properties based on *in silico* interactions with a large number of prepared targets or even pharmacophore models. This simple approach can also pave the way for the development of further *in silico* selectivity methods (Figure 3).

## Results and discussion

### Virtual screening of the FDA-approved drug library

To identify accessible hit compounds with potentially favorable physicochemical properties and suitable downstream properties for biological evaluation, rigorous post-docking filtering was performed to screen out pan-assay interference compounds (PAINS; Shoichet, 2006; Baell and Holloway, 2010; Saubern et al., 2011), aggregators (Irwin et al., 2015; to obtain compounds ready for biological evaluation *in vitro*), and structures with reactive functional groups (REOS; to prioritize toward non-covalent inhibitors; Walters et al., 1998; Zhu et al., 2013). The KNIME software with RDKit nodes was applied to compare all structures in the library with the selection of SMARTS-formatted PAINS, aggregator, and REOS libraries and remove flagged matches. The 10 highest-scoring compounds were selected with the Z-score cutoff of −2.5, clustered, and examined in detail (Table 1).

**FIGURE 4:**
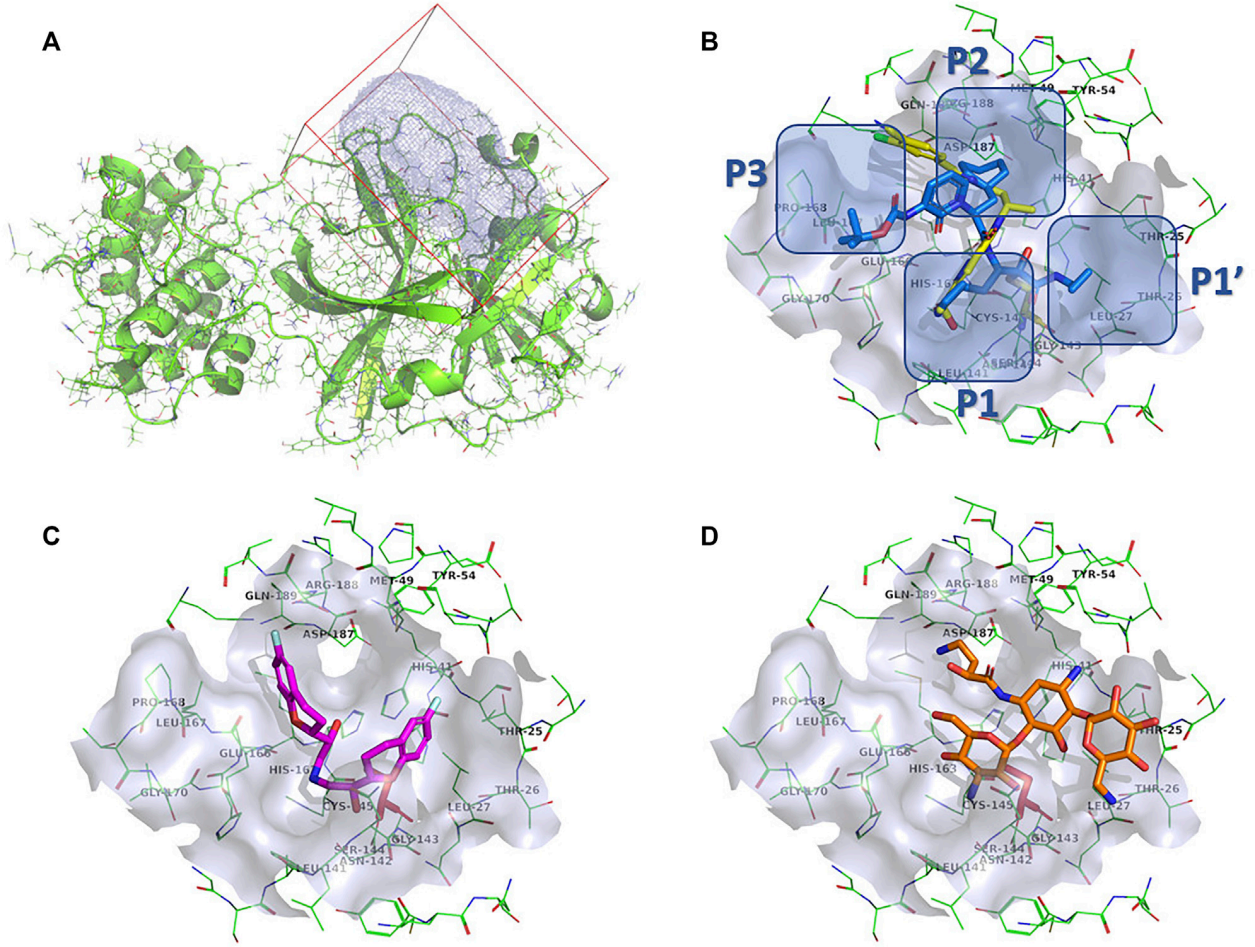
**(A)**: Prepared 3CL^pro^ docking target in the green cartoon model with docking volume highlighted in blue mesh representation. **(B)**: Calculated docking pose of darolutamide shown in the yellow stick model superposed on the OEW reference ligand in the blue stick model. Emphasized are 3CL^pro^ individual binding pockets. **(C)**: Calculated docking pose of nebivolol in the stick model colored magenta. **(D)**: Calculated docking pose of amikacin in the orange-colored stick model. The 3CL^pro^ protein is shown in a green-colored line model, with the active site surface in gray and catalytic Ser144 highlighted in a red-colored stick model.

The identified top-scoring repurposing candidates belong to nine distinct therapeutic classes according to ATC and are detailed in Table 1, with the top three compounds belonging to a group of aminoglycoside antibacterials. Indeed, antibacterial compounds have been proposed as SARS-CoV-2-repurposing candidates, but we would like to highlight their value for the therapy of acquired COVID-19 bacterial coinfections rather than their role as direct-acting antiviral agents (Lai et al., 2020). Moreover, their high scaffold decoration allows for additional target contacts in a typical docking experiment, and we urge the reader to be aware of this fact and interpret the results with this in mind (Meyer-Almes, 2020; Gyselinck et al., 2021). We report that all compounds undergo non-covalent interactions and mainly occupy two different binding poses with classical P1-P2 pocket occupation (Figure 4B; dibekacin, micronomicin, darolutamide, propafenone) or P1′-P2 pocket occupation (Figure 4C), exemplified by pexidartinib, canagliflozin, nebivolol, and filgotinib. The first compound, amikacin, is slightly larger with a MW of 585.6 g/mol and occupies all three P1′-P1-P2 pockets of the active site of 3CL^pro^. The predicted bound conformations for the first 10 hit compounds are analogous with key contacts Thr25, Leu27, Gly143, Ser144, Cys145, His163, His164, Met165, Glu166, Asp187, Thr190, Gln189, and Gln192 at the active site of 3CL^pro^ (Figure 4).

Our top-scoring compounds have also been previously reported and biologically evaluated against 3CL^pro^ (Kumar et al., 2020). Results of extensive screening campaign can also be observed in the ChEMBL database under the comprehensive assay (SARS-CoV-2 3CL-Pro protease inhibition percentage at 20 µM by a FRET kind of response from peptide substrate) with more than 8,700 data points at the time of writing. We report these compounds as 3CL^pro^ hit identification control and support their repurposing research *via* further *in silico* inverse docking fingerprint analysis. Nevertheless, we are compelled to report further 11 potential 3CL^pro^ inhibitors (compounds 12–22) identified by our virtual screening experiment without any existing 3CL^pro^ activity data (CHEMBL4495582, CHEMBL4495583) that can be of use to the SARS-CoV-2 repurposing research (Table 2).

**FIGURE 5:**
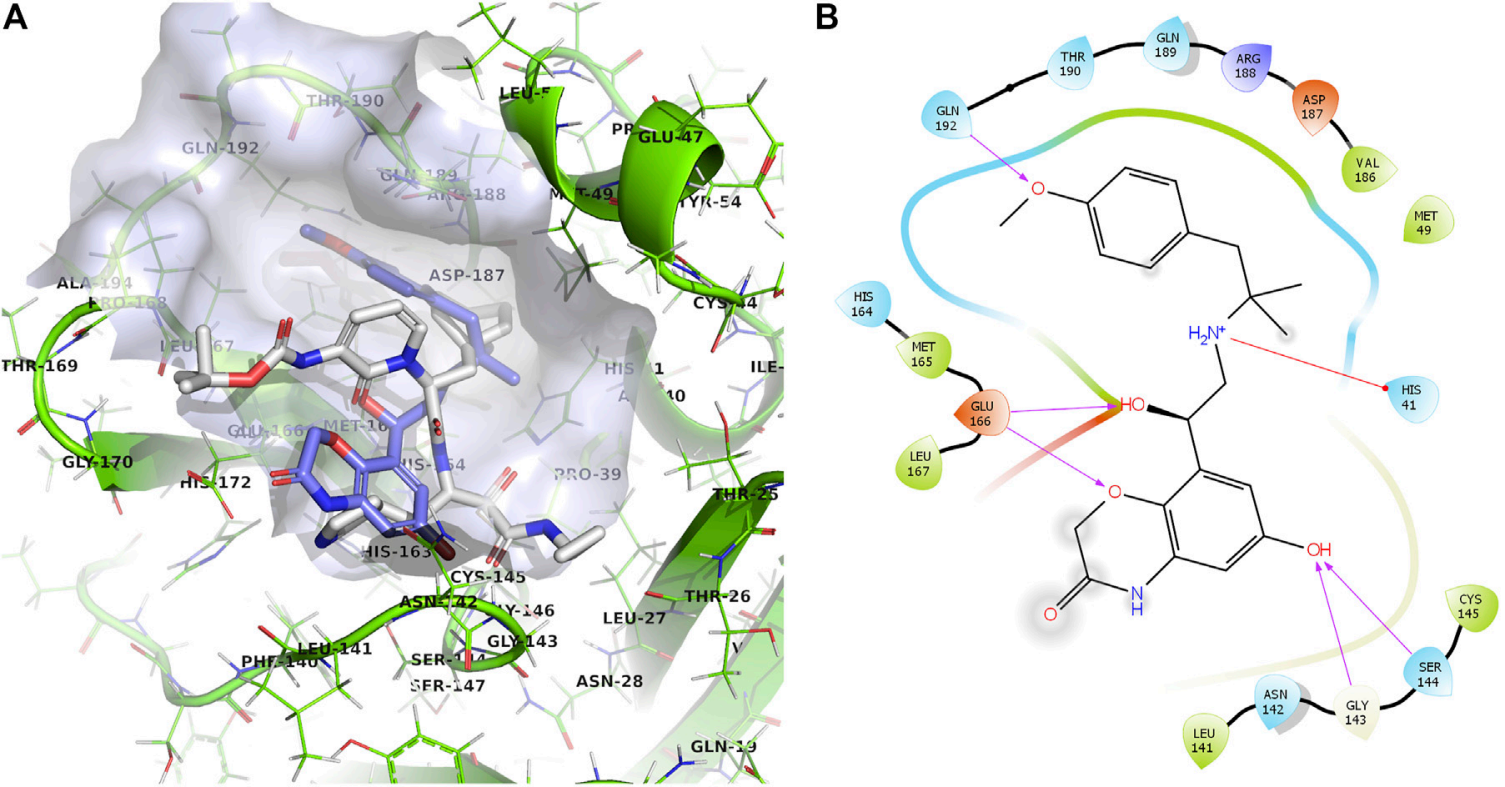
**(A)**: Prepared 3CL^pro^ docking target in the green cartoon model with labeled active site residues with calculated docking pose of olodaterol (12) shown in the light-blue stick model superposed on the OEW reference ligand in the white stick model. The binding site surface around the ligand is emphasized in transparent blue-gray color. **(B)**: 2D projection of the calculated docking pose of olodaterol (12) indicating key residues in vicinity and ligand-binding site interactions.

New 11 reported hit compounds (12–22) belong to eight distinct therapeutic classes according to ATC with two compounds (15, 21) not yet classified as being experimental agents. Examining the ChEMBL database, no compound possesses activity on coronavirus targets as of yet. In this hit list, we also do not report antibacterials, as they are not favorable for drug repurposing causing additional selection pressure and antibacterial resistance problems (Andersson et al., 2016). The list represents small molecules suitable for further biological evaluation on SARS-CoV-2 3CL^pro^ without phospholipidosis potential as identified by Tummino et al. (2021). Majority of compounds occupy the P1-P2 pocket at the 3CL^pro^ active site near Cys145 (Figure 5A) analogous to the PDB ID: 6Y7M OEW reference ligand. Highest-ranking compound **12** (olodaterol) thus makes hydrophobic contacts with Met165 and Glu166; hydrogen bonds with Gly143, Ser144, Cys145, Glu166, and Gln192; and cation-π interactions toward His41 (Figure 5; additional details in Supplementary Material).

Moody et al. also conducted a virtual screening experiment on 3CL^pro^ and reported a list of 56 top scoring candidates on 3CL^pro^, selected according to the highest S-score (between −9.95 and −4.24). After biological evaluation, Moody et al. proposed six repurposing candidates with IC_50_ values ranging from 21.5 to 75.5 µM, namely, micafungin (4th; −9.60 S-score), an antifungal agent of the echinocandin class, ombitasvir (12th; −8.97 S-score), an HCV NS5A inhibitor, boceprevir (23rd; −8.42 S-score), a representative of an HCV protease inhibitor, ivermectin (32nd; −7.74), an antiparasitic that binds to glutamate-gated chloride channels found in invertebrate nerve and muscle cells, tipranavir (35th; −7.48 S-score), an HIV protease inhibitor, and paritaprevir (36th; −7.43 S-score), an HCV NS3-4A serine protease inhibitor (Mody et al., 2021). The observed differences in the hit list can be attributed to the screening software. Moody et al. used the MOE software suite with a different approach to scoring (S-score calculated using the London dG score for placement and the GBVI/WSA dG score for pose refinement). To illustrate the difference, micafungin, an antifungal drug with a molecular mass of 1,270.28 g/mol, was not included, while ombitasvir, boceprevir, ivermectin, tipranavir, and paritaprevir were scored by CmD as −7.81, −7.35, −9.29, −20.10, and −18.27, respectively. However, all reported compounds by Moody et al. are thus reported to have activity on 3CL^pro^, similarly as we demonstrated by our screening effort.

### Inverse docking of repurposing candidates

**FIGURE 6:**
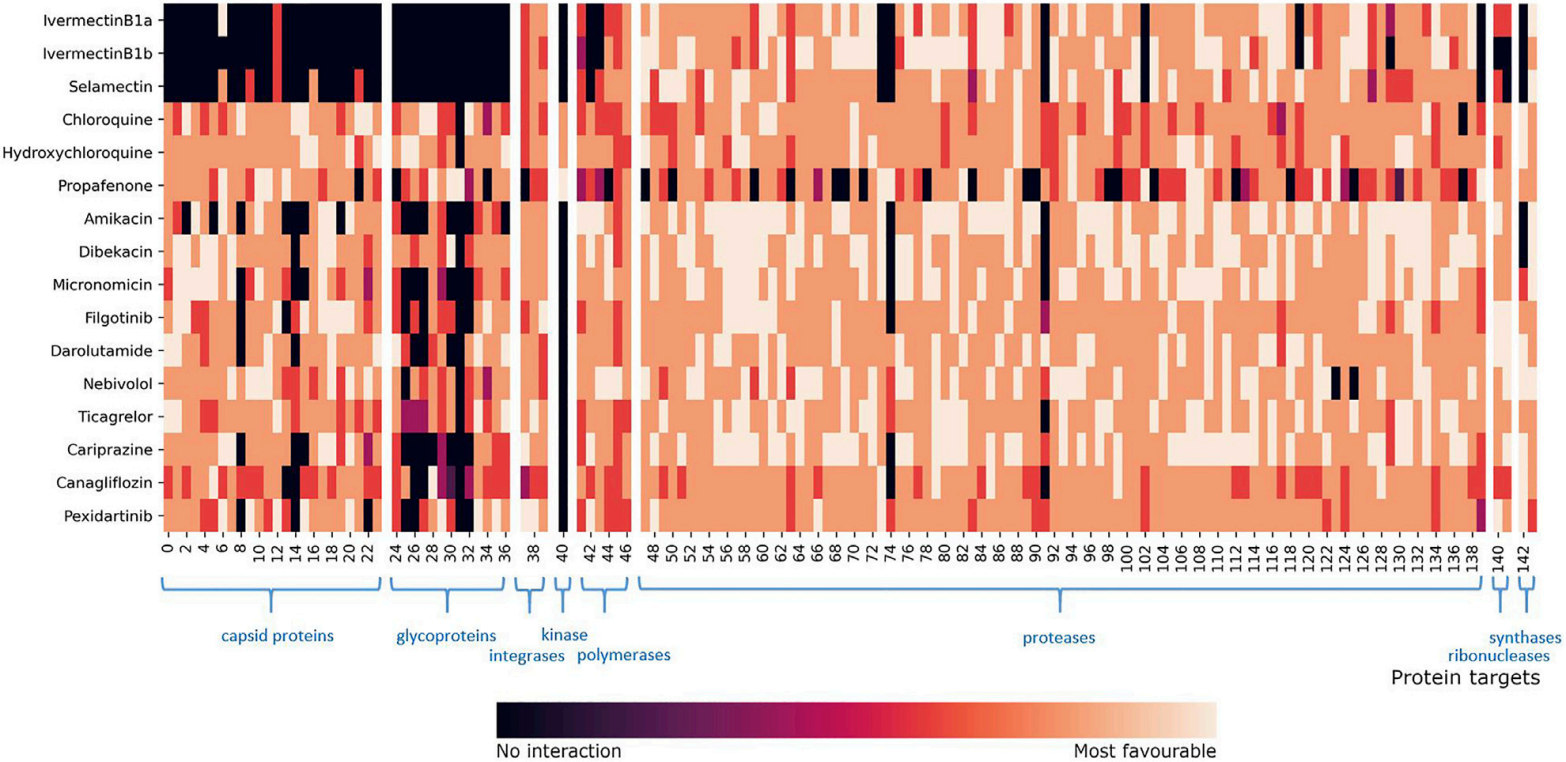
Heatmap representation of compound fingerprints for the complete viral target database (11,640 proteins) of all hit compounds. The total number of combined targets is 144, and the list of PDB IDs is found in Supplementary Table S20. The values shown in the heatmap were calculated and colored according to the normalized docking score for each compound. Interval 0 means that the compound did not interact with the target, while interval 5 means that the compound had a most favorable docking score. Protein targets are grouped according to their class and classes emphasized in blue color.

**FIGURE 7:**
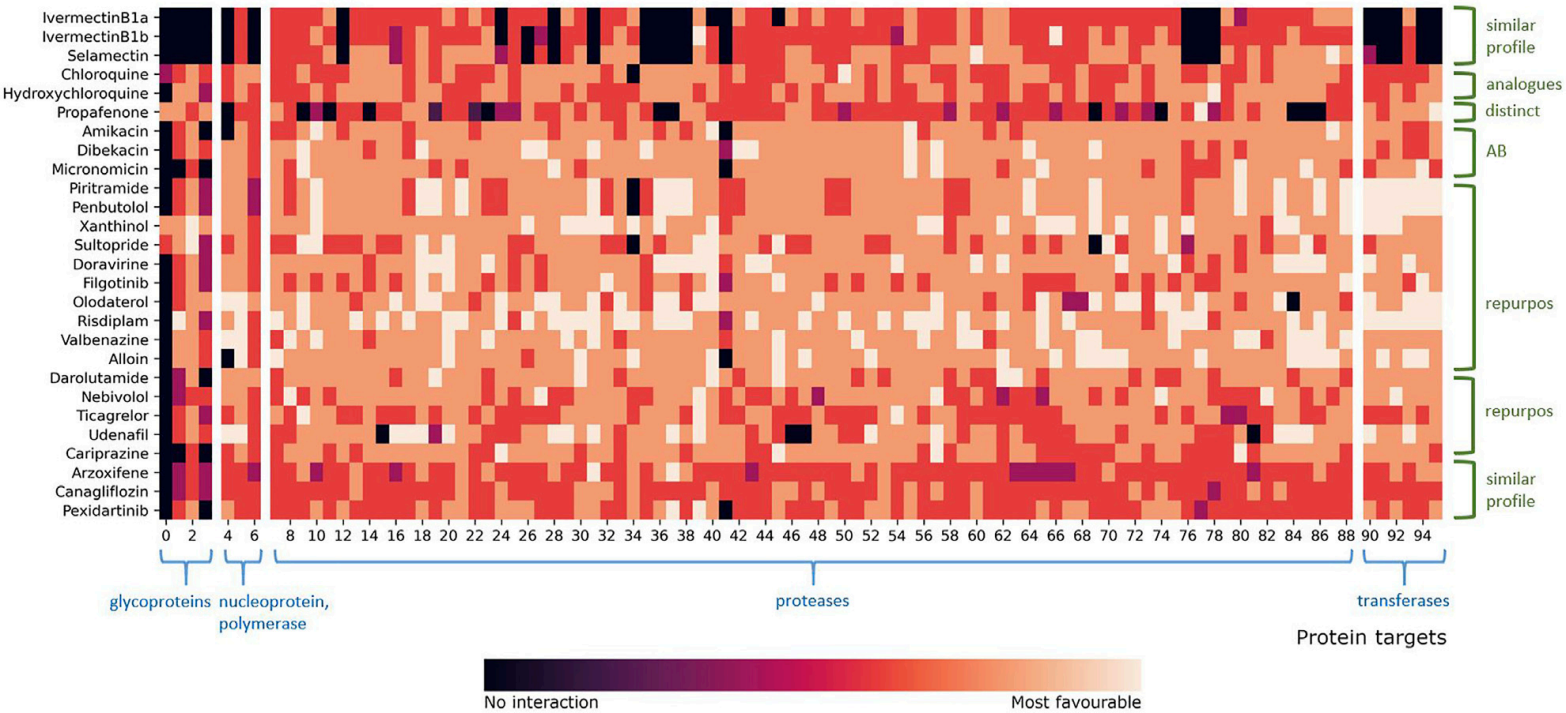
Heatmap representation of compound fingerprints for the *Coronaviridae* database subset (836 proteins) for all compounds. The total number of combined targets is 96, and the list of PDB IDs is found in Supplementary Table S21. The values shown in the heatmap were calculated and colored according to the normalized docking score for each compound. Interval 0 means that the compound did not interact with the target, while interval 5 means that the compound had a most favorable docking score. Protein targets are grouped according to their class and classes emphasized in blue color. Fingerprint profile similarities are colored green. Two “repurpose” subsets represent two different fingerprint profiles within the repurpose compounds group.

To identify the protein targets to which our identified hit compounds express the highest binding potential, we performed rigorous post-docking filtering and expressed only top-scoring targets for reader benefit. We recorded the lists of proteins for each compound and considered only the highest-scoring targets (Table 3). Complete target protein lists are found in Supplementary Tables S1–S3. To generate inverse docking target interaction fingerprints, we combined the 10 highest-scoring viral protein targets of each compound into one list (example for amikacin: 2c86A: 0; 2gecB: −51.6166; 2q6fB: −73.2794; 3cl5A: −57.3933; 4f49A: −68.9848; 4h14A: 0; 4pt5A: −59.4445; 4rezA: −51.841; 4wurA: 0; 5c3nA: 0; 5gwzB: −75.8171; 5hyoA: 0; 5jilA: −67.1155; 5nfyA: −60.2988; 6jijA: 0; 6l5tA: −45.6625; 6nozA: −65.0647; 6qfyA: 0; 6u7hB: −39.7457; 6u7kB: −70.3975; 6y3yA: −35.5868; 6zgfC: 0). We removed duplicate proteins and included docking scores for all targets on the list for each compound. A target interaction space was then plotted as a heatmap as shown in Figure 6. The same procedure was used for the *Coronaviridae* protein subset experiment (Figure 7). Complete lists of successfully docked *Coronaviridae* family targets for each compound are found in Supplementary Tables S4–S19.

**TABLE 3:** Identified viral proteins with the highest scores in the inverse molecular docking experiment.

Ligand	Docking score (arbitrary units)	Organism	Protein name
Amikacin	−101.392	Human immunodeficiency virus-1 HIV-1	HIV-1 protease
	−100.722	Feline immunodeficiency virus (isolate Petaluma)	Retropepsin
	−99.3221	Human rhinovirus type 5	Rhinovirus B5 VP4
	−98.5941	Human immunodeficiency virus type 1 (BRU ISOLATE)	HIV-1 protease
Canagliflozin	−71.9432	Paramecium bursaria Chlorella virus PBCV-1	Probable thymidylate synthase
	−64.0748	Human immunodeficiency virus type 2 (ISOLATE ROD)	Protease
	−62.3561	Human immunodeficiency virus type 1 (BRU ISOLATE)	HIV-1 protease
	−61.7061	Southampton virus (serotype 3)	Thiol protease P3C
Cariprazine	−77.0656	Human immunodeficiency virus type 2, HIV-2	HIV-2 protease
	−75.9674	Human immunodeficiency virus-1 HIV-1	Protease
	−74.7457	Human immunodeficiency virus type 1 (BRU ISOLATE)	Protease
	−74.6709	Human immunodeficiency virus type 1 (ARV2/SF2 ISOLATE)	Protease
Chloroquine	−69.6592	Paramecium bursaria Chlorella virus PBCV-1	Probable thymidylate synthase
	−62.3342	Human enterovirus EV68	Capsid protein VP1
	−61.6339	Human enterovirus EV68	Viral protein 1
	−61.5787	Human immunodeficiency virus-1 HIV-1	HIV-1 protease
Darolutamide	−73.0195	Human rhinovirus type 5	Rhinovirus B5 VP4
	−71.54	Human immunodeficiency virus type 1 (BH10 ISOLATE)	Reverse transcriptase/ribonuclease H
	−70.4064	Dengue virus type 1 Singapore/S275/1990	Fusion protein of nonstructural protein 2B and nonstructural protein 3
	−69.5576	ZIKV	NS2B-NS3 protease
Dibekacin	−82.4989	Human immunodeficiency virus type 1 (BRU ISOLATE)	HIV-1 protease
	−82.4278	Human immunodeficiency virus-1 HIV-1	Protease
	−82.1087	Adeno-associated virus type 3B	Capsid protein VP1
	−81.8136	Influenza virus type A	Hemagglutinin HA2 chain
Filgotinib	−76.1691	Human rhinovirus type 5	Rhinovirus B5 VP4
	−71.0266	Human immunodeficiency virus type 1 (BH10 ISOLATE)	Reverse transcriptase/ribonuclease H
	−70.9686	Human immunodeficiency virus type 1 (NEW YORK-5 ISOLATE)	Capsid protein p24
	−69.6959	Human immunodeficiency virus-1 HIV-1	gp120
Hydroxychloquine	−75.4441	Human enterovirus CVA10	Capsid protein VP1
	−70.6169	Human enterovirus CVA10	Capsid protein VP1
	−70.4093	Human enterovirus CVA16	VP1
	−69.3791	Human immunodeficiency virus-1 HIV-1	Protease retropepsin
Ivermectin B1a	−111.313	Human immunodeficiency virus-1 HIV-1	Protease
	−108.816	Human immunodeficiency virus type 1 (Z2/CDC-Z34 ISOLATE)	Protease
	−104.269	Human immunodeficiency virus-1 HIV-1	Protease
	−104.208	Human immunodeficiency virus-1 HIV-1	Protease
Ivermectin B1b	−107.924	Human immunodeficiency virus-1 HIV-1	HIV-1 protease
	−106.019	Human immunodeficiency virus-1 HIV-1	Protease retropepsin
	−105.357	Human immunodeficiency virus-1 HIV-1	HIV-1 protease
	−104.804	Human immunodeficiency virus-1 HIV-1	HIV-1 protease
Micronomicin	−88.8098	Human immunodeficiency virus-1 HIV-1	POL polyprotein
	−88.7453	Human immunodeficiency virus-1 HIV-1	Protease
	−86.5514	Adeno-associated virus type 3B	Capsid protein VP1
	−86.012	Human immunodeficiency virus-1 HIV-1	Protease
Nebivolol	−71.5908	Human immunodeficiency virus-1 HIV-1	HIV-1 capsid protein
	−71.0465	Human enterovirus EV68	Viral protein 1
	−70.2915	DG-75 Murine leukemia virus	Gag-pro-pol polyprotein
	−69.8426	Human immunodeficiency virus-1 HIV-1	Protease
Pexidartinib	−61.0336	Adeno-associated virus type 6	Capsid protein VP1
	−54.6906	Human immunodeficiency virus-1 HIV-1	Protease
	−54.0666	Bovine respiratory syncytial virus BRSV	Fusion glycoprotein F0, Fibritin
	−53.3484	Human immunodeficiency virus type 1 (NEW YORK-5 ISOLATE)	Capsid protein p24
Propafenone	−69.6736	Human enterovirus EV68	Capsid protein VP1
	−67.4871	Human enterovirus EV68	Viral protein 1
	−67.0906	Influenza virus type A	Hemagglutinin HA2 chain
	−66.5011	Human immunodeficiency virus type 1 (BRU ISOLATE)	Protease
Selamectin	−89.903	ZIKV	NS2B-NS3 protease
	−86.8758	Human immunodeficiency virus-1 HIV-1	HIV-1 protease
	−85.6238	Human immunodeficiency virus-1 HIV-1	HIV-1 protease
	−85.1895	Human immunodeficiency virus-1 HIV-1	HIV-1 protease
Ticagrelor	−83.1521	Human immunodeficiency virus type 2, HIV-2	HIV-2 protease
	−79.2021	Human immunodeficiency virus-1 HIV-1	HIV-1 protease
	−78.8308	Human immunodeficiency virus-1 HIV-1	HIV-1 protease
	−78.4946	Human echovirus E11	Echovirus 11 coat protein vp1

An examination of our inverse docking fingerprints reveals that they are unique to each compound, suggesting that subtle differences in compound conformational space and scaffold decoration (chloroquine and hydroxychloroquine, ivermectin B1a and B1b) have a profound effect on the scope of the target reach, making them very useful for the future development of models (Belyaeva et al., 2021; Kumar Das et al., 2021) and machine learning (D’Souza et al., 2020). Namely, the compound’s ability to conform to a particular set of protein targets is used as ligand structural information that can be of use also when comparing a set of diverse compounds (e.g., can two compounds, even structurally dissimilar conform to a larger set of protein targets and produce favorable binding modes?)

The most studied target of SARS-CoV-2 is the 3CL^pro^, and it is actually postulated as a target (Mody et al., 2021) for the previously reported repurposed drug ivermectin (Kaur et al., 2021; Kern et al., 2021; Nardelli et al., 2021). As is shown in Table 3, the major targets of ivermectin B1a and B1b, as well as the structurally similar selamectin, were identified as proteases. We can support previous reports of ivermectin targeting (Eweas et al., 2021), while with our approach we are able to also place the compounds in the context of other potential therapeutic targets (Zaidi and Dehgani-Mobaraki, 2021; Figure 6). The fingerprint profiles of ivermectins and selamectin are also similar (Figure 6). In comparison, the targets of chloroquine and hydroxychloroquine, calculated by inverse docking, are not proteases and their fingerprints differ significantly. Following the initial reports (Garcia-Cremades et al., 2020) on their efficacy and viral targeting (Adeoye et al., 2020), we are unable to report similar observations and we can treat these compounds (and their fingerprint profile)as negative control examples, as recently reported (Shah, 2021). The fingerprints described can thus be of use in an extensive inverse docking fingerprint screening of a large database of compounds to identify compounds with a similar fingerprint profile.

Upon inspection of the inverse docking fingerprints and top docking poses for all hit compounds 1-11 from the first virtual screening experiment with FDA-approved drugs, all drugs except filgotinib have high docking potential against proteases (Table 3). Beforehand, Ribone et al. (2021) identified protease (PLpro) as the main target of hydroxychloroquine, while further suggesting a human protein PIKfyve as a main target of pexidartinib. Therefore, we can support the potential of the identified FDA-approved drugs to inhibit SARS-CoV-2 3CL^pro^ and also treat the compounds as controls (experimental 3CL^pro^ activity; CHEMBL4495582, CHEMBL4495583). For example, we can postulate that the diverse set of compounds can produce favorable binding poses at the active site of HIV proteases so this set of compounds can access the conformational space that is similar or presents a similar set of transient pharmacophoric elements. This identified potential (similarity in accessing other targets) of a set of studied compounds can then be leveraged in further experimental and repurposing experiments to access novel chemical space.

We fingerprinted the compounds 1–22 using the *Coronaviridae* family subgroup in a similar manner. The proteins of compounds 1–11 controls (experimental 3CL^pro^ activity) are used for fingerprint comparison with novel repurposing candidates without prior experimental information 12–22 (Figure 7). The highest-scoring *Coronaviridae* targets belong to the protease family (Supplementary Tables S4–S19), making all newly reported compounds except arzoxifene, a potential SARS-CoV-2 3CL^pro^ inhibitor that should be evaluated further (Figure 7).

If we compare the fingerprint of propafenone to the other repurposing candidates, we can see that it has significantly more unfavorable targets than other compounds (Figure 7). We also observed that propafenone possesses a distinct fingerprint profile in comparison to other studied drugs. From this, one can postulate that propafenone could possess different mechanisms of action to the other compounds. Each fingerprint is unique; nevertheless, global trends emerge when examining multiple compounds in parallel (Figure 7). Namely, pexidartinib and arzoxifene show similar fingerprint profiles (Figure 7; bottom) which indicates less favorable binding to majority of targets, and both are incidentally also flagged as CAD compounds with phospholipidosis potential (Tables 2, 3). This information can be leveraged in further full-scale inverse docking screening, as stated beforehand.

To further elaborate on inverse docking fingerprints, two case studies were conducted. First is a case study of a fingerprinting target, papain-like protease (PL^pro^). This target was selected as a random representative of the most abundant protease class. Case compounds were also randomly selected, one from each fingerprint-like group: dibekacin, hydroxychloroquine, olodaterol, pexidartinib, and udenafil. As detailed in Supplementary Figure S1, all five compounds bind into the same binding pocket, validating the target database (DeLano, 2002, Adasme et al., 2021; Pettersen et al., 2004). More detailed analysis of the binding interactions shows that udenafil has hydrophobic interactions with Asp165 and Tyr269; hydrogen bonds with Leu163, Tyr265, Tyr269, and Tyr274; and salt bridges to Asp165 and Asp303, suggesting that it may have a more favorable binding potential as a repurposing candidate compared to pexidartinib, which shows hydrophobic interactions with Leu163, Gln270, and π-π stacking with Tyr269. This observation is also immediately apparent from the fingerprint profile in Figure 7. Second, a case study of the fingerprint compound olodaterol is presented. This compound was chosen because it is the highest-scoring repurposing candidate for which data on 3CL^pro^ activity are not yet available. We investigated the binding positions of the repurposing candidate to different protein targets included in its inverse docking fingerprint. The representatives of the target proteins were randomly selected from each protein target class: 3C-like protease and papain-like protease, NSP16 transferase, and spike protein. A detailed analysis of the binding interactions (Supplementary Material) shows that the repurposing candidate has the best docking results and the most favorable binding conformations toward proteases compared to the other targets (3CL^pro^: hydrogen bonds with Leu141, Gly143, Ser144, Cys145, and Glu166; PL^pro^: hydrophobic interactions with Asp165, Tyr265, and Tyr269; and hydrogen bonds with Asp165, Arg167, Glu168, Tyr269, and Gln192). The observations thus support our findings and suggestions on the new SARS-CoV-2 repurposing candidates.

## Conclusion

We conducted a thorough virtual screening experiment on SARS-CoV-2 3CL^pro^ using CmDock software and a database of FDA-approved drugs and identified the highest-scoring drug repurposing candidates. All newly reported candidates (12–22) are readily available and show favorable non-covalent interactions at the active site of 3CL^pro^, and all newly identified hit compounds show a low propensity for phospholipidosis, with the exception of arzoxifene. The latter compound belongs to a typical cathionic amphiphilic drug scaffold but has not been identified as a known inducer of phospholipidosis. Furthermore, in reviewing the inverse docking fingerprints, we found that the majority of the identified FDA-approved repurposing candidates have favorable docking scores against Coronaviridae family proteases and viral proteases in general. In addition, we support current *in silico* studies on 11 top-scoring compounds (1–11) as well as on antihelminthic class member compounds. We postulate the application of this approach to future inverse docking fingerprint screening experiments to investigate the selectivity of compound interaction *in silico* and to identify similarly interacting compounds in large protein databases.

## Data Availability

The original contributions presented in the study are included in the article/Supplementary Material; further inquiries can be directed to the corresponding authors.

## Abbreviations

CAD, cationic amphiphilic drug; FDA, Food and Drug Administration; IMPDH2, inosine-5′-monophosphate dehydrogenase 2; RNA, ribonucleic acid; SARS, severe acute respiratory syndrome; +ss, positive single stranded.

## References

[B1] Abate C. Niso M. Abatematteo F. S. Contino M. Colabufo N. A. Berardi F. (2020). PB28, the Sigma-1 and Sigma-2 Receptors Modulator With Potent Anti-SARS-CoV-2 Activity: A Review About its Pharmacological Properties and Structure Affinity Relationships. Front. Pharmacol. 11, 589810. 10.3389/fphar.2020.589810 33364961 PMC7750835

[B2] Adasme M. F. Linnemann K. L. Bolz S. N. Kaiser F. Salentin S. Haupt V. J. (2021). PLIP 2021: Expanding the Scope of the Protein-Ligand Interaction Profiler to DNA and RNA. Nucleic Acids Res. 49 (W1), W530–W534. 10.1093/nar/gkab294 33950214 PMC8262720

[B3] Adeoye A. O. Oso B. J. Olaoye I. F. Tijjani H. Adebayo A. I. (2020). Repurposing of Chloroquine and Some Clinically Approved Antiviral Drugs as Effective Therapeutics to Prevent Cellular Entry and Replication of Coronavirus. J. Biomol. Struct. Dyn. 39 (10), 3469–3479. 10.1080/07391102.2020.1765876 32375574 PMC7232887

[B4] Amanat F. Krammer F. (2020). SARS-CoV-2 Vaccines: Status Report. Immunity. 52 (4), 583–589. 10.1016/j.immuni.2020.03.007 32259480 PMC7136867

[B5] Anand K. Ziebuhr J. Wadhwani P. Mesters J. R. Hilgenfeld R. (2003). Coronavirus Main Proteinase (3CL Pro ) Structure: Basis for Design of Anti-SARS Drugs. Science. 300 (5626), 1763–1767. 10.1126/science.1085658 12746549

[B6] Andersson D. I. Hughes D. Kubicek-Sutherland J. Z. (2016). Mechanisms and Consequences of Bacterial Resistance to Antimicrobial Peptides. Drug Resist. Updates. 26, 43–57. 10.1016/j.drup.2016.04.002 27180309

[B7] Badgujar K. C. Badgujar V. C. Badgujar S. B. (2020). Vaccine Development Against Coronavirus (2003 to Present): An Overview, Recent Advances, Current Scenario, Opportunities and Challenges. Diabetes Metab. Syndr. Clin. Res. Rev. 14 (5), 1361–1376. 10.1016/j.dsx.2020.07.022 PMC7371592 32755836

[B8] Baell J. B. Holloway G. A. (2010). New Substructure Filters for Removal of pan Assay Interference Compounds (PAINS) From Screening Libraries and for Their Exclusion in Bioassays. J. Med. Chem. 53 (7), 2719–2740. 10.1021/jm901137j 20131845

[B9] Bakowski M. A. Beutler N. Wolff K. C. Kirkpatrick M. G. Chen E. Nguyen T. T. H. (2021). Drug Repurposing Screens Identify Chemical Entities for the Development of COVID-19 Interventions. Nat. Commun. 12 (1), 1–14. 10.1038/s41467-021-23328-0 34083527 PMC8175350

[B10] Belyaeva A. Cammarata L. Radhakrishnan A. Squires C. Yang K. D. Shivashankar G. V. (2021). Causal Network Models of SARS-CoV-2 Expression and Aging to Identify Candidates for Drug Repurposing. Nat. Commun. 12 (1), 1024–1113. 10.1038/s41467-021-21056-z 33589624 PMC7884845

[B11] Chen C. Z. Xu M. Pradhan M. Gorshkov K. Petersen J. D. Straus M. R. (2020a). Identifying SARS-CoV-2 Entry Inhibitors Through Drug Repurposing Screens of SARS-S and MERS-S Pseudotyped Particles. ACS Pharmacol. Transl. Sci. 3 (6), 1165–1175. 10.1021/acsptsci.0c00112 33330839 PMC7586456

[B12] Chen W. H. Strych U. Hotez P. J. Bottazzi M. E. (2020b). The SARS-CoV-2 Vaccine Pipeline: An Overview. Curr. Trop. Med. Rep. 7, 61–64. 10.1007/s40475-020-00201-6 PMC7094941 32219057

[B13] Chen S.-J. (2014). A Potential Target of Tanshinone IIA for Acute Promyelocytic Leukemia Revealed by Inverse Docking and Drug Repurposing. Asian Pac. J. Cancer Prev. 15 (10), 4301–4305. 10.7314/apjcp.2014.15.10.4301 24935388

[B14] Chiou W.-C. Hsu M.-S. Chen Y.-T. Yang J.-M. Tsay Y.-G. Huang H.-C. (2021). Repurposing Existing Drugs: Identification of SARS-CoV-2 3C-Like Protease Inhibitors. J. Enzyme Inhib. Med. Chem. 36 (1), 147–153. 10.1080/14756366.2020.1850710 33430659 PMC7808739

[B15] Coomes E. A. Haghbayan H. (2020). Favipiravir, an Antiviral for COVID-19? J. Antimicrob. Chemother. 75 (7), 2013–2014. 10.1093/jac/dkaa171 32417899 PMC7239147

[B16] Cortegiani A. Ingoglia G. Ippolito M. Giarratano A. Einav S. (2020). A Systematic Review on the Efficacy and Safety of Chloroquine for the Treatment of COVID-19. J. Crit. Care. 57, 279–283. 10.1016/j.jcrc.2020.03.005 32173110 PMC7270792

[B17] Cui J. Li F. Shi Z.-L. (2019). Origin and Evolution of Pathogenic Coronaviruses. Nat. Rev. Microbiol. 17 (3), 181–192. 10.1038/s41579-018-0118-9 30531947 PMC7097006

[B18] de Oliveira O. V. Rocha G. B. Paluch A. S. Costa L. T. (2021). Repurposing Approved Drugs as Inhibitors of SARS-CoV-2 S-Protein from Molecular Modeling and Virtual Screening. J. Biomol. Struct. Dyn. 39 (11), 3924–3933. 10.1080/07391102.2020.1772885 32448085 PMC7284156

[B19] De P. Chakraborty I. Karna B. Mazumder N. (2021). Brief Review on Repurposed Drugs and Vaccines for Possible Treatment of COVID-19. Eur. J. Pharmacol. 898, 173977. 10.1016/j.ejphar.2021.173977 33639193 PMC7905377

[B20] de Wit E. Van Doremalen N. Falzarano D. Munster V. J. (2016). SARS and MERS: Recent Insights into Emerging Coronaviruses. Nat. Rev. Microbiol. 14 (8), 523–534. 10.1038/nrmicro.2016.81 27344959 PMC7097822

[B21] DeLano W. L. (2002). Pymol: An Open-Source Molecular Graphics Tool. CCP4 Newsl. Protein Crystallogr. 40 (1), 82–92.

[B22] Depolli M. Konc J. Rozman K. Trobec R. Janežič D. (2013). Exact Parallel Maximum Clique Algorithm for General and Protein Graphs. J. Chem. Inf. Model. 53 (9), 2217–2228. 10.1021/ci4002525 23965016

[B23] Dotolo S. Marabotti A. Facchiano A. Tagliaferri R. (2021). A Review on Drug Repurposing Applicable to COVID-19. Brief. Bioinformatics. 22 (2), 726–741. 10.1093/bib/bbaa288 33147623 PMC7665348

[B24] D’Souza S. Prema K. V. Balaji S. (2020). Machine Learning Models for Drug–Target Interactions: Current Knowledge and Future Directions. Drug Discov. Today. 25 (4), 748–756. 10.1016/j.drudis.2020.03.003 32171918

[B25] Eweas A. F. Alhossary A. A. Abdel-Moneim A. S. (2021). Molecular Docking Reveals Ivermectin and Remdesivir as Potential Repurposed Drugs against SARS-CoV-2. Front. Microbiol. 11, 3602. 10.3389/fmicb.2020.592908 PMC7976659 33746908

[B26] Faria N. R. Claro I. M. Candido D. Moyses Franco L. A. Andrade P. S. Coletti T. M. (2021). Genomic Characterisation of an Emergent SARS-CoV-2 Lineage in Manaus: Preliminary Findings. Science 372 (6544), 815–821. 10.1126/science.abh2644 33853970 PMC8139423

[B27] Fillbrunn A. Dietz C. Pfeuffer J. Rahn R. Landrum G. A. Berthold M. R. (2017). KNIME for Reproducible Cross-Domain Analysis of Life Science Data. J. Biotechnol. 261, 149–156. 10.1016/j.jbiotec.2017.07.028 28757290

[B28] Fine J. Konc J. Samudrala R. Chopra G. (2020). CANDOCK: Chemical Atomic Network-Based Hierarchical Flexible Docking Algorithm Using Generalized Statistical Potentials. J. Chem. Inf. Model. 60, 1509–1527. 10.1021/acs.jcim.9b00686 32069042 PMC12034428

[B29] Frisch M. J. Trucks G. W. Schlegel H. B. Scuseria G. E. Robb M. A. Cheeseman J. R. (2016). Gaussian 16 Rev. A.03. Wallingford, UK: Gaussian, Inc.

[B30] Fry C. V. Cai X. Zhang Y. Wagner C. S. (2020). Consolidation in a Crisis: Patterns of International Collaboration in Early COVID-19 Research. PLoS One. 15 (7), e0236307. 10.1371/journal.pone.0236307 32692757 PMC7373281

[B31] Furlan V. Konc J. Bren U. (2018). Inverse Molecular Docking as a Novel Approach to Study Anticarcinogenic and Anti-Neuroinflammatory Effects of Curcumin. Molecules. 23, 3351. 10.3390/molecules23123351 PMC6321024 30567342

[B32] Garcia-Cremades M. Solans B. P. Hughes E. Ernest J. P. Wallender E. Aweeka F. (2020). Optimizing Hydroxychloroquine Dosing for Patients With COVID-19: An Integrative Modeling Approach for Effective Drug Repurposing. Clin. Pharmacol. Ther. 108 (2), 253–263. 10.1002/cpt.1856 32285930 PMC7262072

[B33] Gatti M. De Ponti F. (2021). Drug Repurposing in the COVID-19 Era: Insights From Case Studies Showing Pharmaceutical Peculiarities. Pharmaceutics. 13 (3), 302. 10.3390/pharmaceutics13030302 33668969 PMC7996547

[B34] Gorbalenya A. E. Baker S. C. Baric R. S. de Groot R. J. Drosten C. Gulyaeva A. A. (2020). The Species Severe Acute Respiratory Syndrome-Related Coronavirus: Classifying 2019-nCoV and Naming it SARS-CoV-2. Nat. Microbiol. 5 536–544. 10.1038/s41564-020-0695-z 32123347 PMC7095448

[B35] Gordon D. E. Jang G. M. Bouhaddou M. Xu J. Obernier K. White K. M. (2020). A SARS-CoV-2 Protein Interaction Map Reveals Targets for Drug Repurposing. Nature. 583 (7816), 459–468. 10.1038/s41586-020-2286-9 32353859 PMC7431030

[B36] Greenwood J. R. Calkins D. Sullivan A. P. Shelley J. C. (2010). Towards the Comprehensive, Rapid, and Accurate Prediction of the Favorable Tautomeric States of Drug-Like Molecules in Aqueous Solution. J. Comput. Aided Mol. Des. 24 (6-7), 591–604. 10.1007/s10822-010-9349-1 20354892

[B37] Günther S. Reinke P. Y. Fernández-García Y. Lieske J. Lane T. J. Ginn H. M. (2021). X-ray Screening Identifies Active Site and Allosteric Inhibitors of SARS-CoV-2 Main Protease. Science 372 (6542), 642–646. 10.1126/science.abf7945 33811162 PMC8224385

[B38] Gyselinck I. Janssens W. Verhamme P. Vos R. (2021). Rationale for Azithromycin in COVID-19: an Overview of Existing Evidence. BMJ Open Resp Res. 8 (1), e000806. 10.1136/bmjresp-2020-000806 PMC7811960 33441373

[B39] Hanson Q. M. Wilson K. M. Shen M. Itkin Z. Eastman R. T. Shinn P. (2020). Targeting ACE2-RBD Interaction as a Platform for COVID-19 Therapeutics: Development and Drug-Repurposing Screen of an AlphaLISA Proximity Assay. ACS Pharmacol. Transl. Sci. 3 (6), 1352–1360. 10.1021/acsptsci.0c00161 33330843 PMC7688046

[B40] Hanwell M. D. Curtis D. E. Lonie D. C. Vandermeersch T. Zurek E. Hutchison G. R. (2012). Avogadro: An Advanced Semantic Chemical Editor, Visualization, and Analysis Platform. J. Cheminform. 4, 17. 10.1186/1758-2946-4-17 22889332 PMC3542060

[B41] Hilgenfeld R. Peiris M. (2013). From SARS to MERS: 10 Years of Research on Highly Pathogenic Human Coronaviruses. Antivir. Res. 100 (1), 286–295. 10.1016/j.antiviral.2013.08.015 24012996 PMC7113673

[B42] Irwin J. J. Duan D. Torosyan H. Doak A. K. Ziebart K. T. Sterling T. (2015). An Aggregation Advisor for Ligand Discovery. J. Med. Chem. 58 (17), 7076–7087. 10.1021/acs.jmedchem.5b01105 26295373 PMC4646424

[B43] Jin Z. Du X. Xu Y. Deng Y. Liu M. Zhao Y. (2020). Structure of Mpro from SARS-CoV-2 and Discovery of its Inhibitors. Nature. 582, 289–293. 10.1038/s41586-020-2223-y 32272481

[B44] Jukič M. Škrlj B. Tomšič G. Pleško S. Podlipnik Č. Bren U. (2021). Prioritisation of Compounds for 3CLpro Inhibitor Development on SARS-CoV-2 Variants. Molecules. 26 (10), 3003. 10.3390/molecules26103003 34070140 PMC8158358

[B45] Jukič M. Janežič D. Bren U. (2020). Ensemble Docking Coupled to Linear Interaction Energy Calculations for Identification of Coronavirus Main Protease (3CLpro) Non-Covalent Small-Molecule Inhibitors. Molecules. 25 (24), 5808. 10.3390/molecules25245808 PMC7763084 33316996

[B46] Kaddoura M. AlIbrahim M. Hijazi G. Soudani N. Audi A. Alkalamouni H. (2020). COVID-19 Therapeutic Options under Investigation. Front. Pharmacol. 11, 1196. 10.3389/fphar.2020.01196 32848795 PMC7424051

[B47] Kahn J. S. McIntosh K. (2005). History and Recent Advances in Coronavirus Discovery. Pediatr. Infect. Dis. J. 24 (11), S223–S227. 10.1097/01.inf.0000188166.17324.60 16378050

[B48] Kaur H. Shekhar N. Sharma S. Sarma P. Prakash A. Medhi B. (2021). Ivermectin as a Potential Drug for Treatment of COVID-19: An In-Sync Review With Clinical and Computational Attributes. Pharmacol. Rep. 73 (3), 736–749. 10.1007/s43440-020-00195-y 33389725 PMC7778723

[B49] Kern C. Schöning V. Chaccour C. Hammann F. (2021). Modeling of SARS-CoV-2 Treatment Effects for Informed Drug Repurposing. Front. Pharmacol. 12, 625678. 10.3389/fphar.2021.625678 33776767 PMC7988345

[B50] Khalili J. S. Zhu H. Mak N. S. A. Yan Y. Zhu Y. (2020). Novel Coronavirus Treatment With Ribavirin: Groundwork for an Evaluation Concerning COVID‐19. J. Med. Virol. 92 (7), 740–746. 10.1002/jmv.25798 32227493 PMC7228408

[B51] Kharkar P. S. Warrier S. Gaud R. S. (2014). Reverse Docking: a Powerful Tool for Drug Repositioning and Drug rescue. Future Med. Chem. 6 (3), 333–342. 10.4155/fmc.13.207 24575968

[B52] Konc J. Lešnik S. Škrlj B. Janežič D. (2021). ProBiS-Dock Database: A Web Server and Interactive Web Repository of Small Ligand−Protein Binding Sites for Drug Design. J. Chem. Inf. Model. 61 (8), 4097–4107. 10.1021/acs.jcim.1c00454 34319727

[B53] Kores K. Konc J. Bren U. (2021). Mechanistic Insights Into Side Effects of Troglitazone and Rosiglitazone Using a Novel Inverse Molecular Docking Protocol. Pharmaceutics. 13, 315. 10.3390/pharmaceutics13030315 33670968 PMC7997210

[B54] Kores K. Lešnik S. Bren U. Janežič D. Konc J. (2019). Discovery of Novel Potential Human Targets of Resveratrol by Inverse Molecular Docking. J. Chem. Inf. Model. 59, 2467–2478. 10.1021/acs.jcim.8b00981 30883115

[B55] Kumar Das J. Tradigo G. Veltri P. H Guzzi P. Roy S. (2021). Data Science in Unveiling COVID-19 Pathogenesis and Diagnosis: Evolutionary Origin to Drug Repurposing. Brief. Bioinform. 22 (2), 855–872. 10.1093/bib/bbaa420 33592108 PMC7929414

[B56] Kumar P. Bhardwaj T. Kumar A. Gehi B. R. Kapuganti S. K. Garg N. (2020). Reprofiling of Approved Drugs against SARS-CoV-2 Main Protease: An In-Silico Study. J. Biomol. Struct. Dyn., 1–15. 10.1080/07391102.2020.1845976 PMC7678354 33179586

[B57] Kuzikov M. Costanzi E. Reinshagen J. Esposito F. Vangeel L. Wolf M. (2021). Identification of Inhibitors of SARS-CoV-2 3CL-Pro Enzymatic Activity Using a Small Molecule *In Vitro* Repurposing Screen. ACS Pharmacol. Translational Sci. 4 (3), 1096–1110. 10.1021/acsptsci.0c00216 PMC7986981 35287429

[B58] Lai C.-C. Wang C.-Y. Hsueh P.-R. (2020). Co-Infections Among Patients With COVID-19: The Need for Combination Therapy With Non-Anti-SARS-CoV-2 Agents? J. Microbiol. Immunol. Infect. 53 (4), 505–512. 10.1016/j.jmii.2020.05.013 32482366 PMC7245213

[B59] Lamb Y. N. (2020). Remdesivir: First Approval. Drugs 80, 1355–1363. 10.1007/s40265-020-01378-w 32870481 PMC7459246

[B60] Li Q. Guan X. Wu P. Wang X. Zhou L. Tong Y. (2020). Early Transmission Dynamics in Wuhan, China, of Novel Coronavirus–Infected Pneumonia. New Engl. J. Med. 382, 1199–1207. 10.1056/NEJMoa2001316 31995857 PMC7121484

[B61] Lin C.-N. Chan K. R. Ooi E. E. Chiou M.-T. Hoang M. Hsueh P.-R. (2021). Animal Coronavirus Diseases: Parallels with COVID-19 in Humans. Viruses. 13 (8), 1507. 10.3390/v13081507 34452372 PMC8402828

[B62] Malik P. Patel U. Mehta D. Patel N. Kelkar R. Akrmah M. (2021). Biomarkers and Outcomes of COVID-19 Hospitalisations: Systematic Review and Meta-Analysis. Bmj Ebm. 26 (3), 107–108. 10.1136/bmjebm-2020-111536 PMC7493072 32934000

[B63] Meier K. Glatz T. Guijt M. C. Piccininni M. Van Der Meulen M. Atmar K. (2020). Public Perspectives on Protective Measures during the COVID-19 Pandemic in the Netherlands, Germany and Italy: A Survey Study. PloS one. 15 (8), e0236917. 10.1371/journal.pone.0236917 32756573 PMC7406072

[B64] Meo S. A. Klonoff D. C. Akram J. (2020). Efficacy of Chloroquine and Hydroxychloroquine in the Treatment of COVID-19. Eur. Rev. Med. Pharmacol. Sci. 24 (8), 4539–4547. 10.26355/eurrev_202004_21038 32373993

[B65] Meyer-Almes F.-J. (2020). Repurposing Approved Drugs as Potential Inhibitors of 3CL-Protease of SARS-CoV-2: Virtual Screening and Structure Based Drug Design. Comput. Biol. Chem. 88, 107351. 10.1016/j.compbiolchem.2020.107351 32769050 PMC7832737

[B66] Mody V. Ho J. Wills S. Mawri A. Lawson L. Ebert M. C. C. J. C. (2021). Identification of 3-chymotrypsin like Protease (3CLPro) Inhibitors as Potential Anti-SARS-CoV-2 Agents. Commun. Biol. 4 (1), 93–10. 10.1038/s42003-020-01577-x 33473151 PMC7817688

[B67] Moelling K. (2021). Within-Host and Between-Host Evolution in SARS-CoV-2-New Variant's Source. Viruses. 13 (5), 751. 10.3390/v13050751 33922936 PMC8146792

[B68] Morley S. D. Afshar M. (2004). Validation of an Empirical RNA-Ligand Scoring Function for Fast Flexible Docking Using RiboDock®. J. Comput. Aided Mol. Des. 18, 189–208. 10.1023/b:jcam.0000035199.48747.1e 15368919

[B69] Naqvi A. A. T. Fatima K. Mohammad T. Fatima U. Singh I. K. Singh A. (2020). Insights Into SARS-CoV-2 Genome, Structure, Evolution, Pathogenesis and Therapies: Structural Genomics Approach. Biochim. Biophys. Acta (Bba) - Mol. Basis Dis. 1866, 165878. 10.1016/j.bbadis.2020.165878 PMC7293463 32544429

[B70] Nardelli P. Zangrillo A. Sanchini G. Likhvantsev V. V. Yavorovskiy A. G. Garcia C. S. R. (2021). Crying Wolf in Time of Corona: The Strange Case of Ivermectin and Hydroxychloroquine. Is the Fear of Failure Withholding Potential Life-Saving Treatment from Clinical Use? Signa Vitae. 17 (3), 3–4. 10.22514/sv.2021.043

[B71] Nicola M. Alsafi Z. Sohrabi C. Kerwan A. Al-Jabir A. Iosifidis C. (2020). The Socio-Economic Implications of the Coronavirus Pandemic (COVID-19): A Review. Int. J. Surg. 78, 185–193. 10.1016/j.ijsu.2020.04.018 32305533 PMC7162753

[B72] O’Driscoll M. Dos Santos G. R. Wang L. Cummings D. A. Azman A. S. Paireau J. (2021). Age-specific Mortality and Immunity Patterns of SARS-CoV-2. Nature. 590 (7844), 140–145. 10.1038/s41586-020-2918-0 33137809

[B73] Osipiuk J. Azizi S. A. Dvorkin S. Endres M. Jedrzejczak R. Jones K. A. (2021). Structure of Papain-Like Protease From SARS-CoV-2 and its Complexes With Non-Covalent Inhibitors. Nat. Commun. 12 (1), 743–749. 10.1038/s41467-021-21060-3 33531496 PMC7854729

[B74] Pettersen E. F. Goddard T. D. Huang C. C. Couch G. S. Greenblatt D. M. Meng E. C. (2004). UCSF Chimera?A Visualization System for Exploratory Research and Analysis. J. Comput. Chem. 25, 1605–1612. 10.1002/jcc.20084 15264254

[B75] Pooladanda V. Thatikonda S. Godugu C. (2020). The Current Understanding and Potential Therapeutic Options to Combat COVID-19. Life Sci. 254, 117765. 10.1016/j.lfs.2020.117765 32437797 PMC7207108

[B76] Ribone S. P. Paz S. A. Abrams C. F. Villarreal M. A. (2021). Target Identification for Repurposed Drugs Active against SARS-CoV-2 via High-Throughput Inverse Docking. ChemRxiv. 25, 2021. 10.33774/chemrxiv-2021-2n6jh PMC8616721 34825285

[B77] Riva L. Yuan S. Yin X. Martin-Sancho L. Matsunaga N. Pache L. (2020). Discovery of SARS-CoV-2 Antiviral Drugs Through Large-Scale Compound Repurposing. Nature. 586 (7827), 113–119. 10.1038/s41586-020-2577-1 32707573 PMC7603405

[B78] Roy B. Dhillon J. Habib N. Pugazhandhi B. (2021). Global Variants of COVID-19: Current Understanding. J. Biomed. Sci. 8 (1), 8–11. 10.3126/jbs.v8i1.38453

[B79] Ruiz-Carmona S. Alvarez-Garcia D. Foloppe N. Garmendia-Doval A. B. Juhos S. Schmidtke P. (2014). rDock: A Fast, Versatile and Open Source Program for Docking Ligands to Proteins and Nucleic Acids. Plos Comput. Biol. 10 (4), e1003571. 10.1371/journal.pcbi.1003571 24722481 PMC3983074

[B80] Sadegh S. Matschinske J. Blumenthal D. B. Galindez G. Kacprowski T. List M. (2020). Exploring the SARS-CoV-2 Virus-Host-Drug Interactome for Drug Repurposing. Nat. Commun. 11 (1), 3518–3519. 10.1038/s41467-020-17189-2 32665542 PMC7360763

[B81] Saha R. P. Sharma A. R. Singh M. K. Samanta S. Bhakta S. Mandal S. (2020). Repurposing Drugs, Ongoing Vaccine, and New Therapeutic Development Initiatives against COVID-19. Front. Pharmacol. 11, 1258. 10.3389/fphar.2020.01258 32973505 PMC7466451

[B82] Sarkar C. Mondal M. Torequl Islam M. Martorell M. Docea A. O. Maroyi A. (2020). Potential Therapeutic Options for COVID-19: Current Status, Challenges, and Future Perspectives. Front. Pharmacol. 11, 572870. 10.3389/fphar.2020.572870 33041814 PMC7522523

[B83] Saubern S. Guha R. Baell J. B. (2011). KNIME Workflow to Assess PAINS Filters in SMARTS Format. Comparison of RDKit and Indigo Cheminformatics Libraries. Mol. Inf. 30 (10), 847–850. 10.1002/minf.201100076 27468104

[B84] Schmith V. D. Zhou J. Lohmer L. R. L. (2020). The Approved Dose of Ivermectin Alone Is Not the Ideal Dose for the Treatment of COVID‐19. Clin. Pharmacol. Ther. 108 (4), 762–765. 10.1002/cpt.1889 32378737 PMC7267287

[B85] Shah R. R. (2021). Chloroquine and Hydroxychloroquine for COVID‐19: Perspectives on Their Failure in Repurposing. J. Clin. Pharm. Ther. 46 (1), 17–27. 10.1111/jcpt.13267 32981089 PMC7537228

[B86] Shelley J. C. Cholleti A. Frye L. L. Greenwood J. R. Timlin M. R. Uchimaya M. (2007). Epik: a Software Program for pK a Prediction and Protonation State Generation for Drug-like Molecules. J. Comput. Aided Mol. Des. 21 (12), 681–691. 10.1007/s10822-007-9133-z 17899391

[B87] Shoichet B. K. (2006). Interpreting Steep Dose-Response Curves in Early Inhibitor Discovery. J. Med. Chem. 49 (25), 7274–7277. 10.1021/jm061103g 17149857

[B107] Singh T. U. Parida S. Lingaraju M. C. Kesavan M. Kumar D. Singh R. K. (2020). Drug Repurposing Approach to Fight COVID-19. Pharmacol. Rep. 72, 1479–1508. 10.1007/s43440-020-00155-6 32889701 PMC7474498

[B88] Sourimant J. Aggarwal M. Plemper R. K. (2021). Progress and Pitfalls of a Year of Drug Repurposing Screens against COVID-19. Curr. Opin. Virol. 49, 183–193. 10.1016/j.coviro.2021.06.004 34218010 PMC8214175

[B89] Štular T. Lešnik S. Rožman K. Schink J. Zdouc M. Ghysels A. (2016). Discovery of Mycobacterium Tuberculosis InhA Inhibitors by Binding Sites Comparison and Ligands Prediction. J. Med. Chem. 59, 11069–11078. 10.1021/acs.jmedchem.6b01277 27936766 PMC5588031

[B90] Tegally H. Wilkinson E. Giovanetti M. Iranzadeh A. Fonseca V. Giandhari J. (2020). Emergence and Rapid Spread of a New Severe Acute Respiratory Syndrome-Related Coronavirus 2 (SARS-CoV-2) Lineage with Multiple Spike Mutations in South Africa. MedRxiv 2020, 12.21.20248640. 10.1101/2020.12.21.20248640

[B91] Tummino T. A. Rezelj V. V. Fischer B. Fischer A. O’Meara M. J. Monel B. (2021). Drug-induced Phospholipidosis Confounds Drug Repurposing for SARS-CoV-2. Science 373(6554), 541–547. 10.1126/science.abi4708 34326236 PMC8501941

[B92] Verbeke R. Lentacker I. De Smedt S. C. Dewitte H. (2021). The Dawn of mRNA Vaccines: The COVID-19 Case. J. Controlled Release. 333, 511–520. 10.1016/j.jconrel.2021.03.043 PMC8008785 33798667

[B93] Volz E. Mishra S. Chand M. Barrett J. C. Johnson R. Geidelberg L. (2021). Transmission of SARS-CoV-2 Lineage B. 1.1. 7 in England: Insights From Linking Epidemiological and Genetic Data. MedRxiv. 2020, 2.30.20249034. 10.1101/2020.12.30.20249034

[B94] Walters W. P. Stahl M. T. Murcko M. A. (1998). Virtual Screening-An Overview. Drug Discov. Today. 3 (4), 160–178. 10.1016/s1359-6446(97)01163-x

[B95] Wang C. Horby P. W. Hayden F. G. Gao G. F. (2020). A Novel Coronavirus Outbreak of Global Health Concern. The lancet. 395 (10223), 470–473. 10.1016/s0140-6736(20)30185-9 PMC7135038 31986257

[B96] Wang F. Wu F.-X. Li C.-Z. Jia C.-Y. Su S.-W. Hao G.-F. (2019). ACID: a Free Tool for Drug Repurposing Using Consensus Inverse Docking Strategy. J. Cheminform. 11 (1), 73. 10.1186/s13321-019-0394-z 33430982 PMC6882193

[B97] Wei T.-z. Wang H. Wu X.-q. Lu Y. Guan S.-h. Dong F.-q. (2020). In Silico Screening of Potential Spike Glycoprotein Inhibitors of SARS-CoV-2 with Drug Repurposing Strategy. Chin. J. Integr. Med. 26 (9), 663–669. 10.1007/s11655-020-3427-6 32740825 PMC7395204

[B98] Wishart D. S. Feunang Y. D. Guo A. C. Lo E. J. Marcu A. Grant J. R. (2018). DrugBank 5.0: a Major Update to the DrugBank Database for 2018. Nucleic Acids Res. 46 (D1), D1074–D1082. 10.1093/nar/gkx1037 29126136 PMC5753335

[B99] Wu F. Zhao S. Yu B. Chen Y.-M. Wang W. Song Z.-G. (2020). A New Coronavirus Associated With Human Respiratory Disease in China. Nature. 579 (7798), 265–269. 10.1038/s41586-020-2008-3 32015508 PMC7094943

[B100] Yang Y.-L. Yu J.-Q. Huang Y.-W. (2020). Swine Enteric Alphacoronavirus (Swine Acute Diarrhea Syndrome Coronavirus): An Update Three Years after its Discovery. Virus. Res. 285, 198024. 10.1016/j.virusres.2020.198024 32482591 PMC7229464

[B108] Ye X.-T. Luo Y.-L Xia S.-C. Sun Q.-F. Ding J.-G. Zhou Y. (2010). Clinical Efficacy of Lopinavir/Ritonavir in the Treatment of Coronavirus Disease 2019. Eur. Rev. Med. Pharmacol. Sci. 24(6), 3390–3396. 10.26355/eurrev_202003_20706 32271456

[B101] Zaidi A. K. Dehgani-Mobaraki P. (2021). The Mechanisms of Action of Ivermectin Against SARS-CoV-2: An Evidence-Based Clinical Review Article. J. Antibiot., 1–13. 10.1038/s41429-021-00430-5 PMC8203399 34127807

[B102] Zhang L. Lin D. Sun X. Curth U. Drosten C. Sauerhering L. (2020). Crystal Structure of SARS-CoV-2 Main Protease Provides a Basis for Design of Improved α-ketoamide Inhibitors. Science. 368 (6489), 409–412. 10.1126/science.abb3405 32198291 PMC7164518

[B103] Zhou Q. Chen V. Shannon C. P. Wei X.-S. Xiang X. Wang X. (2020). Interferon-α2b Treatment for COVID-19. Front. Immunol. 11, 1061. 10.3389/fimmu.2020.01061 32574262 PMC7242746

[B104] Zhu N. Zhang D. Wang W. Li X. Yang B. Song J. (2020a). A Novel Coronavirus From Patients With Pneumonia in China, 2019. New Engl. J. Med. 382, 727–733. 10.1056/nejmoa2001017 31978945 PMC7092803

[B105] Zhu W. Xu M. Chen C. Z. Guo H. Shen M. Hu X. (2020b). Identification of SARS-CoV-2 3CL Protease Inhibitors by a Quantitative High-Throughput Screening. ACS Pharmacol. Transl. Sci. 3 (5), 1008–1016. 10.1021/acsptsci.0c00108 33062953 PMC7507806

[B106] Zhu T. Cao S. Su P.-C. Patel R. Shah D. Chokshi H. B. (2013). Hit Identification and Optimization in Virtual Screening: Practical Recommendations Based on a Critical Literature Analysis. J. Med. Chem. 56 (17), 6560–6572. 10.1021/jm301916b 23688234 PMC3772997

