# Supplementary material for: Repurposing of Drugs for SARS-CoV-2 Using Inverse Docking Fingerprints
Source: Front Chem. 2021 Dec 28;9:757826. doi: 10.3389/fchem.2021.757826 (PMC8748264; doi:10.3389/fchem.2021.757826)
Supplement: Supplementary file 1 [file DataSheet1.PDF]

Supplementary Information

Repurposing of Drugs for SARS-CoV-2 Using Inverse Docking Fingerprints

*Marko Jukič [1,2 #], Katarina Kores [1 #], Dušanka Janežič [2 *] and Urban Bren [1,2 *]*

[1] Laboratory of Physical Chemistry and Chemical Thermodynamics, Faculty of Chemistry and Chemical Engineering, University of Maribor, , Smetanova ulica 17, SI-2000 Maribor, Slovenia; marko.jukic@um.si

[2] Faculty of Mathematics, Natural Sciences and Information Technologies, University of Primorska, Glagoljaška 8, SI-6000 Koper, Slovenia

[#] *Authors contributed equally*

*** Correspondence:** Dušanka Janežič; Tel: + 386 5 611 76 59; email: dusanka.janezic@upr.si and Urban Bren; Tel: + 386 2 22 94 421; email: urban.bren@um.si.

Table S1. Combined 10 top-docking results for ivermectin B1a and B1b.

PDB ID with Chain	***Score***	***UniProt***	***Organism***	***Protein name***
2pk5A	-111.313	Q9J2P7	human immunodeficiency virus-1 HIV-1	Protease
5ah8A	-108.816	P03366	Human immunodeficiency virus type 1 (Z2/CDC-Z34 ISOLATE)	PROTEASE
1hxwB	-107.924	P12499	human immunodeficiency virus-1 HIV-1	HIV-1 PROTEASE
1iiqA	-106.019	P03367	human immunodeficiency virus-1 HIV-1	PROTEASE RETROPEPSIN
2avmA	-105.357	P04587	human immunodeficiency virus-1 HIV-1	HIV-1 protease
1a9mB	-104.804	P03366	human immunodeficiency virus-1 HIV-1	HIV-1 PROTEASE
3gi6A	-104.269	P03369	human immunodeficiency virus-1 HIV-1	Protease
6dv0B	-104.208	P04585	human immunodeficiency virus-1 HIV-1	Protease
2q55A	-103.635	O38719	human immunodeficiency virus-1 HIV-1	Protease
5cokB	-103.397	G0X8E8	human immunodeficiency virus-1 HIV-1	HIV-1 protease

Table S2. 10 top-docking results for selamectin.

PDB ID with Chain	***Score***	***UniProt***	***Organism***	***Protein name***
5lc0A	-89.903	A0A140DLX4	ZIKV	NS2B-NS3 protease,NS2B-NS3 protease
1hxwB	-86.8758	P12499	human immunodeficiency virus-1 HIV-1	HIV-1 PROTEASE
1bv7B	-85.6238	P04585	human immunodeficiency virus-1 HIV-1	PROTEIN (HIV-1 PROTEASE)
1hviA	-85.1895	O92139	human immunodeficiency virus-1 HIV-1	HIV-1 PROTEASE
1ivqB	-83.8065	P04584	human immunodeficiency virus type 2, HIV-2	HIV-2 PROTEASE
4djqA	-83.7874	Q90K99	human immunodeficiency virus-1 HIV-1	Pol polyprotein
6bz2B	-83.5656	P03366	human immunodeficiency virus-1 HIV-1	Protease
3lzsB	-83.3717	P24740	human immunodeficiency virus-1 HIV-1	HIV-1 protease
3vf7A	-83.2471	P03367	Human immunodeficiency virus type 1 (BRU ISOLATE)	protease
1wbkA	-82.2659	Q8Q3H0	human immunodeficiency virus-1 HIV-1	POL PROTEIN (FRAGMENT)

Table S3. Combined 10 top-docking results for chloroquine and hydroxychloroquine.

PDB ID with Chain	***Score***	***UniProt***	***Organism***	***Protein name***
6snwA	-75.4441	Q6JKR9	Human enterovirus CVA10	Capsid protein VP1
7bznA	-70.6169	A0A0C5AWF6	Human enterovirus CVA10	Capsid protein VP1
6lhbA	-70.4093	Q9QF31	Human enterovirus CVA16	VP1
4fzbC	-69.6592	O41156	Paramecium bursaria Chlorella virus PBCV-1	Probable thymidylate synthase
1iiqB	-69.3791	P03367	human immunodeficiency virus-1 HIV-1	PROTEASE RETROPEPSIN
4adjA	-69.2024	P08563	Rubella virus	E1 ENVELOPE GLYCOPROTEIN
6b3hB	-67.7853	P04587	human immunodeficiency virus-1 HIV-1	HIV-1 Protease
2i4xB	-67.6191	Q903J0	human immunodeficiency virus-1 HIV-1	Protease
6o57A	-67.5429	I7BFC3	human immunodeficiency virus-1 HIV-1	HIV-1 protease
3ngbG	-67.2868	Q0ED31	human immunodeficiency virus-1 HIV-1	Envelope glycoprotein gp160

Table S4. List of Coronaviridae family targets, to which amikacin successfully docked.

PDB ID with Chain	***Score***	***UniProt***	***Organism***	***Protein name***
5gwyA	-93.3008	P0C6U6	Human coronavirus NL	main protease
6xqsA	-87.6338	P0DTD1	Wuhan coronavirus	3C-like proteinase
1wofB	-86.5213	P0C6X7	Severe acute respiratory syndrome coronavirus	3C-like proteinase
4wmeB	-86.4021	W6A941	Middle East respiratory syndrome coronavirus	MERS-CoV 3CL protease
6xhoB	-84.1304	P0C6U8	Severe acute respiratory syndrome coronavirus	3C-like proteinase
4yo9B	-84.0367	P0C6W3	Bat coronavirus HKU4	3C-like proteinase
5ynnA	-83.6106	K0BWD0	human betacoronavirus 2c EMC	nsp16 protein
4zroA	-79.8456	Q98VG9	Feline infectious peritonitis virus (strain 79-1146)	3C-like proteinase
2ynbB	-78.2081	P0C6T4	Bat coronavirus HKU4	3C-LIKE PROTEINASE
6l70B	-76.5304	K4L9I6	porcine epidemic diarrhoea virus	PEDV main protease
2q6dB	-76.3977	P0C6Y3	infectious bronchitis virus IBV	Infectious bronchitis virus (IBV) main protease
2zu2B	-76.3558	P0C6U2	Human coronavirus strain 229E	3C-like proteinase
6jijB	-75.9427	P0C6X9	Murine hepatitis virus (strain A59)	Replicative polyprotein 1ab
5wklA	-75.8867	K9N638	Middle East respiratory syndrome coronavirus	Orf1a protein
5gwzB	-75.8171	P0C6V6	Porcine epidemic diarrhea virus (strain CV777)	PEDV main protease
6ohwB	-75.6671	Q696P8	Human coronavirus strain OC43	Spike surface glycoprotein
4yluA	-75.6249	V9TU12	Middle East respiratory syndrome coronavirus	ORF1a protein
3d23C	-75.436	P0C6U3	Human coronavirus HKU1 (isolate N1)	3C-like proteinase
5c3nB	-75.406	V9TU05	Middle East respiratory syndrome coronavirus	ORF1a protein
5hyoB	-75.3655	U6BPB2	porcine epidemic diarrhoea virus	PEDV 3CLpro
2ampB	-74.4825	P0C6Y5	transmissible gastroenteritis virus TGEV	3C-like proteinase
2q6fB	-73.2794	P0C6V5	infectious bronchitis virus IBV	Infectious bronchitis virus (IBV) main protease
5nh0C	-72.7199	P0C6X5	Human coronavirus NL	3C-like proteinase
5zqgB	-71.0044	R4JK63	porcine epidemic diarrhoea virus	Non-structural protein
6u7kB	-70.3975	Q91AV1	Porcine epidemic diarrhea virus (strain CV777)	Spike glycoprotein
4f49A	-68.9848	P0C6V2	Porcine transmissible gastroenteritis coronavirus (STRAIN PURDUE)	3C-like proteinase
5jilA	-67.1155	Q3HS77	Rat coronavirus	Hemagglutinin-esterase
4lmtA	-66.2212	P33469	Human coronavirus strain OC43	Nucleoprotein
6nozA	-65.0647	W8QLX4	porcine epidemic diarrhoea virus	Polyprotein
4c7wB	-63.5141	P31614	murine hepatitis virus, MHV	HEMAGGLUTININ-ESTERASE
5nfyA	-60.2988	Q1T6X8	SARS coronavirus Frankfurt 1	Polyprotein 1ab
4pt5A	-59.4445	K4LC41	human betacoronavirus 2c EMC	Papain-like protease
5jifB	-58.4478	O92367	Murine coronavirus (strain DVIM)	Hemagglutinin-esterase
5yvdA	-58.4248	A0A0U2GPI9	Middle East respiratory syndrome coronavirus	Nsp15
3cl5A	-57.3933	P15776	neonatal calf diarrhea virus	Hemagglutinin-esterase
6m3mA	-56.7036	P0DTC9	Wuhan coronavirus	Nucleoprotein

6kl2C	-55.6832	K9N4V7	Middle East respiratory syndrome coronavirus	Nucleoprotein
1sskA	-53.1345	P59595	Severe acute respiratory syndrome coronavirus	Nucleocapsid protein
4rezA	-51.841	M4STU1	Human betacoronavirus 2c strain Jordan-N3/2012	ORF1ab protein
2gecB	-51.6166	P32923	infectious bronchitis virus IBV	Nucleocapsid protein
6q04C	-47.9983	K0BRG7	human betacoronavirus 2c EMC	Spike glycoprotein
5n11A	-47.0157	Q4VID6	Human coronavirus strain OC43	Hemagglutinin-esterase
6l8qB	-45.7336	K9N5Q8	Middle East respiratory syndrome coronavirus	Spike glycoprotein
6l5tA	-45.6625	A0A385H8D7	Swine acute diarrhea syndrome coronavirus	Peptidase C16
6vybA	-43.8942	P0DTC2	Wuhan coronavirus	Spike glycoprotein
6m15A	-43.1245	A8JNZ2	Bat coronavirus HKU2	Spike glycoprotein
6u7hB	-39.7457	P15423	Human coronavirus strain 229E	spike glycoprotein
6y3yA	-35.5868	Q5MQD1	Human CoV/HKU1	Hemagglutinin-esterase
5x58A	-25.5834	P59594	SARS coronavirus BJ01	Spike glycoprotein

Table S5. List of Coronaviridae family targets, to which canagliflozin successfully docked.

PDB ID with Chain	***Score***	***UniProt***	***Organism***	***Protein name***
5gwzB	-54.8918	P0C6V6	Porcine epidemic diarrhea virus (strain CV777)	PEDV main protease
2h2zA	-52.5384	P0C6X7	Severe acute respiratory syndrome coronavirus	Replicase polyprotein 1ab
5gwyA	-51.9845	P0C6U6	Human coronavirus NL	main protease
6xbhA	-49.7296	P0DTD1	Wuhan coronavirus	3C-like proteinase
3mj5A	-49.0391	P0C6U8	Severe acute respiratory syndrome coronavirus	Replicase polyprotein 1a
4wmeB	-48.5037	W6A941	Middle East respiratory syndrome coronavirus	MERS-CoV 3CL protease
2ynbB	-48.0252	P0C6T4	Bat coronavirus HKU4	3C-LIKE PROTEINASE
5c3nA	-45.9224	V9TU05	Middle East respiratory syndrome coronavirus	ORF1a protein
6jijA	-45.4373	P0C6X9	Murine hepatitis virus (strain A59)	Replicative polyprotein 1ab
4yo9A	-44.8257	P0C6W3	Bat coronavirus HKU4	3C-like proteinase
4xfqB	-44.5828	K4L9I6	porcine epidemic diarrhoea virus	PEDV main protease
2zu2B	-42.5297	P0C6U2	Human coronavirus strain 229E	3C-like proteinase
2ampB	-42.4682	P0C6Y5	transmissible gastroenteritis virus TGEV	3C-like proteinase
4lm9A	-41.858	P33469	Human coronavirus strain OC43	Nucleoprotein
6fv1A	-41.5605	P0C6X5	Human coronavirus NL	3C-like proteinase
4c7wB	-41.2309	P31614	murine hepatitis virus, MHV	HEMAGGLUTININ-ESTERASE
2q6dA	-41.1471	P0C6Y3	infectious bronchitis virus IBV	Infectious bronchitis virus (IBV) main protease
4zuhA	-41.1471	R4JK63	porcine epidemic diarrhoea virus	PEDV 3C-Like protease
5ynjA	-41.0821	K0BWD0	human betacoronavirus 2c EMC	nsp16 protein
4yluA	-39.8587	V9TU12	Middle East respiratory syndrome coronavirus	ORF1a protein
5hyoA	-39.0922	U6BPB2	porcine epidemic diarrhoea virus	PEDV 3CLpro
2q6fB	-38.6493	P0C6V5	infectious bronchitis virus IBV	Infectious bronchitis virus (IBV) main protease

3d23A	-37.9615	P0C6U3	Human coronavirus HKU1 (isolate N1)	3C-like proteinase
4rezA	-37.522	M4STU1	Human betacoronavirus 2c strain Jordan-N3/2012	ORF1ab protein
6kl2A	-37.0705	K9N4V7	Middle East respiratory syndrome coronavirus	Nucleoprotein
5wkjA	-36.861	K9N638	Middle East respiratory syndrome coronavirus	Orf1a protein
6l5tA	-36.0813	A0A385H8D7	Swine acute diarrhea syndrome coronavirus	Peptidase C16
4zroA	-35.9708	Q98VG9	Feline infectious peritonitis virus (strain 79-1146)	3C-like proteinase
5yvdA	-35.7164	A0A0U2GPI9	Middle East respiratory syndrome coronavirus	Nsp15
6nzkB	-34.7194	Q696P8	Human coronavirus strain OC43	Spike surface glycoprotein
5w9oJ	-34.6568	K9N5Q8	Middle East respiratory syndrome coronavirus	Spike glycoprotein
5nfyA	-34.5373	Q1T6X8	SARS coronavirus Frankfurt 1	Polyprotein 1ab
6nozA	-34.0719	W8QLX4	porcine epidemic diarrhoea virus	Polyprotein
6wkpC	-33.7399	P0DTC9	Wuhan coronavirus	Nucleoprotein
4wurA	-33.1557	K9N7C7	Betacoronavirus England 1	Papain-like protease
6nb3A	-33.1296	A0A140AYW5	Middle East respiratory syndrome coronavirus	Spike glycoprotein
6q06A	-32.9471	K0BRG7	human betacoronavirus 2c EMC	Spike glycoprotein
6vxxB	-32.5735	P0DTC2	Wuhan coronavirus	Spike glycoprotein
6y3yA	-32.5314	Q5MQD1	Human CoV/HKU1	Hemagglutinin-esterase
2c86A	-32.0335	P69598	infectious bronchitis virus IBV	NUCLEOCAPSID PROTEIN
4f49A	-31.9574	P0C6V2	Porcine transmissible gastroenteritis coronavirus (STRAIN PURDUE)	3C-like proteinase
6accA	-31.8078	P59594	Severe acute respiratory syndrome coronavirus	Spike glycoprotein
5jilA	-31.7626	Q3HS77	Rat coronavirus	Hemagglutinin-esterase
3cl5A	-31.3432	P15776	neonatal calf diarrhea virus	Hemagglutinin-esterase
6u7hB	-31.2535	P15423	Human coronavirus strain 229E	spike glycoprotein
4pt5A	-30.7586	K4LC41	human betacoronavirus 2c EMC	Papain-like protease
2gecB	-30.677	P32923	infectious bronchitis virus IBV	Nucleocapsid protein
2ofzA	-30.66	P59595	Severe acute respiratory syndrome-related coronavirus Tor2	Nucleocapsid protein
6u7kB	-29.9265	Q91AV1	Porcine epidemic diarrhea virus (strain CV777)	Spike glycoprotein
5n11A	-29.661	Q4VID6	Human coronavirus strain OC43	Hemagglutinin-esterase
3jclC	-29.4339	P11224	Murine hepatitis virus (strain A59)	Spike glycoprotein
5i08C	-29.4024	Q0ZME7	Human coronavirus HKU1 (isolate N5)	Spike glycoprotein,Foldon chimera
6qfyA	-28.6309	A0A1Z2WUW0	Porcine hemagglutinating encephalomyelitis coronavirus	Spike glycoprotein
6m15C	-27.5286	A8JNZ2	Bat coronavirus HKU2	Spike glycoprotein
4h14A	-27.3866	Q1HLC5	neonatal calf diarrhea virus	Spike glycoprotein
4l3nA	-27.1706	M4SVE7	Human betacoronavirus 2c strain Jordan-N3/2012	S protein
6m16B	-25.1062	A0A2P1G1L3	Swine acute diarrhea syndrome coronavirus	Spike glycoprotein
5jifA	-24.302	O92367	Murine coronavirus (strain DVIM)	Hemagglutinin-esterase
6b7nB	-24.2792	A0A075E3D7	Deltacoronavirus SDCV/USA/Ohio137/2014	Spike protein
6zgfC	-14.8464	A0A6B9WHD3	Bat coronavirus isolate RaTG13	Spike glycoprotein

Table S6. List of Coronaviridae family targets, to which cariprazine successfully docked.

PDB ID with Chain	*Score*	*UniProt*	*Organism*	*Protein name*
3atwB	-69.2402	P0C6U8	Severe acute respiratory syndrome coronavirus	3C-Like Proteinase
5gwzB	-63.7157	P0C6V6	Porcine epidemic diarrhea virus (strain CV777)	PEDV main protease
4yo9A	-63.0843	P0C6W3	Bat coronavirus HKU4	3C-like proteinase
6xa4A	-62.114	P0DTD1	Wuhan coronavirus	3C-like proteinase
4wmeB	-61.4553	W6A941	Middle East respiratory syndrome coronavirus	MERS-CoV 3CL protease
5ynoA	-60.3035	K0BWD0	human betacoronavirus 2c EMC	nsp16 protein
5gwyA	-60.0945	P0C6U6	Human coronavirus NL	main protease
2ynbA	-59.5699	P0C6T4	Bat coronavirus HKU4	3C-LIKE PROTEINASE
2bx4A	-58.7312	P0C6X7	SARS coronavirus Sin2774	3C-LIKE PROTEINASE
5c3nA	-57.2069	V9TU05	Middle East respiratory syndrome coronavirus	ORF1a protein
6jijB	-57.0098	P0C6X9	Murine hepatitis virus (strain A59)	Replicative polyprotein 1ab
4zroD	-56.8665	Q98VG9	Feline infectious peritonitis virus (strain 79-1146)	3C-like proteinase
2q6fB	-55.9024	P0C6V5	infectious bronchitis virus IBV	Infectious bronchitis virus (IBV) main protease
4wurA	-54.3333	K9N7C7	Betacoronavirus England 1	Papain-like protease
4xfqA	-53.7722	K4L9I6	porcine epidemic diarrhoea virus	PEDV main protease
3d23B	-53.2591	P0C6U3	Human coronavirus HKU1 (isolate N1)	3C-like proteinase
6fv1A	-51.7755	P0C6X5	Human coronavirus NL	3C-like proteinase
1p9sA	-50.9396	P0C6U2	Human coronavirus strain 229E	Replicase polyprotein 1ab
2q6dB	-49.7657	P0C6Y3	infectious bronchitis virus IBV	Infectious bronchitis virus (IBV) main protease
5wkmA	-49.096	K9N638	Middle East respiratory syndrome coronavirus	Orf1a protein
4f49A	-48.6483	P0C6V2	Porcine transmissible gastroenteritis coronavirus (STRAIN PURDUE)	3C-like proteinase
4c7wB	-47.7756	P31614	murine hepatitis virus, MHV	HEMAGGLUTININ-ESTERASE
2ampB	-47.6803	P0C6Y5	transmissible gastroenteritis virus TGEV	3C-like proteinase
4zuhB	-47.5744	R4JK63	porcine epidemic diarrhoea virus	PEDV 3C-Like protease
5jilA	-47.3238	Q3HS77	Rat coronavirus	Hemagglutinin-esterase
5hyoB	-46.46	U6BPB2	porcine epidemic diarrhoea virus	PEDV 3CLpro
6l5tA	-46.1962	A0A385H8D7	Swine acute diarrhea syndrome coronavirus	Peptidase C16
6wkpC	-45.976	P0DTC9	Wuhan coronavirus	Nucleoprotein
4rspA	-45.8813	V9TU12	Middle East respiratory syndrome coronavirus	Orf1a protein
6ohwB	-45.4358	Q696P8	Human coronavirus strain OC43	Spike surface glycoprotein
6kl6A	-44.1512	K9N4V7	Middle East respiratory syndrome coronavirus	Nucleoprotein
6nozA	-43.2027	W8QLX4	porcine epidemic diarrhoea virus	Polyprotein
1sskA	-42.8416	P59595	Severe acute respiratory syndrome coronavirus	Nucleocapsid protein
6u7kB	-42.1573	Q91AV1	Porcine epidemic diarrhea virus (strain CV777)	Spike glycoprotein
4pt5A	-41.7125	K4LC41	human betacoronavirus 2c EMC	Papain-like protease

5yvdA	-40.189	A0A0U2GPI9	Middle East respiratory syndrome coronavirus	Nsp15
5nfyA	-38.5602	Q1T6X8	SARS coronavirus Frankfurt 1	Polyprotein 1ab
5w9nD	-37.9892	K9N5Q8	Middle East respiratory syndrome coronavirus	MERS S
3cl5A	-37.6965	P15776	neonatal calf diarrhea virus	Hemagglutinin-esterase
4lmtA	-36.0935	P33469	Human coronavirus strain OC43	Nucleoprotein
2c86A	-35.718	P69598	infectious bronchitis virus IBV	NUCLEOCAPSID PROTEIN
2gecB	-34.6507	P32923	infectious bronchitis virus IBV	Nucleocapsid protein
7c2lB	-34.5766	P0DTC2	Wuhan coronavirus	Spike glycoprotein
6zgfC	-33.7673	A0A6B9WHD3	Bat coronavirus isolate RaTG13	Spike glycoprotein
5n11B	-33.2187	Q4VID6	Human coronavirus strain OC43	Hemagglutinin-esterase
4rezA	-31.637	M4STU1	Human betacoronavirus 2c strain Jordan-N3/2012	ORF1ab protein
6vsjA	-31.0628	P11224	Murine hepatitis virus (strain A59)	Spike glycoprotein
6u7hB	-30.5844	P15423	Human coronavirus strain 229E	spike glycoprotein
5jifA	-22.7543	O92367	Murine coronavirus (strain DVIM)	Hemagglutinin-esterase
6y3yA	-21.2763	Q5MQD1	Human CoV/HKU1	Hemagglutinin-esterase
5do2A	-20.846	K0BRG7	Middle East respiratory syndrome coronavirus	S protein
6qfyA	-19.9426	A0A1Z2WUW0	Porcine hemagglutinating encephalomyelitis coronavirus	Spike glycoprotein
6m16C	-19.3776	A0A2P1G1L3	Swine acute diarrhea syndrome coronavirus	Spike glycoprotein
6acgA	-13.9137	P59594	Severe acute respiratory syndrome coronavirus	Spike glycoprotein

Table S7. List of Coronaviridae family targets, to which chloroquine successfully docked.

PDB ID with Chain	*Score*	*UniProt*	*Organism*	*Protein name*
4zroC	-57.2338	Q98VG9	Feline infectious peritonitis virus (strain 79-1146)	3C-like proteinase
6xhlA	-55.9668	P0C6U8	Severe acute respiratory syndrome coronavirus	3C-like proteinase
5gwzA	-53.6394	P0C6V6	Porcine epidemic diarrhea virus (strain CV777)	PEDV main protease
4wmeA	-52.3921	W6A941	Middle East respiratory syndrome coronavirus	MERS-CoV 3CL protease
7bw4A	-52.0321	P0DTD1	Wuhan coronavirus	RNA-directed RNA polymerase
3d23D	-50.742	P0C6U3	Human coronavirus HKU1 (isolate N1)	3C-like proteinase
2a5kA	-50.6558	P0C6X7	Severe acute respiratory syndrome coronavirus	3C-like peptidase
2ynbA	-50.6223	P0C6T4	Bat coronavirus HKU4	3C-LIKE PROTEINASE
4yoiA	-48.5522	P0C6W3	Bat coronavirus HKU4	3C-like proteinase
2q6dB	-47.8228	P0C6Y3	infectious bronchitis virus IBV	Infectious bronchitis virus (IBV) main protease
2q6fB	-47.3439	P0C6V5	infectious bronchitis virus IBV	Infectious bronchitis virus (IBV) main protease
4pt5A	-47.1783	K4LC41	human betacoronavirus 2c EMC	Papain-like protease
3tloB	-47.1416	P0C6U6	Human coronavirus NL	3C-like proteinase
2ampB	-45.5922	P0C6Y5	transmissible gastroenteritis virus TGEV	3C-like proteinase
5wkjA	-45.2327	K9N638	Middle East respiratory syndrome coronavirus	Orf1a protein

5c3nA	-44.4014	V9TU05	Middle East respiratory syndrome coronavirus	ORF1a protein
5x5bB	-42.6861	P59594	SARS coronavirus BJ01	Spike glycoprotein
4xfqA	-42.5882	K4L9I6	porcine epidemic diarrhoea virus	PEDV main protease
6jijA	-42.5041	P0C6X9	Murine hepatitis virus (strain A59)	Replicative polyprotein 1ab
6nzkB	-42.3724	Q696P8	Human coronavirus strain OC43	Spike surface glycoprotein
5nfyA	-42.2522	Q1T6X8	SARS coronavirus Frankfurt 1	Polyprotein 1ab
4p16A	-41.894	K0BWD0	human betacoronavirus 2c EMC	ORF1a
6vxxC	-41.7442	P0DTC2	Wuhan coronavirus	Spike glycoprotein
5nh0B	-41.2759	P0C6X5	Human coronavirus NL	3C-like proteinase
2zu2A	-40.6282	P0C6U2	Human coronavirus strain 229E	3C-like proteinase
5yvdA	-40.0939	A0A0U2GPI9	Middle East respiratory syndrome coronavirus	Nsp15
6l8qF	-39.6999	K9N5Q8	Middle East respiratory syndrome coronavirus	Spike glycoprotein
6vsjC	-39.5872	P11224	Murine hepatitis virus (strain A59)	Spike glycoprotein
5jilA	-38.4588	Q3HS77	Rat coronavirus	Hemagglutinin-esterase
4f49A	-37.8741	P0C6V2	Porcine transmissible gastroenteritis coronavirus (STRAIN PURDUE)	3C-like proteinase
4c7lB	-37.8379	P31614	murine hepatitis virus MHV-S	HEMAGGLUTININ-ESTERASE
4yluB	-37.4958	V9TU12	Middle East respiratory syndrome coronavirus	ORF1a protein
6yi3A	-37.1728	P0DTC9	Wuhan coronavirus	Nucleoprotein
6kl5C	-36.9217	K9N4V7	Middle East respiratory syndrome coronavirus	Nucleoprotein
6u7kB	-36.7136	Q91AV1	Porcine epidemic diarrhea virus (strain CV777)	Spike glycoprotein
2gecB	-36.608	P32923	infectious bronchitis virus IBV	Nucleocapsid protein
5zqgB	-36.4876	R4JK63	porcine epidemic diarrhoea virus	Non-structural protein
6b7nB	-36.4423	A0A075E3D7	Deltacoronavirus SDCV/USA/Ohio137/2014	Spike protein
5hyoA	-35.5434	U6BPB2	porcine epidemic diarrhoea virus	PEDV 3CLpro
6m15B	-34.9517	A8JNZ2	Bat coronavirus HKU2	Spike glycoprotein
3cl5A	-34.2675	P15776	neonatal calf diarrhea virus	Hemagglutinin-esterase
6nb4A	-34.189	A0A140AYW5	Middle East respiratory syndrome coronavirus	Spike glycoprotein
4wurA	-34.1089	K9N7C7	Betacoronavirus England 1	Papain-like protease
4lm9A	-33.9162	P33469	Human coronavirus strain OC43	Nucleoprotein
6nozA	-33.8385	W8QLX4	porcine epidemic diarrhoea virus	Polyprotein
5jifA	-32.6114	O92367	Murine coronavirus (strain DVIM)	Hemagglutinin-esterase
4l3nA	-32.4586	M4SVE7	Human betacoronavirus 2c strain Jordan-N3/2012	S protein
6y3yB	-32.4163	Q5MQD1	Human CoV/HKU1	Hemagglutinin-esterase
6q04C	-32.3788	K0BRG7	human betacoronavirus 2c EMC	Spike glycoprotein
6u7hB	-31.8518	P15423	Human coronavirus strain 229E	spike glycoprotein
4rf1A	-31.7112	M4STU1	Human betacoronavirus 2c strain Jordan-N3/2012	ORF1ab protein
5i08C	-31.2658	Q0ZME7	Human coronavirus HKU1 (isolate N5)	Spike glycoprotein,Foldon chimera
6zgfC	-29.535	A0A6B9WHD3	Bat coronavirus isolate RaTG13	Spike glycoprotein
2ofzA	-27.9807	P59595	Severe acute respiratory syndrome-related coronavirus Tor2	Nucleocapsid protein

2c86A	-27.4979	P69598	infectious bronchitis virus IBV	NUCLEOCAPSID PROTEIN
6jx7B	-27.0071	C6GHB7	FIPV	Feline Infectious Peritonitis Virus Spike Protein
6vv5B	-25.238	S5RJN4	porcine epidemic diarrhoea virus	Spike glycoprotein
6m16B	-24.7045	A0A2P1G1L3	Swine acute diarrhea syndrome coronavirus	Spike glycoprotein
6l5tA	-24.4112	A0A385H8D7	Swine acute diarrhea syndrome coronavirus	Peptidase C16
6bfuB	-19.7075	A0A140ESF1	Porcine deltacoronavirus	Spike protein
5n11B	-18.0129	Q4VID6	Human coronavirus strain OC43	Hemagglutinin-esterase
4h14A	-16.0557	Q1HLC5	neonatal calf diarrhea virus	Spike glycoprotein
6qfyA	-13.4628	A0A1Z2WUW0	Porcine hemagglutinating encephalomyelitis coronavirus	Spike glycoprotein
6g13C	-11.8608	A0A0D3MU51	Middle East respiratory syndrome coronavirus	Nucleoprotein

Table S8. List of Coronaviridae family targets, to which darolutamide successfully docked.

PDB ID with Chain	***Score***	***UniProt***	***Organism***	***Protein name***
2ynaB	-62.0017	P0C6T4	Bat coronavirus HKU4	3C-LIKE PROTEINASE
5r84A	-60.9431	P0DTD1	Wuhan coronavirus	3C-like proteinase
5nh0B	-60.8713	P0C6X5	Human coronavirus NL	3C-like proteinase
3m3sB	-60.8126	P0C6X7	Severe acute respiratory syndrome coronavirus	3C-like proteinase
4yo9A	-60.6143	P0C6W3	Bat coronavirus HKU4	3C-like proteinase
4wmeB	-59.6287	W6A941	Middle East respiratory syndrome coronavirus	MERS-CoV 3CL protease
3d23D	-57.8682	P0C6U3	Human coronavirus HKU1 (isolate N1)	3C-like proteinase
5ynpA	-57.6368	K0BWD0	human betacoronavirus 2c EMC	nsp16 protein
3aw0A	-56.1037	P0C6U8	Severe acute respiratory syndrome coronavirus	3C-Like Proteinase
4wurA	-53.6255	K9N7C7	Betacoronavirus England 1	Papain-like protease
5gwyB	-53.6056	P0C6U6	Human coronavirus NL	main protease
5gwzB	-53.4794	P0C6V6	Porcine epidemic diarrhea virus (strain CV777)	PEDV main protease
6jijB	-51.1895	P0C6X9	Murine hepatitis virus (strain A59)	Replicative polyprotein 1ab
6wkpC	-50.1159	P0DTC9	Wuhan coronavirus	Nucleoprotein
5wkkA	-49.7233	K9N638	Middle East respiratory syndrome coronavirus	Orf1a protein
2q6fB	-49.6304	P0C6V5	infectious bronchitis virus IBV	Infectious bronchitis virus (IBV) main protease
6nzkB	-49.2049	Q696P8	Human coronavirus strain OC43	Spike surface glycoprotein
4yluB	-48.5906	V9TU12	Middle East respiratory syndrome coronavirus	ORF1a protein
5zqgB	-48.3525	R4JK63	porcine epidemic diarrhoea virus	Non-structural protein
2q6dA	-48.3369	P0C6Y3	infectious bronchitis virus IBV	Infectious bronchitis virus (IBV) main protease
4lmtA	-47.6327	P33469	Human coronavirus strain OC43	Nucleoprotein
4zroC	-47.0753	Q98VG9	Feline infectious peritonitis virus (strain 79-1146)	3C-like proteinase
2ampA	-46.9996	P0C6Y5	transmissible gastroenteritis virus TGEV	3C-like proteinase
6kl6C	-46.5553	K9N4V7	Middle East respiratory syndrome coronavirus	Nucleoprotein
6nozA	-46.3295	W8QLX4	porcine epidemic diarrhoea virus	Polyprotein

4pt5A	-45.8053	K4LC41	human betacoronavirus 2c EMC	Papain-like protease
2zu2A	-45.3292	P0C6U2	Human coronavirus strain 229E	3C-like proteinase
4xfqB	-44.4583	K4L9I6	porcine epidemic diarrhoea virus	PEDV main protease
4f49A	-44.4363	P0C6V2	Porcine transmissible gastroenteritis coronavirus (STRAIN PURDUE)	3C-like proteinase
6u7kB	-44.4044	Q91AV1	Porcine epidemic diarrhea virus (strain CV777)	Spike glycoprotein
5yvdB	-42.0617	A0A0U2GPI9	Middle East respiratory syndrome coronavirus	Nsp15
5w9mA	-41.7993	K9N5Q8	Middle East respiratory syndrome coronavirus	Spike glycoprotein
5c3nB	-41.7886	V9TU05	Middle East respiratory syndrome coronavirus	ORF1a protein
6q04C	-41.6988	K0BRG7	human betacoronavirus 2c EMC	Spike glycoprotein
2gecB	-41.6335	P32923	infectious bronchitis virus IBV	Nucleocapsid protein
5nfyA	-41.3368	Q1T6X8	SARS coronavirus Frankfurt 1	Polyprotein 1ab
5hyoA	-41.044	U6BPB2	porcine epidemic diarrhoea virus	PEDV 3CLpro
4c7lB	-40.6384	P31614	murine hepatitis virus MHV-S	HEMAGGLUTININ-ESTERASE
3cl5A	-40.3408	P15776	neonatal calf diarrhea virus	Hemagglutinin-esterase
4rf1A	-39.4932	M4STU1	Human betacoronavirus 2c strain Jordan-N3/2012	ORF1ab protein
5jilA	-38.7338	Q3HS77	Rat coronavirus	Hemagglutinin-esterase
6zoyB	-37.9133	P0DTC2	Wuhan coronavirus	Spike glycoprotein
2ofzA	-36.9648	P59595	Severe acute respiratory syndrome-related coronavirus Tor2	Nucleocapsid protein
6l5tA	-36.7296	A0A385H8D7	Swine acute diarrhea syndrome coronavirus	Peptidase C16
6vsjA	-33.8111	P11224	Murine hepatitis virus (strain A59)	Spike glycoprotein
6ackC	-33.2235	P59594	Severe acute respiratory syndrome coronavirus	Spike glycoprotein
5n11A	-31.8325	Q4VID6	Human coronavirus strain OC43	Hemagglutinin-esterase
6zgfC	-30.1122	A0A6B9WHD3	Bat coronavirus isolate RaTG13	Spike glycoprotein
5jifB	-28.8133	O92367	Murine coronavirus (strain DVIM)	Hemagglutinin-esterase
5i08C	-28.3981	Q0ZME7	Human coronavirus HKU1 (isolate N5)	Spike glycoprotein,Foldon chimera
6y3yA	-27.2662	Q5MQD1	Human CoV/HKU1	Hemagglutinin-esterase
4h14A	-26.2532	Q1HLC5	neonatal calf diarrhea virus	Spike glycoprotein
2c86A	-25.9064	P69598	infectious bronchitis virus IBV	NUCLEOCAPSID PROTEIN
6m15A	-24.7014	A8JNZ2	Bat coronavirus HKU2	Spike glycoprotein
6u7hB	-23.8565	P15423	Human coronavirus strain 229E	spike glycoprotein
6m16A	-22.1074	A0A2P1G1L3	Swine acute diarrhea syndrome coronavirus	Spike glycoprotein
6qfyA	-21.0859	A0A1Z2WUW0	Porcine hemagglutinating encephalomyelitis coronavirus	Spike glycoprotein
4l3nA	-19.2125	M4SVE7	Human betacoronavirus 2c strain Jordan-N3/2012	S protein

Table S9. List of Coronaviridae family targets, to which dibekacin successfully docked.

PDB ID with Chain	***Score***	***UniProt***	***Organism***	***Protein name***
1wofA	-81.3156	P0C6X7	Severe acute respiratory syndrome coronavirus	3C-like proteinase
5wkkA	-75.4224	K9N638	Middle East respiratory syndrome coronavirus	Orf1a protein

3vb5A	-72.8857	P0C6U8	Severe acute respiratory syndrome coronavirus	3C-like proteinase
5rf4A	-71.6068	P0DTD1	Wuhan coronavirus	3C-like proteinase
5gwzB	-71.1824	P0C6V6	Porcine epidemic diarrhea virus (strain CV777)	PEDV main protease
3d23C	-71.0507	P0C6U3	Human coronavirus HKU1 (isolate N1)	3C-like proteinase
5gwyA	-70.6318	P0C6U6	Human coronavirus NL	main protease
4yluA	-70.0049	V9TU12	Middle East respiratory syndrome coronavirus	ORF1a protein
4xfqA	-67.7452	K4L9I6	porcine epidemic diarrhoea virus	PEDV main protease
2ynaA	-66.7632	P0C6T4	Bat coronavirus HKU4	3C-LIKE PROTEINASE
4zroB	-66.5041	Q98VG9	Feline infectious peritonitis virus (strain 79-1146)	3C-like proteinase
2q6fA	-66.0077	P0C6V5	infectious bronchitis virus IBV	Infectious bronchitis virus (IBV) main protease
5ynoA	-65.8498	K0BWD0	human betacoronavirus 2c EMC	nsp16 protein
5nh0C	-65.49	P0C6X5	Human coronavirus NL	3C-like proteinase
4wmdB	-64.4392	W6A941	Middle East respiratory syndrome coronavirus	ORF1a
4zuhB	-62.9745	R4JK63	porcine epidemic diarrhoea virus	PEDV 3C-Like protease
4yogB	-62.9124	P0C6W3	Bat coronavirus HKU4	3C-like proteinase
5c3nA	-62.7417	V9TU05	Middle East respiratory syndrome coronavirus	ORF1a protein
6jijA	-61.1303	P0C6X9	Murine hepatitis virus (strain A59)	Replicative polyprotein 1ab
2q6dB	-60.7634	P0C6Y3	infectious bronchitis virus IBV	Infectious bronchitis virus (IBV) main protease
6wkpC	-60.4241	P0DTC9	Wuhan coronavirus	Nucleoprotein
2ampA	-59.7982	P0C6Y5	transmissible gastroenteritis virus TGEV	3C-like proteinase
5hyoA	-59.3128	U6BPB2	porcine epidemic diarrhoea virus	PEDV 3CLpro
2zu2A	-58.757	P0C6U2	Human coronavirus strain 229E	3C-like proteinase
6ohwA	-56.2724	Q696P8	Human coronavirus strain OC43	Spike surface glycoprotein
4pt5A	-55.9169	K4LC41	human betacoronavirus 2c EMC	Papain-like protease
4f49A	-55.1921	P0C6V2	Porcine transmissible gastroenteritis coronavirus (STRAIN PURDUE)	3C-like proteinase
5i08C	-50.9057	Q0ZME7	Human coronavirus HKU1 (isolate N5)	Spike glycoprotein,Foldon chimera
6u7kB	-50.7875	Q91AV1	Porcine epidemic diarrhea virus (strain CV777)	Spike glycoprotein
5nfyA	-49.9806	Q1T6X8	SARS coronavirus Frankfurt 1	Polyprotein 1ab
5jilA	-48.7343	Q3HS77	Rat coronavirus	Hemagglutinin-esterase
5yvdB	-48.6489	A0A0U2GPI9	Middle East respiratory syndrome coronavirus	Nsp15
2gecB	-48.3302	P32923	infectious bronchitis virus IBV	Nucleocapsid protein
6q04C	-48.2705	K0BRG7	human betacoronavirus 2c EMC	Spike glycoprotein
2c86A	-47.7526	P69598	infectious bronchitis virus IBV	NUCLEOCAPSID PROTEIN
6zghB	-47.2676	P0DTC2	Wuhan coronavirus	Spike glycoprotein
5w9lB	-46.98	K9N5Q8	Middle East respiratory syndrome coronavirus	Spike glycoprotein
4c7wB	-46.2687	P31614	murine hepatitis virus, MHV	HEMAGGLUTININ-ESTERASE
4lmtA	-46.2568	P33469	Human coronavirus strain OC43	Nucleoprotein
6l5tA	-46.2162	A0A385H8D7	Swine acute diarrhea syndrome coronavirus	Peptidase C16

6acdA	-45.6367	P59594	Severe acute respiratory syndrome coronavirus	Spike glycoprotein
6kl5A	-43.4911	K9N4V7	Middle East respiratory syndrome coronavirus	Nucleoprotein
6nb3A	-42.8179	A0A140AYW5	Middle East respiratory syndrome coronavirus	Spike glycoprotein
6y3yA	-42.3543	Q5MQD1	Human CoV/HKU1	Hemagglutinin-esterase
6nozA	-42.3333	W8QLX4	porcine epidemic diarrhoea virus	Polyprotein
3cl5A	-40.9735	P15776	neonatal calf diarrhea virus	Hemagglutinin-esterase
4h14A	-39.234	Q1HLC5	neonatal calf diarrhea virus	Spike glycoprotein
1sskA	-39.0061	P59595	Severe acute respiratory syndrome coronavirus	Nucleocapsid protein
4rezA	-38.7448	M4STU1	Human betacoronavirus 2c strain Jordan-N3/2012	ORF1ab protein
3jclC	-37.1789	P11224	Murine hepatitis virus (strain A59)	Spike glycoprotein
5jifA	-36.9585	O92367	Murine coronavirus (strain DVIM)	Hemagglutinin-esterase
5n11B	-36.2624	Q4VID6	Human coronavirus strain OC43	Hemagglutinin-esterase
6qfyA	-35.8658	A0A1Z2WUW0	Porcine hemagglutinating encephalomyelitis coronavirus	Spike glycoprotein
6u7hB	-35.0998	P15423	Human coronavirus strain 229E	spike glycoprotein
6m15A	-34.6207	A8JNZ2	Bat coronavirus HKU2	Spike glycoprotein
4l3nA	-34.0063	M4SVE7	Human betacoronavirus 2c strain Jordan-N3/2012	S protein
6m16C	-33.9854	A0A2P1G1L3	Swine acute diarrhea syndrome coronavirus	Spike glycoprotein
4wurA	-28.276	K9N7C7	Betacoronavirus England 1	Papain-like protease
6b7nB	-28.2415	A0A075E3D7	Deltacoronavirus SDCV/USA/Ohio137/2014	Spike protein
6zgfC	-21.8731	A0A6B9WHD3	Bat coronavirus isolate RaTG13	Spike glycoprotein

Table S10. List of Coronaviridae family targets, to which filgotinib successfully docked.

PDB ID with Chain	***Score***	***UniProt***	***Organism***	***Protein name***
4yo9A	-65.277	P0C6W3	Bat coronavirus HKU4	3C-like proteinase
5ynqA	-64.952	K0BWD0	human betacoronavirus 2c EMC	nsp16 protein
2ynaA	-64.2243	P0C6T4	Bat coronavirus HKU4	3C-LIKE PROTEINASE
4wmeB	-63.0127	W6A941	Middle East respiratory syndrome coronavirus	MERS-CoV 3CL protease
6xqtA	-62.2983	P0DTD1	Wuhan coronavirus	3C-like proteinase
3titA	-62.1599	P0C6X7	Severe acute respiratory syndrome coronavirus	SARS coronavirus main protease
2ampA	-60.38	P0C6Y5	transmissible gastroenteritis virus TGEV	3C-like proteinase
5wkkA	-60.1393	K9N638	Middle East respiratory syndrome coronavirus	Orf1a protein
6lnyA	-58.2424	P0C6U8	Severe acute respiratory syndrome coronavirus	Replicase polyprotein 1a
2q6dB	-56.8643	P0C6Y3	infectious bronchitis virus IBV	Infectious bronchitis virus (IBV) main protease
5gwzB	-56.4672	P0C6V6	Porcine epidemic diarrhea virus (strain CV777)	PEDV main protease
5hyoA	-56.2658	U6BPB2	porcine epidemic diarrhoea virus	PEDV 3CLpro
4zroC	-56.2021	Q98VG9	Feline infectious peritonitis virus (strain 79-1146)	3C-like proteinase
5c3nA	-55.5124	V9TU05	Middle East respiratory syndrome coronavirus	ORF1a protein

6ohwB	-53.0063	Q696P8	Human coronavirus strain OC43	Spike surface glycoprotein
6fv2A	-52.8682	P0C6X5	Human coronavirus NL	3C-like proteinase
6nozA	-52.5782	W8QLX4	porcine epidemic diarrhoea virus	Polyprotein
4xfqA	-52.5262	K4L9I6	porcine epidemic diarrhoea virus	PEDV main protease
4f49A	-51.3607	P0C6V2	Porcine transmissible gastroenteritis coronavirus (STRAIN PURDUE)	3C-like proteinase
3tloB	-50.5273	P0C6U6	Human coronavirus NL	3C-like proteinase
3d23D	-49.9212	P0C6U3	Human coronavirus HKU1 (isolate N1)	3C-like proteinase
2q6fB	-49.6843	P0C6V5	infectious bronchitis virus IBV	Infectious bronchitis virus (IBV) main protease
4rspA	-49.5863	V9TU12	Middle East respiratory syndrome coronavirus	Orf1a protein
6wkpC	-49.3325	P0DTC9	Wuhan coronavirus	Nucleoprotein
3cl5A	-49.0483	P15776	neonatal calf diarrhea virus	Hemagglutinin-esterase
2zu2A	-48.4373	P0C6U2	Human coronavirus strain 229E	3C-like proteinase
6jijA	-47.3485	P0C6X9	Murine hepatitis virus (strain A59)	Replicative polyprotein 1ab
2gecB	-47.2582	P32923	infectious bronchitis virus IBV	Nucleocapsid protein
6cs0C	-45.9235	P59594	Severe acute respiratory syndrome coronavirus	Spike glycoprotein,Fibritin
4pt5A	-44.9301	K4LC41	human betacoronavirus 2c EMC	Papain-like protease
4c7wA	-43.99	P31614	murine hepatitis virus, MHV	HEMAGGLUTININ-ESTERASE
6kl6A	-43.4285	K9N4V7	Middle East respiratory syndrome coronavirus	Nucleoprotein
5yvdB	-43.4243	A0A0U2GPI9	Middle East respiratory syndrome coronavirus	Nsp15
5jilA	-43.4186	Q3HS77	Rat coronavirus	Hemagglutinin-esterase
4zuhB	-42.9926	R4JK63	porcine epidemic diarrhoea virus	PEDV 3C-Like protease
6q04C	-41.5292	K0BRG7	human betacoronavirus 2c EMC	Spike glycoprotein
6x2bB	-41.1033	P0DTC2	Wuhan coronavirus	Spike glycoprotein
6u7kB	-40.6523	Q91AV1	Porcine epidemic diarrhea virus (strain CV777)	Spike glycoprotein
4rezA	-40.206	M4STU1	Human betacoronavirus 2c strain Jordan-N3/2012	ORF1ab protein
6l5tA	-39.7703	A0A385H8D7	Swine acute diarrhea syndrome coronavirus	Peptidase C16
4lmtA	-39.568	P33469	Human coronavirus strain OC43	Nucleoprotein
5vyhA	-38.6384	K9N5Q8	Middle East respiratory syndrome coronavirus	S protein
1sskA	-38.5685	P59595	Severe acute respiratory syndrome coronavirus	Nucleocapsid protein
3jclC	-37.2666	P11224	Murine hepatitis virus (strain A59)	Spike glycoprotein
5i08A	-34.8806	Q0ZME7	Human coronavirus HKU1 (isolate N5)	Spike glycoprotein,Foldon chimera
6u7hB	-33.1004	P15423	Human coronavirus strain 229E	spike glycoprotein
5nfyA	-32.2078	Q1T6X8	SARS coronavirus Frankfurt 1	Polyprotein 1ab
6b7nB	-32.0787	A0A075E3D7	Deltacoronavirus SDCV/USA/Ohio137/2014	Spike protein
5n11A	-31.5167	Q4VID6	Human coronavirus strain OC43	Hemagglutinin-esterase
4h14A	-31.4858	Q1HLC5	neonatal calf diarrhea virus	Spike glycoprotein
6nb3A	-31.3886	A0A140AYW5	Middle East respiratory syndrome coronavirus	Spike glycoprotein
4l3nA	-30.2296	M4SVE7	Human betacoronavirus 2c strain Jordan-N3/2012	S protein

5jifB	-30.1166	O92367	Murine coronavirus (strain DVIM)	Hemagglutinin-esterase
6y3yA	-27.254	Q5MQD1	Human CoV/HKU1	Hemagglutinin-esterase
6qfyA	-24.7776	A0A1Z2WUW 0	Porcine hemagglutinating encephalomyelitis coronavirus	Spike glycoprotein
6m15B	-24.7147	A8JNZ2	Bat coronavirus HKU2	Spike glycoprotein
4wurA	-23.9886	K9N7C7	Betacoronavirus England 1	Papain-like protease
6m16A	-23.7096	A0A2P1G1L3	Swine acute diarrhea syndrome coronavirus	Spike glycoprotein
6zgfC	-22.8577	A0A6B9WHD3	Bat coronavirus isolate RaTG13	Spike glycoprotein

Table S11. List of Coronaviridae family targets, to which hydroxychloroquine successfully docked.

PDB ID with Chain	***Score***	***UniProt***	***Organism***	***Protein name***
6w9cC	-61.1833	P0DTD1	Wuhan coronavirus	Non-structural protein 3
4ovzA	-60.1819	P0C6U8	SARS-CoV (Urbani strain)	Papain-like proteinase
4wmeB	-56.5818	W6A941	Middle East respiratory syndrome coronavirus	MERS-CoV 3CL protease
5ynqA	-56.4281	K0BWD0	human betacoronavirus 2c EMC	nsp16 protein
2h2zA	-56.4222	P0C6X7	Severe acute respiratory syndrome coronavirus	Replicase polyprotein 1ab
5gwzB	-55.2076	P0C6V6	Porcine epidemic diarrhea virus (strain CV777)	PEDV main protease
4zroC	-55.2053	Q98VG9	Feline infectious peritonitis virus (strain 79-1146)	3C-like proteinase
6l8qB	-54.0088	K9N5Q8	Middle East respiratory syndrome coronavirus	Spike glycoprotein
2ynaB	-53.4539	P0C6T4	Bat coronavirus HKU4	3C-LIKE PROTEINASE
3d23D	-53.1988	P0C6U3	Human coronavirus HKU1 (isolate N1)	3C-like proteinase
6nzkB	-53.009	Q696P8	Human coronavirus strain OC43	Spike surface glycoprotein
5yn8B	-52.222	K4LC41	human betacoronavirus 2c EMC	nsp10 protein
2q6dB	-51.4532	P0C6Y3	infectious bronchitis virus IBV	Infectious bronchitis virus (IBV) main protease
2q6fB	-51.2221	P0C6V5	infectious bronchitis virus IBV	Infectious bronchitis virus (IBV) main protease
2ampB	-50.8368	P0C6Y5	transmissible gastroenteritis virus TGEV	3C-like proteinase
4yo9A	-50.7433	P0C6W3	Bat coronavirus HKU4	3C-like proteinase
6fv1A	-50.6244	P0C6X5	Human coronavirus NL	3C-like proteinase
5jilA	-49.4252	Q3HS77	Rat coronavirus	Hemagglutinin-esterase
5zqgB	-49.0379	R4JK63	porcine epidemic diarrhoea virus	Non-structural protein
4rezA	-47.6468	M4STU1	Human betacoronavirus 2c strain Jordan-N3/2012	ORF1ab protein
5nfyA	-47.6087	Q1T6X8	SARS coronavirus Frankfurt 1	Polyprotein 1ab
6nozA	-47.5065	W8QLX4	porcine epidemic diarrhoea virus	Polyprotein
6jijA	-47.4385	P0C6X9	Murine hepatitis virus (strain A59)	Replicative polyprotein 1ab
5c3nA	-47.4378	V9TU05	Middle East respiratory syndrome coronavirus	ORF1a protein
2zu2B	-46.601	P0C6U2	Human coronavirus strain 229E	3C-like proteinase
5wkjA	-46.0791	K9N638	Middle East respiratory syndrome coronavirus	Orf1a protein
5gwyA	-44.915	P0C6U6	Human coronavirus NL	main protease
6yi3A	-44.6264	P0DTC9	Wuhan coronavirus	Nucleoprotein
6cs0A	-42.8557	P59594	Severe acute respiratory syndrome coronavirus	Spike glycoprotein,Fibritin

4yluA	-42.8205	V9TU12	Middle East respiratory syndrome coronavirus	ORF1a protein
4wurA	-42.6541	K9N7C7	Betacoronavirus England 1	Papain-like protease
5yvdA	-42.5333	A0A0U2GPI9	Middle East respiratory syndrome coronavirus	Nsp15
6vsjC	-41.4858	P11224	Murine hepatitis virus (strain A59)	Spike glycoprotein
6l70A	-41.421	K4L9I6	porcine epidemic diarrhoea virus	PEDV main protease
6u7kB	-41.2728	Q91AV1	Porcine epidemic diarrhea virus (strain CV777)	Spike glycoprotein
6kl5A	-40.9253	K9N4V7	Middle East respiratory syndrome coronavirus	Nucleoprotein
6vxxC	-40.4093	P0DTC2	Wuhan coronavirus	Spike glycoprotein
4c7lB	-40.2054	P31614	murine hepatitis virus MHV-S	HEMAGGLUTININ-ESTERASE
4f49A	-39.8789	P0C6V2	Porcine transmissible gastroenteritis coronavirus (STRAIN PURDUE)	3C-like proteinase
5hyoB	-39.8541	U6BPB2	porcine epidemic diarrhoea virus	PEDV 3CLpro
6l5tA	-39.0242	A0A385H8D7	Swine acute diarrhea syndrome coronavirus	Peptidase C16
6q07C	-38.8676	K0BRG7	human betacoronavirus 2c EMC	Spike glycoprotein
5i08C	-38.8255	Q0ZME7	Human coronavirus HKU1 (isolate N5)	Spike glycoprotein,Foldon chimera
6jx7C	-38.7656	C6GHB7	FIPV	Feline Infectious Peritonitis Virus Spike Protein
3cl5A	-37.931	P15776	neonatal calf diarrhea virus	Hemagglutinin-esterase
6y3yB	-36.312	Q5MQD1	Human CoV/HKU1	Hemagglutinin-esterase
2c86A	-36.2548	P69598	infectious bronchitis virus IBV	NUCLEOCAPSID PROTEIN
6nb3A	-34.8464	A0A140AYW5	Middle East respiratory syndrome coronavirus	Spike glycoprotein
4l3nA	-34.5001	M4SVE7	Human betacoronavirus 2c strain Jordan-N3/2012	S protein
6m16B	-33.6907	A0A2P1G1L3	Swine acute diarrhea syndrome coronavirus	Spike glycoprotein
2gecB	-33.5273	P32923	infectious bronchitis virus IBV	Nucleocapsid protein
5jifA	-33.1037	O92367	Murine coronavirus (strain DVIM)	Hemagglutinin-esterase
6zgfC	-31.8418	A0A6B9WHD3	Bat coronavirus isolate RaTG13	Spike glycoprotein
6m15B	-31.7014	A8JNZ2	Bat coronavirus HKU2	Spike glycoprotein
6vv5B	-31.1031	S5RJN4	porcine epidemic diarrhoea virus	Spike glycoprotein
4lmtA	-30.5085	P33469	Human coronavirus strain OC43	Nucleoprotein
6qfyA	-29.9976	A0A1Z2WUW0	Porcine hemagglutinating encephalomyelitis coronavirus	Spike glycoprotein
2ofzA	-29.8936	P59595	Severe acute respiratory syndrome-related coronavirus Tor2	Nucleocapsid protein
6u7hB	-28.0016	P15423	Human coronavirus strain 229E	spike glycoprotein
4h14A	-26.5107	Q1HLC5	neonatal calf diarrhea virus	Spike glycoprotein
6b7nA	-25.856	A0A075E3D7	Deltacoronavirus SDCV/USA/Ohio137/2014	Spike protein
6bfuB	-22.1371	A0A140ESF1	Porcine deltacoronavirus	Spike protein
5n11A	-15.1193	Q4VID6	Human coronavirus strain OC43	Hemagglutinin-esterase

Table S12. List of Coronaviridae family targets, to which ivermectin B1a successfully docked.

PDB ID with Chain	***Score***	***UniProt***	***Organism***	***Protein name***
5nh0B	-84.8233	P0C6X5	Human coronavirus NL	3C-like proteinase

3e91A	-81.8493	P0C6X7	Severe acute respiratory syndrome coronavirus	3C-like proteinase
3vb3B	-80.6177	P0C6U8	Severe acute respiratory syndrome coronavirus	3C-like proteinase
6wnpA	-79.9472	P0DTD1	Wuhan coronavirus	3C-like proteinase
4wmeA	-77.9929	W6A941	Middle East respiratory syndrome coronavirus	MERS-CoV 3CL protease
5ynpA	-76.9035	K0BWD0	human betacoronavirus 2c EMC	nsp16 protein
4yoiB	-73.7192	P0C6W3	Bat coronavirus HKU4	3C-like proteinase
4xfqA	-72.4992	K4L9I6	porcine epidemic diarrhoea virus	PEDV main protease
2ynbA	-70.4812	P0C6T4	Bat coronavirus HKU4	3C-LIKE PROTEINASE
5gwyA	-70.3562	P0C6U6	Human coronavirus NL	main protease
5eu8A	-70.068	Q98VG9	FIPV	main protease
1p9uA	-70.0275	P0C6Y5	transmissible gastroenteritis virus TGEV	putative coronavirus nsp2 (3CL-PRO)
1p9sA	-69.5677	P0C6U2	Human coronavirus strain 229E	Replicase polyprotein 1ab
5wkmA	-68.5431	K9N638	Middle East respiratory syndrome coronavirus	Orf1a protein
2q6dA	-65.4669	P0C6Y3	infectious bronchitis virus IBV	Infectious bronchitis virus (IBV) main protease
4zuhB	-64.855	R4JK63	porcine epidemic diarrhoea virus	PEDV 3C-Like protease
3d23B	-64.2552	P0C6U3	Human coronavirus HKU1 (isolate N1)	3C-like proteinase
4f49A	-61.0468	P0C6V2	Porcine transmissible gastroenteritis coronavirus (STRAIN PURDUE)	3C-like proteinase
5hyoA	-60.5464	U6BPB2	porcine epidemic diarrhoea virus	PEDV 3CLpro
5c3nA	-59.9143	V9TU05	Middle East respiratory syndrome coronavirus	ORF1a protein
5gwzA	-59.5047	P0C6V6	Porcine epidemic diarrhea virus (strain CV777)	PEDV main protease
6jijA	-57.2848	P0C6X9	Murine hepatitis virus (strain A59)	Replicative polyprotein 1ab
2q6fB	-56.4901	P0C6V5	infectious bronchitis virus IBV	Infectious bronchitis virus (IBV) main protease
4yluA	-56.3579	V9TU12	Middle East respiratory syndrome coronavirus	ORF1a protein

Table S13. List of Coronaviridae family targets, to which ivermectin B1b successfully docked.

PDB ID with Chain	***Score***	***UniProt***	***Organism***	***Protein name***
5rgqA	-88.4984	P0DTD1	Wuhan coronavirus	3C-like proteinase
4wmeA	-86.992	W6A941	Middle East respiratory syndrome coronavirus	MERS-CoV 3CL protease
6fv1A	-76.6498	P0C6X5	Human coronavirus NL	3C-like proteinase
6xhlB	-76.2684	P0C6U8	Severe acute respiratory syndrome coronavirus	3C-like proteinase
5n19A	-75.1864	P0C6X7	Severe acute respiratory syndrome coronavirus	SARS coronavirus main protease
5gwyA	-73.0402	P0C6U6	Human coronavirus NL	main protease
2ynaA	-72.6385	P0C6T4	Bat coronavirus HKU4	3C-LIKE PROTEINASE
2zu2B	-72.073	P0C6U2	Human coronavirus strain 229E	3C-like proteinase
5ynbA	-71.2601	K0BWD0	human betacoronavirus 2c EMC	nsp16 protein
5wkkA	-67.4222	K9N638	Middle East respiratory syndrome coronavirus	Orf1a protein
6ohwC	-65.2965	Q696P8	Human coronavirus strain OC43	Spike surface glycoprotein

4zroC	-64.5277	Q98VG9	Feline infectious peritonitis virus (strain 79-1146)	3C-like proteinase
4yogA	-64.2198	P0C6W3	Bat coronavirus HKU4	3C-like proteinase
6jijA	-62.7192	P0C6X9	Murine hepatitis virus (strain A59)	Replicative polyprotein 1ab
1p9uA	-62.5042	P0C6Y5	transmissible gastroenteritis virus TGEV	putative coronavirus nsp2 (3CL-PRO)
4f49A	-62.1784	P0C6V2	Porcine transmissible gastroenteritis coronavirus (STRAIN PURDUE)	3C-like proteinase
4xfqA	-60.7577	K4L9I6	porcine epidemic diarrhoea virus	PEDV main protease
5gwzA	-59.5835	P0C6V6	Porcine epidemic diarrhea virus (strain CV777)	PEDV main protease
5zqgB	-59.5004	R4JK63	porcine epidemic diarrhoea virus	Non-structural protein
2q6dA	-58.9215	P0C6Y3	infectious bronchitis virus IBV	Infectious bronchitis virus (IBV) main protease
5c3nB	-58.412	V9TU05	Middle East respiratory syndrome coronavirus	ORF1a protein
5hyoA	-54.6851	U6BPB2	porcine epidemic diarrhoea virus	PEDV 3CLpro
4rspA	-54.476	V9TU12	Middle East respiratory syndrome coronavirus	Orf1a protein
3d23A	-53.6038	P0C6U3	Human coronavirus HKU1 (isolate N1)	3C-like proteinase
2q6fB	-42.4489	P0C6V5	infectious bronchitis virus IBV	Infectious bronchitis virus (IBV) main protease

Table S14. List of Coronaviridae family targets, to which micronomicin successfully docked.

PDB ID with Chain	***Score***	***UniProt***	***Organism***	***Protein name***
5ynqA	-79.1253	K0BWD0	human betacoronavirus 2c EMC	nsp16 protein
5gwzB	-78.7726	P0C6V6	Porcine epidemic diarrhea virus (strain CV777)	PEDV main protease
5gwyA	-78.5667	P0C6U6	Human coronavirus NL	main protease
5rghA	-76.3171	P0DTD1	Wuhan coronavirus	3C-like proteinase
1wofA	-75.9637	P0C6X7	Severe acute respiratory syndrome coronavirus	3C-like proteinase
6xhnB	-74.0899	P0C6U8	Severe acute respiratory syndrome coronavirus	3C-like proteinase
3d23D	-72.1551	P0C6U3	Human coronavirus HKU1 (isolate N1)	3C-like proteinase
4zroA	-70.1222	Q98VG9	Feline infectious peritonitis virus (strain 79-1146)	3C-like proteinase
2q6fA	-67.5934	P0C6V5	infectious bronchitis virus IBV	Infectious bronchitis virus (IBV) main protease
5nh0A	-66.9786	P0C6X5	Human coronavirus NL	3C-like proteinase
2q6dA	-66.9523	P0C6Y3	infectious bronchitis virus IBV	Infectious bronchitis virus (IBV) main protease
5hyoB	-65.736	U6BPB2	porcine epidemic diarrhoea virus	PEDV 3CLpro
2ynbA	-64.9202	P0C6T4	Bat coronavirus HKU4	3C-LIKE PROTEINASE
6ohwB	-64.8766	Q696P8	Human coronavirus strain OC43	Spike surface glycoprotein
2zu2B	-64.3794	P0C6U2	Human coronavirus strain 229E	3C-like proteinase
4wmeA	-64.3201	W6A941	Middle East respiratory syndrome coronavirus	MERS-CoV 3CL protease
4yojB	-63.8351	P0C6W3	Bat coronavirus HKU4	3C-like proteinase
1p9uB	-62.9649	P0C6Y5	transmissible gastroenteritis virus TGEV	putative coronavirus nsp2 (3CL-PRO)
6jijA	-62.8811	P0C6X9	Murine hepatitis virus (strain A59)	Replicative polyprotein 1ab
4xfqA	-62.5156	K4L9I6	porcine epidemic diarrhoea virus	PEDV main protease

5c3nA	-61.0449	V9TU05	Middle East respiratory syndrome coronavirus	ORF1a protein
6wkpC	-60.105	P0DTC9	Wuhan coronavirus	Nucleoprotein
4pt5A	-58.7848	K4LC41	human betacoronavirus 2c EMC	Papain-like protease
5zqgB	-57.5802	R4JK63	porcine epidemic diarrhoea virus	Non-structural protein
4f49A	-57.4267	P0C6V2	Porcine transmissible gastroenteritis coronavirus (STRAIN PURDUE)	3C-like proteinase
5wkjA	-56.9041	K9N638	Middle East respiratory syndrome coronavirus	Orf1a protein
4rspA	-56.3009	V9TU12	Middle East respiratory syndrome coronavirus	Orf1a protein
5yvdA	-53.0148	A0A0U2GPI9	Middle East respiratory syndrome coronavirus	Nsp15
6nozA	-52.3658	W8QLX4	porcine epidemic diarrhoea virus	Polyprotein
6kl2A	-51.3014	K9N4V7	Middle East respiratory syndrome coronavirus	Nucleoprotein
4lm9A	-50.9075	P33469	Human coronavirus strain OC43	Nucleoprotein
5nfyA	-50.6473	Q1T6X8	SARS coronavirus Frankfurt 1	Polyprotein 1ab
1sskA	-49.6457	P59595	Severe acute respiratory syndrome coronavirus	Nucleocapsid protein
2gecB	-49.1914	P32923	infectious bronchitis virus IBV	Nucleocapsid protein
6l5tA	-48.6748	A0A385H8D7	Swine acute diarrhea syndrome coronavirus	Peptidase C16
6u7kB	-47.1255	Q91AV1	Porcine epidemic diarrhea virus (strain CV777)	Spike glycoprotein
4rezA	-46.9495	M4STU1	Human betacoronavirus 2c strain Jordan-N3/2012	ORF1ab protein
4c7wA	-46.155	P31614	murine hepatitis virus, MHV	HEMAGGLUTININ-ESTERASE
3cl5A	-45.6682	P15776	neonatal calf diarrhea virus	Hemagglutinin-esterase
5w9iJ	-44.5555	K9N5Q8	Middle East respiratory syndrome coronavirus	Spike glycoprotein
5jilA	-44.2153	Q3HS77	Rat coronavirus	Hemagglutinin-esterase
6u7hB	-43.3106	P15423	Human coronavirus strain 229E	spike glycoprotein
5i08C	-43.0832	Q0ZME7	Human coronavirus HKU1 (isolate N5)	Spike glycoprotein,Foldon chimera
6zoxA	-42.6434	P0DTC2	Wuhan coronavirus	Spike glycoprotein
5jifB	-41.3816	O92367	Murine coronavirus (strain DVIM)	Hemagglutinin-esterase
5n11A	-36.1514	Q4VID6	Human coronavirus strain OC43	Hemagglutinin-esterase
6m15C	-36.0786	A8JNZ2	Bat coronavirus HKU2	Spike glycoprotein
6m16B	-36.05	A0A2P1G1L3	Swine acute diarrhea syndrome coronavirus	Spike glycoprotein
6qfyA	-35.7518	A0A1Z2WUW0	Porcine hemagglutinating encephalomyelitis coronavirus	Spike glycoprotein
6y3yA	-34.2048	Q5MQD1	Human CoV/HKU1	Hemagglutinin-esterase
5do2A	-33.1872	K0BRG7	Middle East respiratory syndrome coronavirus	S protein
6acgA	-32.2708	P59594	Severe acute respiratory syndrome coronavirus	Spike glycoprotein
4h14A	-32.0354	Q1HLC5	neonatal calf diarrhea virus	Spike glycoprotein
2c86A	-31.2789	P69598	infectious bronchitis virus IBV	NUCLEOCAPSID PROTEIN
6nb3A	-29.5657	A0A140AYW5	Middle East respiratory syndrome coronavirus	Spike glycoprotein
6vsjA	-23.8848	P11224	Murine hepatitis virus (strain A59)	Spike glycoprotein
6zgfC	-16.5115	A0A6B9WHD3	Bat coronavirus isolate RaTG13	Spike glycoprotein

6b7nB	-15.3145	A0A075E3D7	Deltacoronavirus SDCV/USA/Ohio137/2014	Spike protein

Table S15. List of Coronaviridae family targets, to which nebivolol successfully docked.

PDB ID with Chain	***Score***	***UniProt***	***Organism***	***Protein name***
5gwzB	-62.0548	P0C6V6	Porcine epidemic diarrhea virus (strain CV777)	PEDV main protease
3d23C	-60.7946	P0C6U3	Human coronavirus HKU1 (isolate N1)	3C-like proteinase
4wmeA	-60.2914	W6A941	Middle East respiratory syndrome coronavirus	MERS-CoV 3CL protease
6xbiB	-60.2077	P0DTD1	Wuhan coronavirus	3C-like proteinase
1uk3A	-57.8652	P0C6X7	Severe acute respiratory syndrome coronavirus	3C-like proteinase
5ynoA	-57.7867	K0BWD0	human betacoronavirus 2c EMC	nsp16 protein
6lnyA	-57.6664	P0C6U8	Severe acute respiratory syndrome coronavirus	Replicase polyprotein 1a
3tloB	-57.404	P0C6U6	Human coronavirus NL	3C-like proteinase
4pt5A	-56.5874	K4LC41	human betacoronavirus 2c EMC	Papain-like protease
6nozA	-56.4812	W8QLX4	porcine epidemic diarrhoea virus	Polyprotein
5zqgB	-55.8307	R4JK63	porcine epidemic diarrhoea virus	Non-structural protein
4yo9A	-53.5708	P0C6W3	Bat coronavirus HKU4	3C-like proteinase
5wkjA	-52.6246	K9N638	Middle East respiratory syndrome coronavirus	Orf1a protein
2ynaB	-51.7494	P0C6T4	Bat coronavirus HKU4	3C-LIKE PROTEINASE
2q6dA	-50.6565	P0C6Y3	infectious bronchitis virus IBV	Infectious bronchitis virus (IBV) main protease
2q6fA	-50.5706	P0C6V5	infectious bronchitis virus IBV	Infectious bronchitis virus (IBV) main protease
5c3nA	-50.528	V9TU05	Middle East respiratory syndrome coronavirus	ORF1a protein
6jijA	-49.9971	P0C6X9	Murine hepatitis virus (strain A59)	Replicative polyprotein 1ab
6wkpC	-49.7589	P0DTC9	Wuhan coronavirus	Nucleoprotein
4xfqB	-48.7067	K4L9I6	porcine epidemic diarrhoea virus	PEDV main protease
6ohwC	-48.3607	Q696P8	Human coronavirus strain OC43	Spike surface glycoprotein
1lvoB	-47.705	P0C6Y5	transmissible gastroenteritis virus TGEV	Replicase, hydrolase domain
1p9sA	-47.1689	P0C6U2	Human coronavirus strain 229E	Replicase polyprotein 1ab
5yvdA	-45.2904	A0A0U2GPI9	Middle East respiratory syndrome coronavirus	Nsp15
4zroC	-44.1991	Q98VG9	Feline infectious peritonitis virus (strain 79-1146)	3C-like proteinase
6fv2A	-44.1726	P0C6X5	Human coronavirus NL	3C-like proteinase
4rspA	-43.1991	V9TU12	Middle East respiratory syndrome coronavirus	Orf1a protein
6q06B	-42.8259	K0BRG7	human betacoronavirus 2c EMC	Spike glycoprotein
4c7lA	-42.8027	P31614	murine hepatitis virus MHV-S	HEMAGGLUTININ-ESTERASE
6u7kB	-42.7397	Q91AV1	Porcine epidemic diarrhea virus (strain CV777)	Spike glycoprotein
6l5tA	-42.6836	A0A385H8D7	Swine acute diarrhea syndrome coronavirus	Peptidase C16
6cs0B	-41.827	P59594	Severe acute respiratory syndrome coronavirus	Spike glycoprotein,Fibritin
5i08A	-41.5683	Q0ZME7	Human coronavirus HKU1 (isolate N5)	Spike glycoprotein,Foldon chimera

5hyoA	-41.4853	U6BPB2	porcine epidemic diarrhoea virus	PEDV 3CLpro
6pz8E	-40.7764	K9N5Q8	Middle East respiratory syndrome coronavirus	S protein
6kl5A	-40.4404	K9N4V7	Middle East respiratory syndrome coronavirus	Nucleoprotein
5n11B	-40.3945	Q4VID6	Human coronavirus strain OC43	Hemagglutinin-esterase
6zoyA	-38.1016	P0DTC2	Wuhan coronavirus	Spike glycoprotein
6m15B	-37.8566	A8JNZ2	Bat coronavirus HKU2	Spike glycoprotein
5jilA	-37.072	Q3HS77	Rat coronavirus	Hemagglutinin-esterase
4lmtA	-36.6819	P33469	Human coronavirus strain OC43	Nucleoprotein
2c86A	-35.9796	P69598	infectious bronchitis virus IBV	NUCLEOCAPSID PROTEIN
3cl5A	-35.3779	P15776	neonatal calf diarrhea virus	Hemagglutinin-esterase
6y3yA	-34.8784	Q5MQD1	Human CoV/HKU1	Hemagglutinin-esterase
4f49A	-34.7999	P0C6V2	Porcine transmissible gastroenteritis coronavirus (STRAIN PURDUE)	3C-like proteinase
1sskA	-34.5847	P59595	Severe acute respiratory syndrome coronavirus	Nucleocapsid protein
4rf1A	-34.208	M4STU1	Human betacoronavirus 2c strain Jordan-N3/2012	ORF1ab protein
4wurA	-33.5927	K9N7C7	Betacoronavirus England 1	Papain-like protease
6nb4C	-33.5901	A0A140AYW5	Middle East respiratory syndrome coronavirus	Spike glycoprotein
6zgfC	-33.2876	A0A6B9WHD3	Bat coronavirus isolate RaTG13	Spike glycoprotein
5nfyA	-32.0914	Q1T6X8	SARS coronavirus Frankfurt 1	Polyprotein 1ab
2gecB	-31.9527	P32923	infectious bronchitis virus IBV	Nucleocapsid protein
5jifB	-31.0165	O92367	Murine coronavirus (strain DVIM)	Hemagglutinin-esterase
6m16B	-30.4187	A0A2P1G1L3	Swine acute diarrhea syndrome coronavirus	Spike glycoprotein
6u7hB	-29.0618	P15423	Human coronavirus strain 229E	spike glycoprotein
6jx7C	-28.7884	C6GHB7	FIPV	Feline Infectious Peritonitis Virus Spike Protein
6vsjA	-28.5832	P11224	Murine hepatitis virus (strain A59)	Spike glycoprotein
4l3nA	-25.7261	M4SVE7	Human betacoronavirus 2c strain Jordan-N3/2012	S protein
6b7nB	-25.2906	A0A075E3D7	Deltacoronavirus SDCV/USA/Ohio137/2014	Spike protein
4h14A	-24.3983	Q1HLC5	neonatal calf diarrhea virus	Spike glycoprotein
6bfuA	-24.1396	A0A140ESF1	Porcine deltacoronavirus	Spike protein
6qfyA	-22.623	A0A1Z2WUW0	Porcine hemagglutinating encephalomyelitis coronavirus	Spike glycoprotein
6vv5B	-14.0774	S5RJN4	porcine epidemic diarrhoea virus	Spike glycoprotein

Table S16. List of Coronaviridae family targets, to which pexidartinib successfully docked.

PDB ID with Chain	***Score***	***UniProt***	***Organism***	***Protein name***
4wmeA	-50.4923	W6A941	Middle East respiratory syndrome coronavirus	MERS-CoV 3CL protease
4ow0A	-49.5934	P0C6U8	SARS-CoV (Urbani strain)	papain-like protease
7c2jA	-48.9168	P0DTD1	Wuhan coronavirus	2'-O-methyltransferase
5b6oA	-44.6071	P0C6X7	Severe acute respiratory syndrome coronavirus	3C-like proteinase
4yo9A	-44.5033	P0C6W3	Bat coronavirus HKU4	3C-like proteinase
5ynfA	-42.5796	K0BWD0	human betacoronavirus 2c EMC	nsp16 protein

2ynbA	-42.0074	P0C6T4	Bat coronavirus HKU4	3C-LIKE PROTEINASE
6nzkB	-41.7014	Q696P8	Human coronavirus strain OC43	Spike surface glycoprotein
5wkjA	-39.3044	K9N638	Middle East respiratory syndrome coronavirus	Orf1a protein
6wkpC	-39.0376	P0DTC9	Wuhan coronavirus	Nucleoprotein
6jijB	-38.9526	P0C6X9	Murine hepatitis virus (strain A59)	Replicative polyprotein 1ab
5gwyA	-38.8454	P0C6U6	Human coronavirus NL	main protease
6l70B	-38.458	K4L9I6	porcine epidemic diarrhoea virus	PEDV main protease
3d23B	-38.3987	P0C6U3	Human coronavirus HKU1 (isolate N1)	3C-like proteinase
1lvoB	-38.0787	P0C6Y5	transmissible gastroenteritis virus TGEV	Replicase, hydrolase domain
5c3nA	-37.2519	V9TU05	Middle East respiratory syndrome coronavirus	ORF1a protein
4zuhA	-37.062	R4JK63	porcine epidemic diarrhoea virus	PEDV 3C-Like protease
2q6fB	-37.039	P0C6V5	infectious bronchitis virus IBV	Infectious bronchitis virus (IBV) main protease
4zroA	-36.9848	Q98VG9	Feline infectious peritonitis virus (strain 79-1146)	3C-like proteinase
4pt5A	-36.734	K4LC41	human betacoronavirus 2c EMC	Papain-like protease
5gwzB	-36.6978	P0C6V6	Porcine epidemic diarrhea virus (strain CV777)	PEDV main protease
4f49A	-36.5002	P0C6V2	Porcine transmissible gastroenteritis coronavirus (STRAIN PURDUE)	3C-like proteinase
6fv1A	-36.2073	P0C6X5	Human coronavirus NL	3C-like proteinase
4c7wB	-35.926	P31614	murine hepatitis virus, MHV	HEMAGGLUTININ-ESTERASE
5yvdA	-35.6482	A0A0U2GPI9	Middle East respiratory syndrome coronavirus	Nsp15
4yluB	-35.3555	V9TU12	Middle East respiratory syndrome coronavirus	ORF1a protein
5jilA	-35.1373	Q3HS77	Rat coronavirus	Hemagglutinin-esterase
6nozA	-35.063	W8QLX4	porcine epidemic diarrhoea virus	Polyprotein
2q6dB	-34.8603	P0C6Y3	infectious bronchitis virus IBV	Infectious bronchitis virus (IBV) main protease
6u7kB	-33.9951	Q91AV1	Porcine epidemic diarrhea virus (strain CV777)	Spike glycoprotein
5nfyA	-33.8499	Q1T6X8	SARS coronavirus Frankfurt 1	Polyprotein 1ab
6kl6A	-33.7945	K9N4V7	Middle East respiratory syndrome coronavirus	Nucleoprotein
6zozB	-32.7538	P0DTC2	Wuhan coronavirus	Spike glycoprotein
1p9sA	-32.3146	P0C6U2	Human coronavirus strain 229E	Replicase polyprotein 1ab
6l5tA	-30.3324	A0A385H8D7	Swine acute diarrhea syndrome coronavirus	Peptidase C16
3cl5A	-29.8743	P15776	neonatal calf diarrhea virus	Hemagglutinin-esterase
6pz8I	-29.5426	K9N5Q8	Middle East respiratory syndrome coronavirus	S protein
5hyoA	-29.2183	U6BPB2	porcine epidemic diarrhoea virus	PEDV 3CLpro
2c86A	-29.2124	P69598	infectious bronchitis virus IBV	NUCLEOCAPSID PROTEIN
3jclC	-27.5703	P11224	Murine hepatitis virus (strain A59)	Spike glycoprotein
4rezA	-27.4688	M4STU1	Human betacoronavirus 2c strain Jordan-N3/2012	ORF1ab protein
2gecB	-26.7109	P32923	infectious bronchitis virus IBV	Nucleocapsid protein
2ofzA	-26.4379	P59595	Severe acute respiratory syndrome-related coronavirus Tor2	Nucleocapsid protein

5jifB	-26.3445	O92367	Murine coronavirus (strain DVIM)	Hemagglutinin-esterase
6nb7C	-26.2534	P59594	Severe acute respiratory syndrome coronavirus	Spike glycoprotein
6u7hB	-26.2056	P15423	Human coronavirus strain 229E	spike glycoprotein
5i08A	-25.1074	Q0ZME7	Human coronavirus HKU1 (isolate N5)	Spike glycoprotein,Foldon chimera
4lmcA	-25.0784	P33469	Human coronavirus strain OC43	Nucleoprotein
6qfyA	-24.8144	A0A1Z2WUW0	Porcine hemagglutinating encephalomyelitis coronavirus	Spike glycoprotein
6q05A	-23.7657	K0BRG7	human betacoronavirus 2c EMC	Spike glycoprotein
5n11A	-23.6502	Q4VID6	Human coronavirus strain OC43	Hemagglutinin-esterase
6y3yA	-23.4314	Q5MQD1	Human CoV/HKU1	Hemagglutinin-esterase
6nb3A	-23.1964	A0A140AYW5	Middle East respiratory syndrome coronavirus	Spike glycoprotein
6zgfC	-22.6448	A0A6B9WHD3	Bat coronavirus isolate RaTG13	Spike glycoprotein
6b7nB	-21.8709	A0A075E3D7	Deltacoronavirus SDCV/USA/Ohio137/2014	Spike protein
6m15A	-21.1653	A8JNZ2	Bat coronavirus HKU2	Spike glycoprotein
6m16B	-21.1052	A0A2P1G1L3	Swine acute diarrhea syndrome coronavirus	Spike glycoprotein
4h14A	-19.0027	Q1HLC5	neonatal calf diarrhea virus	Spike glycoprotein
4l3nA	-12.0024	M4SVE7	Human betacoronavirus 2c strain Jordan-N3/2012	S protein

Table S17. List of Coronaviridae family targets, to which propafenone successfully docked.

PDB ID with Chain	***Score***	***UniProt***	***Organism***	***Protein name***
6w9cB	-63.3677	P0DTD1	Wuhan coronavirus	Non-structural protein 3
1p9uB	-57.6165	P0C6Y5	transmissible gastroenteritis virus TGEV	putative coronavirus nsp2 (3CL-PRO)
2vj1B	-57.3554	P0C6X7	Severe acute respiratory syndrome coronavirus	SARS CORONAVIRUS MAIN PROTEINASE
6lo0A	-55.1631	P0C6U8	Severe acute respiratory syndrome coronavirus	Replicase polyprotein 1a
6l8qB	-53.578	K9N5Q8	Middle East respiratory syndrome coronavirus	Spike glycoprotein
6crxA	-51.8525	P59594	Severe acute respiratory syndrome coronavirus	Spike glycoprotein,Fibritin
4pt5A	-49.4908	K4LC41	human betacoronavirus 2c EMC	Papain-like protease
4yo9B	-47.4696	P0C6W3	Bat coronavirus HKU4	3C-like proteinase
6zp2C	-45.2797	P0DTC2	Wuhan coronavirus	Spike glycoprotein
3d23B	-45.1587	P0C6U3	Human coronavirus HKU1 (isolate N1)	3C-like proteinase
5ynqA	-45.0107	K0BWD0	human betacoronavirus 2c EMC	nsp16 protein
2ynaA	-44.2336	P0C6T4	Bat coronavirus HKU4	3C-LIKE PROTEINASE
4wmeA	-44.1596	W6A941	Middle East respiratory syndrome coronavirus	MERS-CoV 3CL protease
6u7kB	-43.6109	Q91AV1	Porcine epidemic diarrhea virus (strain CV777)	Spike glycoprotein
6ohwA	-43.3709	Q696P8	Human coronavirus strain OC43	Spike surface glycoprotein
6fv2A	-43.2361	P0C6X5	Human coronavirus NL	3C-like proteinase
6wkpA	-43.2311	P0DTC9	Wuhan coronavirus	Nucleoprotein
2c86A	-43.1462	P69598	infectious bronchitis virus IBV	NUCLEOCAPSID PROTEIN
6q04C	-42.8429	K0BRG7	human betacoronavirus 2c EMC	Spike glycoprotein

4f49A	-42.6504	P0C6V2	Porcine transmissible gastroenteritis coronavirus (STRAIN PURDUE)	3C-like proteinase
6zgfC	-42.4693	A0A6B9WHD3	Bat coronavirus isolate RaTG13	Spike glycoprotein
5yvdB	-41.7873	A0A0U2GPI9	Middle East respiratory syndrome coronavirus	Nsp15
6jijA	-41.0844	P0C6X9	Murine hepatitis virus (strain A59)	Replicative polyprotein 1ab
4c7wA	-40.0881	P31614	murine hepatitis virus, MHV	HEMAGGLUTININ-ESTERASE
4xfqB	-40.0037	K4L9I6	porcine epidemic diarrhoea virus	PEDV main protease
2q6dB	-39.9808	P0C6Y3	infectious bronchitis virus IBV	Infectious bronchitis virus (IBV) main protease
4zuhA	-39.7354	R4JK63	porcine epidemic diarrhoea virus	PEDV 3C-Like protease
4rezA	-38.7644	M4STU1	Human betacoronavirus 2c strain Jordan-N3/2012	ORF1ab protein
6kl5A	-38.6741	K9N4V7	Middle East respiratory syndrome coronavirus	Nucleoprotein
5gwzA	-37.9605	P0C6V6	Porcine epidemic diarrhea virus (strain CV777)	PEDV main protease
6b7nA	-37.8042	A0A075E3D7	Deltacoronavirus SDCV/USA/Ohio137/2014	Spike protein
6m15A	-37.4851	A8JNZ2	Bat coronavirus HKU2	Spike glycoprotein
4zroA	-37.042	Q98VG9	Feline infectious peritonitis virus (strain 79-1146)	3C-like proteinase
4yluA	-36.9586	V9TU12	Middle East respiratory syndrome coronavirus	ORF1a protein
6nb4C	-36.4911	A0A140AYW5	Middle East respiratory syndrome coronavirus	Spike glycoprotein
3tloB	-36.252	P0C6U6	Human coronavirus NL	3C-like proteinase
3cl5A	-35.8849	P15776	neonatal calf diarrhea virus	Hemagglutinin-esterase
5wklA	-35.2315	K9N638	Middle East respiratory syndrome coronavirus	Orf1a protein
6m16C	-35.1476	A0A2P1G1L3	Swine acute diarrhea syndrome coronavirus	Spike glycoprotein
6nozA	-35.1329	W8QLX4	porcine epidemic diarrhoea virus	Polyprotein
5c3nA	-34.9426	V9TU05	Middle East respiratory syndrome coronavirus	ORF1a protein
4h14A	-34.8688	Q1HLC5	neonatal calf diarrhea virus	Spike glycoprotein
6jx7C	-34.4044	C6GHB7	FIPV	Feline Infectious Peritonitis Virus Spike Protein
5hyoB	-34.305	U6BPB2	porcine epidemic diarrhoea virus	PEDV 3CLpro
1p9sA	-33.9983	P0C6U2	Human coronavirus strain 229E	Replicase polyprotein 1ab
2q6fA	-32.6691	P0C6V5	infectious bronchitis virus IBV	Infectious bronchitis virus (IBV) main protease
6bfuA	-31.4926	A0A140ESF1	Porcine deltacoronavirus	Spike protein
4lmcA	-31.3822	P33469	Human coronavirus strain OC43	Nucleoprotein
6y3yA	-30.8614	Q5MQD1	Human CoV/HKU1	Hemagglutinin-esterase
3jclA	-30.2918	P11224	Murine hepatitis virus (strain A59)	Spike glycoprotein
4wurA	-29.7843	K9N7C7	Betacoronavirus England 1	Papain-like protease
5nfyA	-29.1367	Q1T6X8	SARS coronavirus Frankfurt 1	Polyprotein 1ab
6l5tA	-29.0638	A0A385H8D7	Swine acute diarrhea syndrome coronavirus	Peptidase C16
6vv5B	-28.711	S5RJN4	porcine epidemic diarrhoea virus	Spike glycoprotein
6u7hB	-28.4927	P15423	Human coronavirus strain 229E	spike glycoprotein
2ofzA	-27.8652	P59595	Severe acute respiratory syndrome-related coronavirus Tor2	Nucleocapsid protein
4zxnA	-27.3744	Q3HS77	Rat coronavirus (strain NJ)	HE protein

5i08C	-26.9983	Q0ZME7	Human coronavirus HKU1 (isolate N5)	Spike glycoprotein,Foldon chimera
5n11A	-26.7304	Q4VID6	Human coronavirus strain OC43	Hemagglutinin-esterase
6qfyA	-23.3252	A0A1Z2WUW0	Porcine hemagglutinating encephalomyelitis coronavirus	Spike glycoprotein
5jifB	-16.1822	O92367	Murine coronavirus (strain DVIM)	Hemagglutinin-esterase

Table S18. List of Coronaviridae family targets, to which selamectin successfully docked.

PDB ID with Chain	***Score***	***UniProt***	***Organism***	***Protein name***
6xqsA	-73.7824	P0DTD1	Wuhan coronavirus	3C-like proteinase
5n5oA	-73.465	P0C6X7	Severe acute respiratory syndrome coronavirus	Replicase polyprotein 1ab
2ynaA	-72.1342	P0C6T4	Bat coronavirus HKU4	3C-LIKE PROTEINASE
6xhnB	-70.3328	P0C6U8	Severe acute respiratory syndrome coronavirus	3C-like proteinase
5nh0B	-65.3721	P0C6X5	Human coronavirus NL	3C-like proteinase
4wmeA	-65.0155	W6A941	Middle East respiratory syndrome coronavirus	MERS-CoV 3CL protease
2zu2A	-64.3684	P0C6U2	Human coronavirus strain 229E	3C-like proteinase
2q6dB	-63.7405	P0C6Y3	infectious bronchitis virus IBV	Infectious bronchitis virus (IBV) main protease
4zroD	-63.1005	Q98VG9	Feline infectious peritonitis virus (strain 79-1146)	3C-like proteinase
5hyoA	-62.3811	U6BPB2	porcine epidemic diarrhoea virus	PEDV 3CLpro
5wklA	-62.0095	K9N638	Middle East respiratory syndrome coronavirus	Orf1a protein
6l70A	-61.3982	K4L9I6	porcine epidemic diarrhoea virus	PEDV main protease
3tloA	-61.0346	P0C6U6	Human coronavirus NL	3C-like proteinase
5gwzB	-60.9263	P0C6V6	Porcine epidemic diarrhea virus (strain CV777)	PEDV main protease
5c3nA	-60.0699	V9TU05	Middle East respiratory syndrome coronavirus	ORF1a protein
2ampB	-59.2108	P0C6Y5	transmissible gastroenteritis virus TGEV	3C-like proteinase
4yogA	-59.1457	P0C6W3	Bat coronavirus HKU4	3C-like proteinase
4zuhB	-58.2564	R4JK63	porcine epidemic diarrhoea virus	PEDV 3C-Like protease
4f49A	-57.7283	P0C6V2	Porcine transmissible gastroenteritis coronavirus (STRAIN PURDUE)	3C-like proteinase
4yluB	-53.7995	V9TU12	Middle East respiratory syndrome coronavirus	ORF1a protein
6ohwB	-51.1758	Q696P8	Human coronavirus strain OC43	Spike surface glycoprotein
3d23C	-50.1771	P0C6U3	Human coronavirus HKU1 (isolate N1)	3C-like proteinase
6jijB	-49.6361	P0C6X9	Murine hepatitis virus (strain A59)	Replicative polyprotein 1ab
2q6fB	-47.7368	P0C6V5	infectious bronchitis virus IBV	Infectious bronchitis virus (IBV) main protease
5ynpA	-42.3545	K0BWD0	human betacoronavirus 2c EMC	nsp16 protein

Table S19. List of Coronaviridae family targets, to which ticagrelor successfully docked.

PDB ID with Chain	***Score***	***UniProt***	***Organism***	***Protein name***
5c5oA	-75.5513	P0C6X7	Severe acute respiratory syndrome coronavirus	3C-like proteinase
4wmeA	-70.1036	W6A941	Middle East respiratory syndrome coronavirus	MERS-CoV 3CL protease

1p9uB	-68.6317	P0C6Y5	transmissible gastroenteritis virus TGEV	putative coronavirus nsp2 (3CL-PRO)
4yluB	-65.3582	V9TU12	Middle East respiratory syndrome coronavirus	ORF1a protein
5rhfA	-64.1523	P0DTD1	Wuhan coronavirus	3C-like proteinase
2ynbA	-63.7354	P0C6T4	Bat coronavirus HKU4	3C-LIKE PROTEINASE
5hyoA	-63.5243	U6BPB2	porcine epidemic diarrhoea virus	PEDV 3CLpro
2q6dA	-62.7349	P0C6Y3	infectious bronchitis virus IBV	Infectious bronchitis virus (IBV) main protease
3tloB	-62.3181	P0C6U6	Human coronavirus NL	3C-like proteinase
5wklA	-62.2231	K9N638	Middle East respiratory syndrome coronavirus	Orf1a protein
3mj5A	-62.0705	P0C6U8	Severe acute respiratory syndrome coronavirus	Replicase polyprotein 1a
4zuhB	-60.7488	R4JK63	porcine epidemic diarrhoea virus	PEDV 3C-Like protease
5c3nA	-59.9382	V9TU05	Middle East respiratory syndrome coronavirus	ORF1a protein
1p9sA	-57.9706	P0C6U2	Human coronavirus strain 229E	Replicase polyprotein 1ab
4yoiB	-56.3491	P0C6W3	Bat coronavirus HKU4	3C-like proteinase
6jijA	-56.2687	P0C6X9	Murine hepatitis virus (strain A59)	Replicative polyprotein 1ab
6ohwB	-55.3749	Q696P8	Human coronavirus strain OC43	Spike surface glycoprotein
4rezA	-55.137	M4STU1	Human betacoronavirus 2c strain Jordan-N3/2012	ORF1ab protein
5gwzB	-54.5812	P0C6V6	Porcine epidemic diarrhea virus (strain CV777)	PEDV main protease
6l70B	-54.5203	K4L9I6	porcine epidemic diarrhoea virus	PEDV main protease
6fv1A	-53.7256	P0C6X5	Human coronavirus NL	3C-like proteinase
5ynpA	-52.7649	K0BWD0	human betacoronavirus 2c EMC	nsp16 protein
6wkpC	-52.1371	P0DTC9	Wuhan coronavirus	Nucleoprotein
4f49A	-51.1088	P0C6V2	Porcine transmissible gastroenteritis coronavirus (STRAIN PURDUE)	3C-like proteinase
6nb4A	-49.9945	A0A140AYW5	Middle East respiratory syndrome coronavirus	Spike glycoprotein
4zroC	-49.4736	Q98VG9	Feline infectious peritonitis virus (strain 79-1146)	3C-like proteinase
5nfyA	-49.1399	Q1T6X8	SARS coronavirus Frankfurt 1	Polyprotein 1ab
3d23B	-48.0264	P0C6U3	Human coronavirus HKU1 (isolate N1)	3C-like proteinase
5yvdA	-48.0051	A0A0U2GPI9	Middle East respiratory syndrome coronavirus	Nsp15
6u7kB	-47.6625	Q91AV1	Porcine epidemic diarrhea virus (strain CV777)	Spike glycoprotein
5jilA	-47.2	Q3HS77	Rat coronavirus	Hemagglutinin-esterase
4pt5A	-46.8418	K4LC41	human betacoronavirus 2c EMC	Papain-like protease
2q6fB	-45.8974	P0C6V5	infectious bronchitis virus IBV	Infectious bronchitis virus (IBV) main protease
3cl5A	-45.0174	P15776	neonatal calf diarrhea virus	Hemagglutinin-esterase
4wurA	-44.9135	K9N7C7	Betacoronavirus England 1	Papain-like protease
5w9jL	-44.8716	K9N5Q8	Middle East respiratory syndrome coronavirus	Spike glycoprotein
6kl6A	-44.7723	K9N4V7	Middle East respiratory syndrome coronavirus	Nucleoprotein
4c7lA	-43.8266	P31614	murine hepatitis virus MHV-S	HEMAGGLUTININ-ESTERASE
6l5tA	-43.3615	A0A385H8D7	Swine acute diarrhea syndrome coronavirus	Peptidase C16

6q05C	-43.2054	K0BRG7	human betacoronavirus 2c EMC	Spike glycoprotein
4lmtA	-42.6323	P33469	Human coronavirus strain OC43	Nucleoprotein
6nozA	-42.5477	W8QLX4	porcine epidemic diarrhoea virus	Polyprotein
5i08C	-41.9181	Q0ZME7	Human coronavirus HKU1 (isolate N5)	Spike glycoprotein,Foldon chimera
5jifB	-41.512	O92367	Murine coronavirus (strain DVIM)	Hemagglutinin-esterase
6zozA	-40.7662	P0DTC2	Wuhan coronavirus	Spike glycoprotein
2gecB	-40.395	P32923	infectious bronchitis virus IBV	Nucleocapsid protein
6ackC	-40.2592	P59594	Severe acute respiratory syndrome coronavirus	Spike glycoprotein
2c86A	-39.7412	P69598	infectious bronchitis virus IBV	NUCLEOCAPSID PROTEIN
6y3yA	-39.7231	Q5MQD1	Human CoV/HKU1	Hemagglutinin-esterase
3jclC	-38.1993	P11224	Murine hepatitis virus (strain A59)	Spike glycoprotein
2ofzA	-37.6994	P59595	Severe acute respiratory syndrome-related coronavirus Tor2	Nucleocapsid protein
5n11A	-37.0068	Q4VID6	Human coronavirus strain OC43	Hemagglutinin-esterase
4l3nA	-36.4321	M4SVE7	Human betacoronavirus 2c strain Jordan-N3/2012	S protein
6m15A	-35.0929	A8JNZ2	Bat coronavirus HKU2	Spike glycoprotein
4h14A	-33.652	Q1HLC5	neonatal calf diarrhea virus	Spike glycoprotein
6u7hB	-33.4137	P15423	Human coronavirus strain 229E	spike glycoprotein
6zgfC	-33.1816	A0A6B9WHD3	Bat coronavirus isolate RaTG13	Spike glycoprotein
6qfyA	-31.9212	A0A1Z2WUW0	Porcine hemagglutinating encephalomyelitis coronavirus	Spike glycoprotein
6m16B	-30.4205	A0A2P1G1L3	Swine acute diarrhea syndrome coronavirus	Spike glycoprotein
6b7nB	-18.9639	A0A075E3D7	Deltacoronavirus SDCV/USA/Ohio137/2014	Spike protein
6jx7C	-14.9956	C6GHB7	FIPV	Feline Infectious Peritonitis Virus Spike Protein
6bfuC	-13.1488	A0A140ESF1	Porcine deltacoronavirus	Spike protein

Table S20. List of combined viral protein targets for all drugs, grouped by protein class.

no	***PDB ID***	***Class***	***no***	***PDB ID***	***Class***	***no***	***PDB ID***	***Class***
0	1h8tA	capsid protein	48	1a9mB	protease	96	3s54B	protease
1	1upnA	capsid protein	49	1bv7B	protease	97	3s85A	protease
2	3j2jA	capsid protein	50	1hviA	protease	98	3sacB	protease
3	3kicH	capsid protein	51	1hxwB	protease	99	3t3cA	protease
4	3kieC	capsid protein	52	1iiqA	protease	100	3ttpB	protease
5	3kieQ	capsid protein	53	1iiqB	protease	101	3vf7A	protease
6	3shmB	capsid protein	54	1ivqB	protease	102	4b75A	protease
7	3sm2A	capsid protein	55	1jldB	protease	103	4djqA	protease
8	5bnpA	capsid protein	56	1k1uB	protease	104	4djqB	protease
9	5up4J	capsid protein	57	1mrwA	protease	105	4dqfB	protease
10	6aj2A	capsid protein	58	1mrwB	protease	106	4ejlB	protease
11	6b2hA	capsid protein	59	1mrxA	protease	107	4fivA	protease
12	6bhtD	capsid protein	60	1w5vA	protease	108	4mc2A	protease
13	6crrA	capsid protein	61	1w5vB	protease	109	4mc6A	protease
14	6cv3A	capsid protein	62	1wbkA	protease	110	4njtB	protease
15	6iioA	capsid protein	63	1wofA	protease	111	4njuA	protease
16	6lhbA	capsid protein	64	2a4fB	protease	112	4q1wA	protease
17	6sk5A	capsid protein	65	2avmA	protease	113	4q1yB	protease
18	6sk6A	capsid protein	66	2bbbB	protease	114	4upjB	protease
19	6snbA	capsid protein	67	2cenA	protease	115	5ah7B	protease
20	6snwA	capsid protein	68	2hpeA	protease	116	5ah8A	protease
21	6ubmA	capsid protein	69	2hpeB	protease	117	5cokB	protease
22	6wdtA	capsid protein	70	2i4uA	protease	118	5ivtB	protease
23	7bznA	capsid protein	71	2i4vB	protease	119	5lc0A	protease
24	1mqnE	glycoprotein	72	2i4xB	protease	120	5ufzA	protease
25	3ngbG	glycoprotein	73	2iphB	protease	121	5upjA	protease
26	4adjA	glycoprotein	74	2m9qA	protease	122	6b3gA	protease
27	4adjB	glycoprotein	75	2o4pB	protease	123	6b3hB	protease
28	4nm8D	glycoprotein	76	2pk5A	protease	124	6bz2B	protease
29	5tdgC	glycoprotein	77	2q55A	protease	125	6dj2B	protease
30	5tgoF	glycoprotein	78	2q5kA	protease	126	6dv0B	protease
31	5tgvB	glycoprotein	79	2r5pB	protease	127	6fivA	protease
32	5u68C	glycoprotein	80	2r5qA	protease	128	6ixdA	protease
33	5udcF	glycoprotein	81	2r5qB	protease	129	6kk6A	protease
34	5v8mA	glycoprotein	82	2uy0A	protease	130	6o57A	protease
35	5v8mF	glycoprotein	83	2yolA	protease	131	6o57B	protease
36	5xl8A	glycoprotein	84	3dk1B	protease	132	6o5aA	protease
37	3ovnA	integrase	85	3ebzB	protease	133	6o5aB	protease
38	6rwmB	integrase	86	3ecgA	protease	134	6oglA	protease

39	6rwmD	integrase	87	3ekqB	protease	135	6oxpB	protease
40	1p72B	kinase	88	3ekwA	protease	136	6p9aA	protease
41	3hw4A	polymerase	89	3gi6A	protease	137	6pjmB	protease
42	3qgfA	polymerase	90	3i7eB	protease	138	6pu8B	protease
43	5d98A	polymerase	91	3lkwA	protease	139	6w9cB	protease
44	6kv5A	polymerase	92	3lzsB	protease	140	5vqqA	ribonuclease
45	6qcsA	polymerase	93	3nlsB	protease	141	6c0pA	ribonuclease
46	6qcwA	polymerase	94	3qrmA	protease	142	4fzbC	synthase
47	1a8kA	protease	95	3s45A	protease	143	4kb9A	synthase

Table S21. List of combined coronaviridae family protein targets for all drugs, grouped by protein class.

no	***PDB ID***	***Class***	***no***	***PDB ID***	***Class***	***no***	***PDB ID***	***Class***
0	6crxA	glycoprotein	32	3titA	protease	64	5rf4A	protease
1	6l8qB	glycoprotein	33	3tloB	protease	65	5rghA	protease
2	6nzkB	glycoprotein	34	3vb3B	protease	66	5rgqA	protease
3	6zp2C	glycoprotein	35	3vb5A	protease	67	5rhfA	protease
4	6wkpC	Nucleoprotein	36	4ovzA	protease	68	5wkjA	protease
5	2a5kA	peptidase	37	4ow0A	protease	69	5wkkA	protease
6	7bw4A	polymerase	38	4pt5A	protease	70	5wklA	protease
7	1p9uB	protease	39	4wmeA	protease	71	6fv1A	protease
8	1uk3A	protease	40	4wmeB	protease	72	6jijA	protease
9	1wofA	protease	41	4wurA	protease	73	6l70B	protease
10	1wofB	protease	42	4xfqA	protease	74	6lnyA	protease
11	2ampA	protease	43	4yluA	protease	75	6lo0A	protease
12	2bx4A	protease	44	4yluB	protease	76	6nozA	protease
13	2h2zA	protease	45	4yo9A	protease	77	6w9cB	protease
14	2q6dA	protease	46	4yo9B	protease	78	6w9cC	protease
15	2q6dB	protease	47	4yoiA	protease	79	6wnpA	protease
16	2q6fA	protease	48	4yoiB	protease	80	6xa4A	protease
17	2vj1B	protease	49	4zroA	protease	81	6xbhA	protease
18	2ynaA	protease	50	4zroC	protease	82	6xbiB	protease
19	2ynaB	protease	51	4zroD	protease	83	6xhlA	protease
20	2ynbA	protease	52	5b6oA	protease	84	6xhlB	protease
21	2ynbB	protease	53	5c3nA	protease	85	6xhnB	protease
22	2zu2A	protease	54	5c5oA	protease	86	6xhoB	protease
23	2zu2B	protease	55	5gwyA	protease	87	6xqsA	protease
24	3atwB	protease	56	5gwzA	protease	88	6xqtA	protease
25	3aw0A	protease	57	5gwzB	protease	89	5ynbA	transferase
26	3d23B	protease	58	5hyoA	protease	90	5ynfA	transferase
27	3d23C	protease	59	5n19A	protease	91	5ynnA	transferase
28	3d23D	protease	60	5n5oA	protease	92	5ynoA	transferase
29	3e91A	protease	61	5nh0A	protease	93	5ynpA	transferase
30	3m3sB	protease	62	5nh0B	protease	94	5ynqA	transferase
31	3mj5A	protease	63	5r84A	protease	95	7c2jA	transferase

Calculated binding modes and 2D-binding projections of novel drugs in the repurposing experiment (12-22) with no prior reported 3CI^{pro} activity data.

Olodaterol

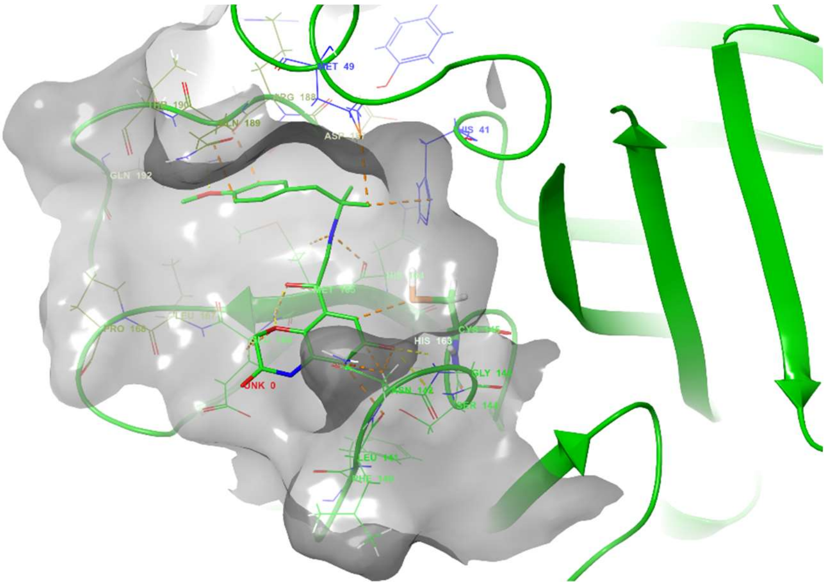

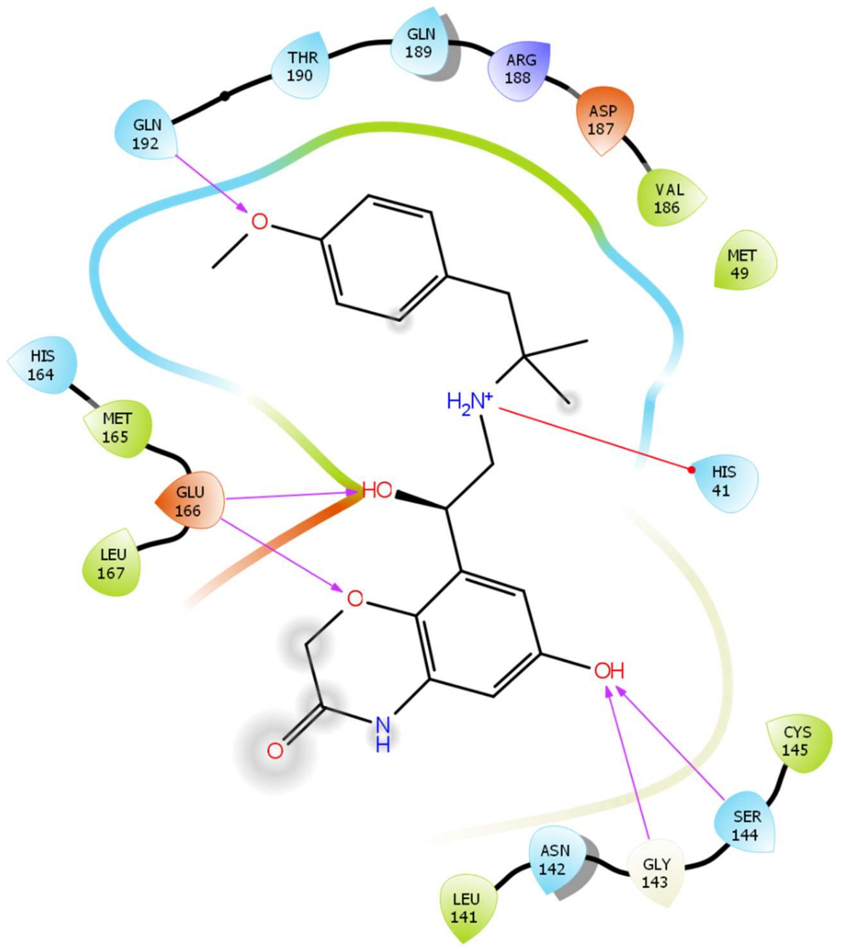

Xanthinol

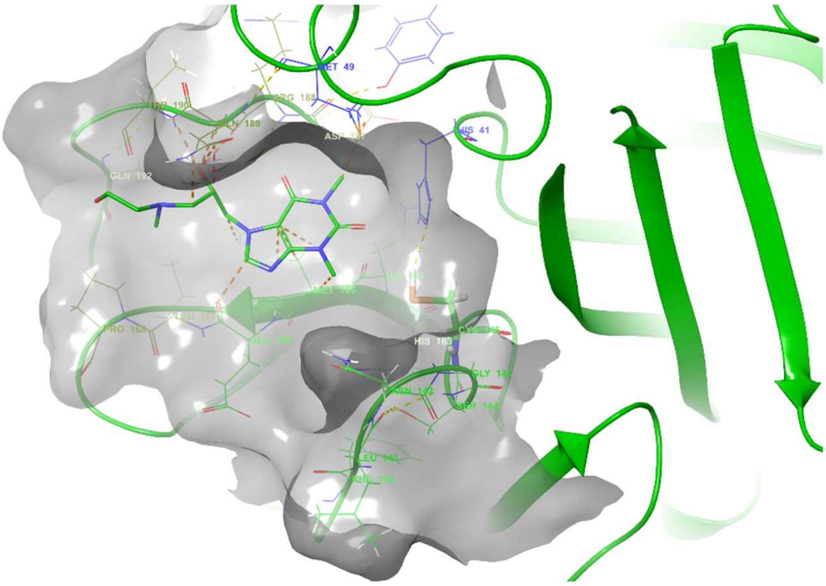

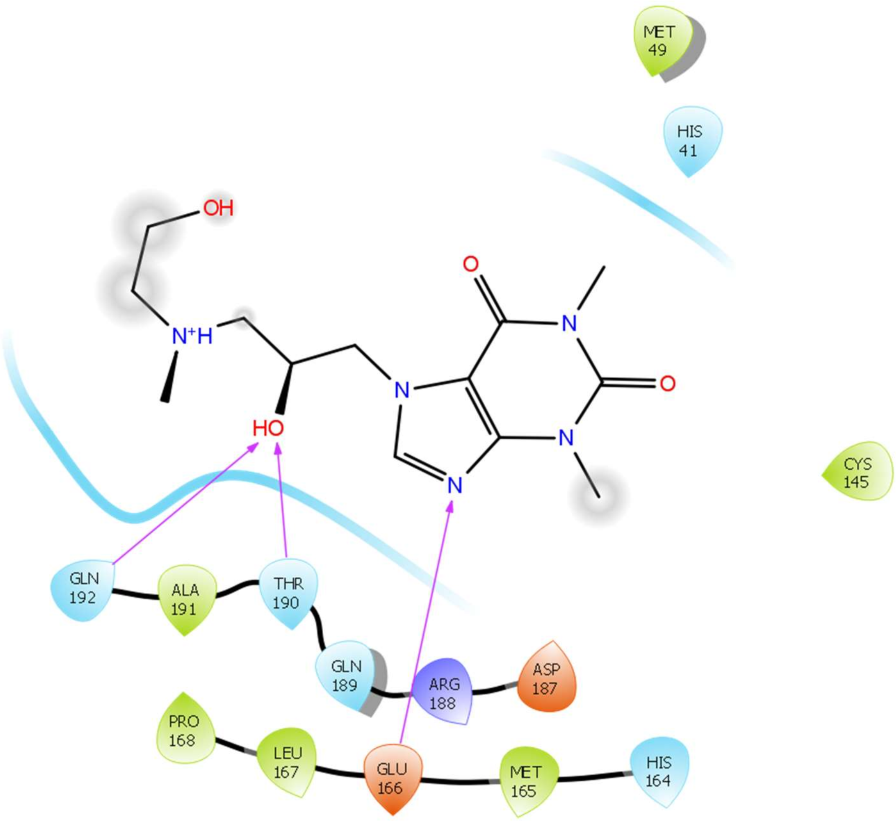

Penbutolol

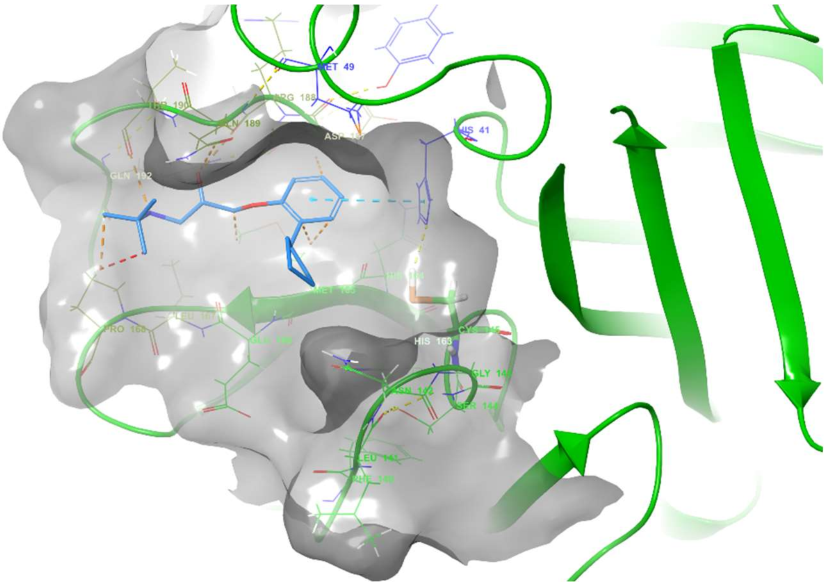

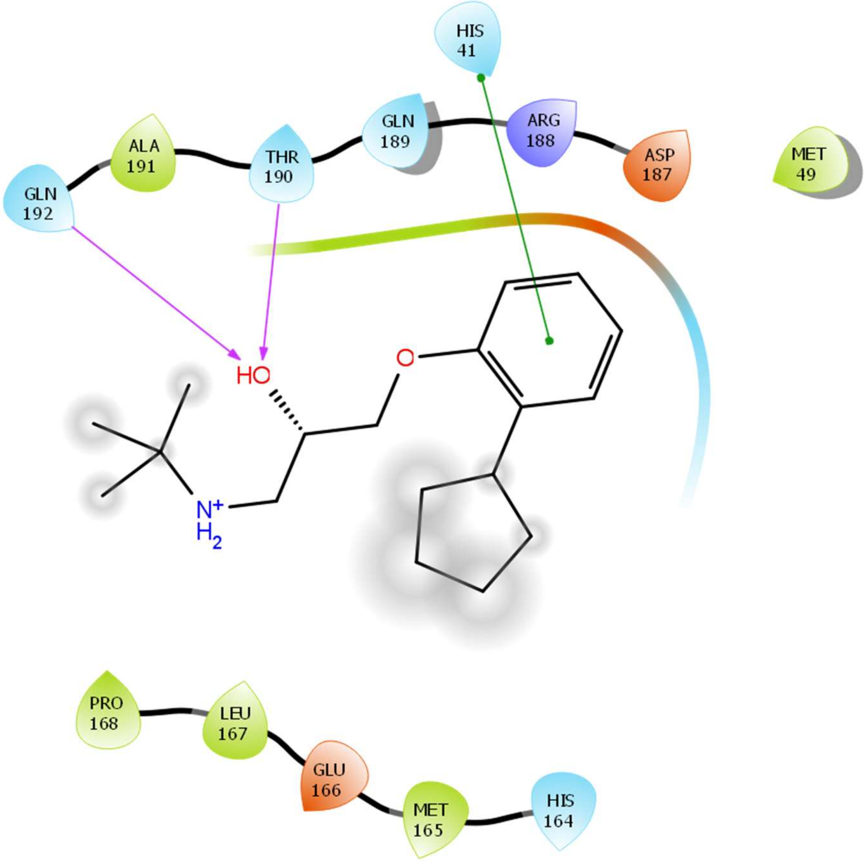
HIS 41
GLN 192
ALA 191
THR 190
GLN 189
ARG 188
ASP 187
MET 49
HO
O
N+ H2
PRO 168
LEU 167
GLU 166
MET 165
HIS 164

Alloin

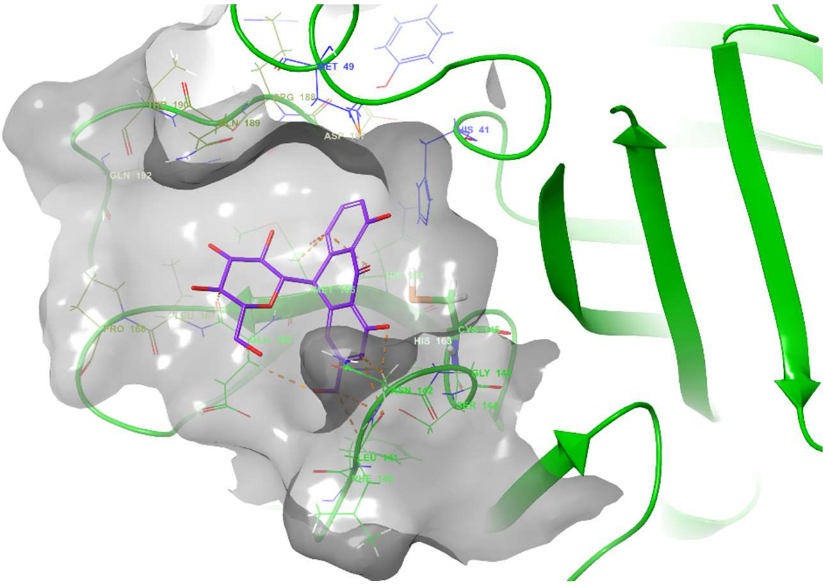

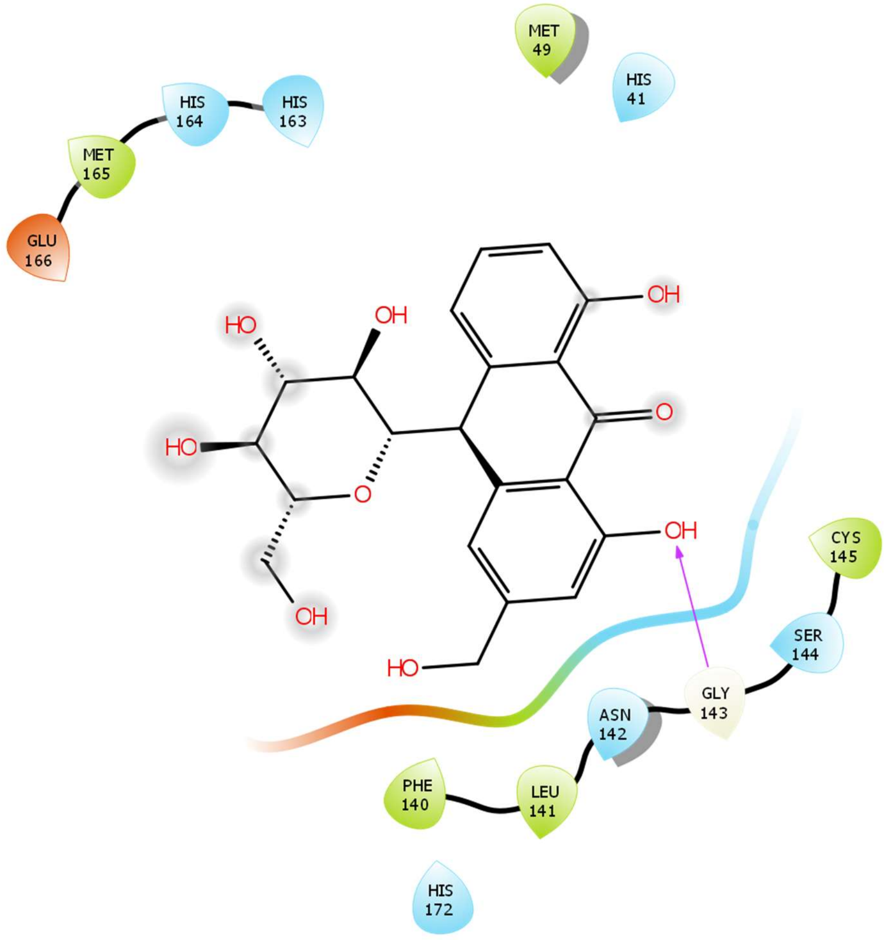

Piritramide

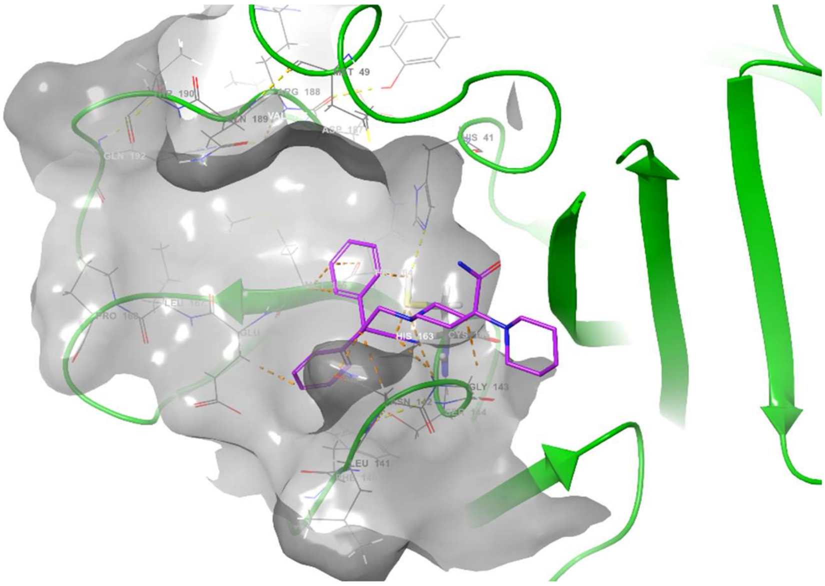

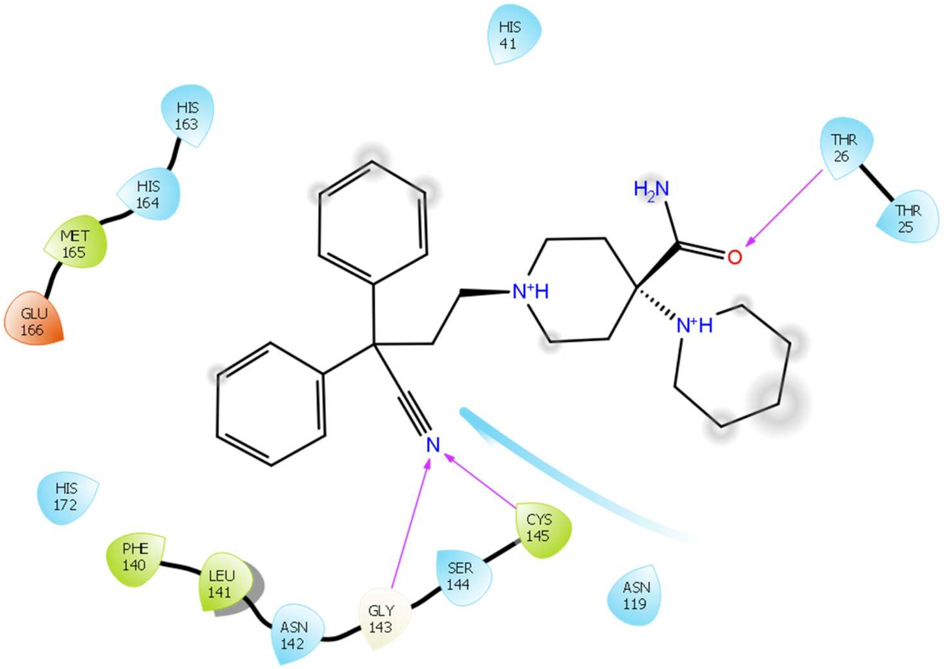
HIS 41
HIS 163
HIS 164
MET 165
GLU 166
THR 26
THR 25
H2N
O
N+H
N+H
N
HIS 172
PHE 140
LEU 141
ASN 142
GLY 143
SER 144
CYS 145
ASN 119

Sultopride

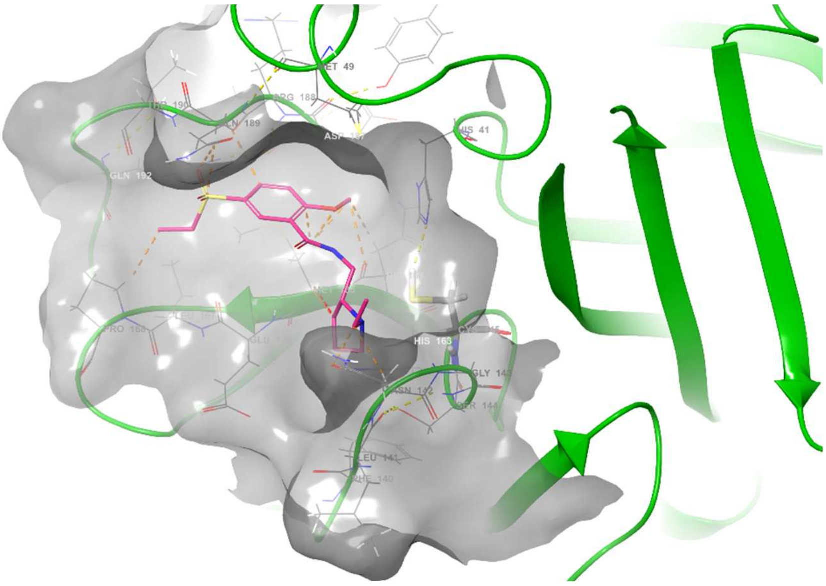

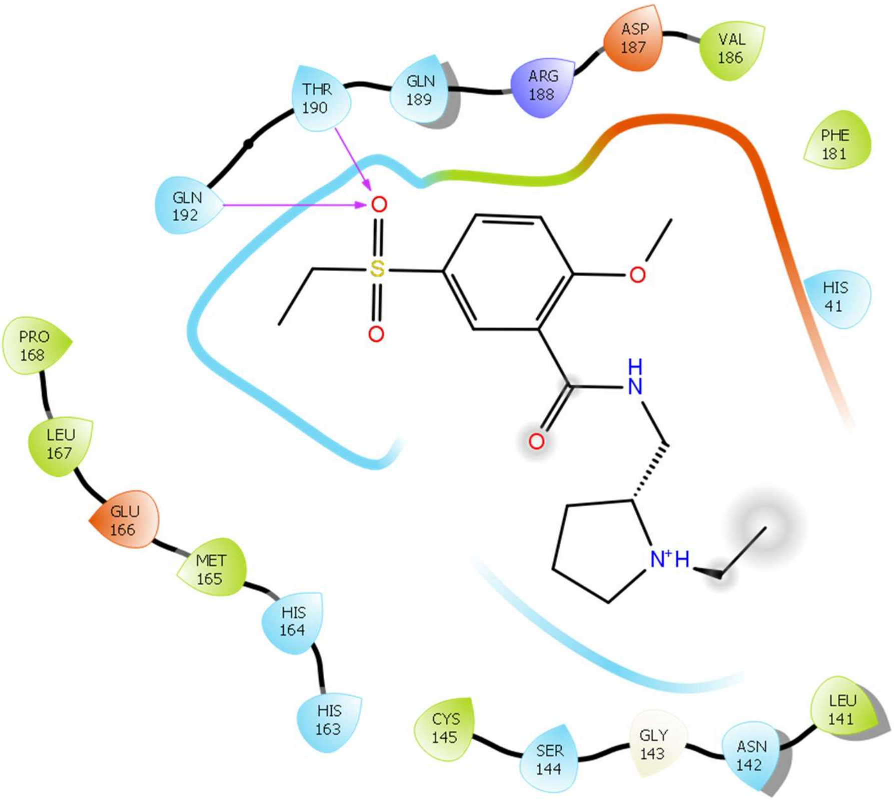

THR 190
GLN 189
ARG 188
ASP 187
VAL 186
PHE 181
GLN 192
HIS 41
PRO 168
LEU 167
GLU 166
MET 165
HIS 164
HIS 163
CYS 145
SER 144
GLY 143
ASN 142
LEU 141

Udenafil

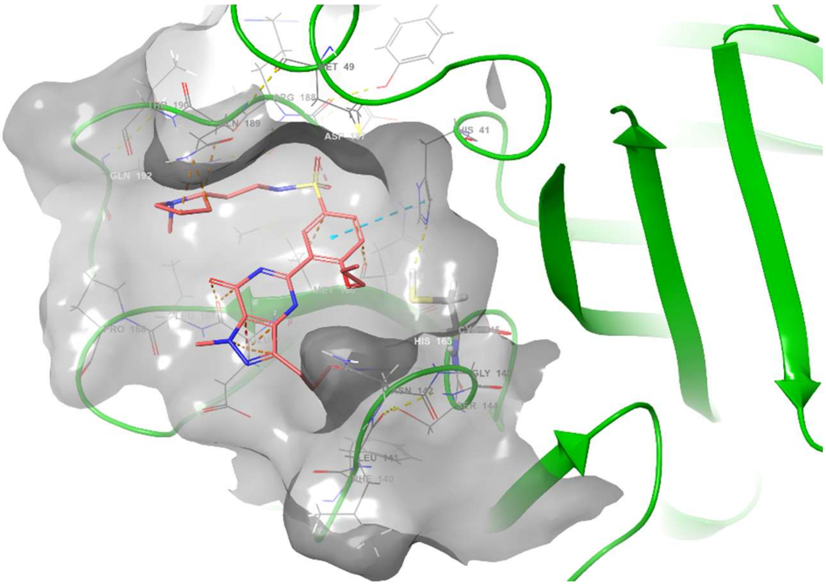
GLN 192
HIS 41
HIS 163

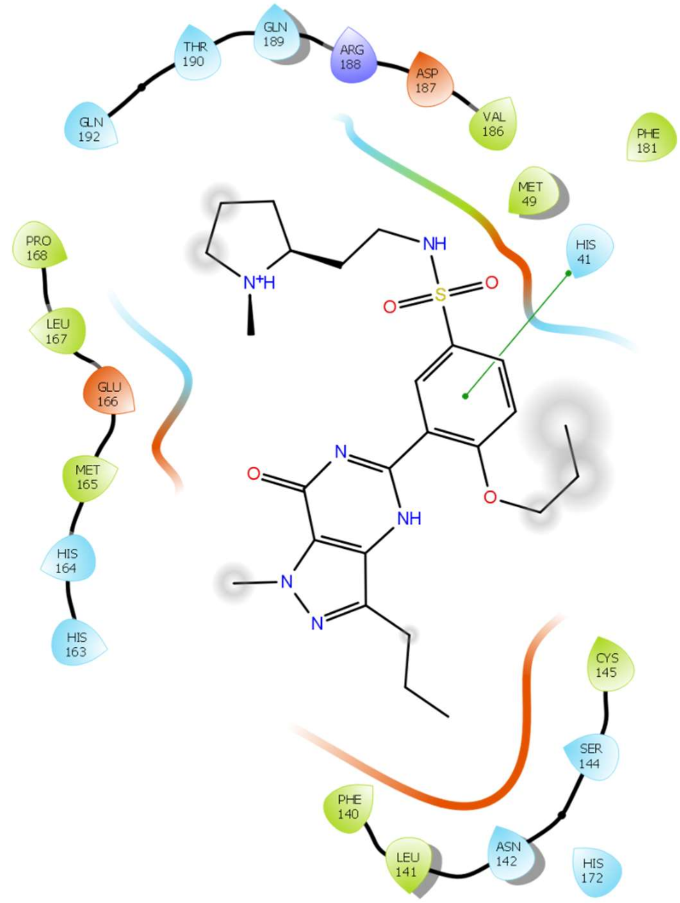
THR 190
GLN 189
ARG 188
ASP 187
VAL 186
PHE 181
GLN 192
MET 49
HIS 41
PRO 168
LEU 167
GLU 166
MET 165
HIS 164
HIS 163
CYS 145
SER 144
PHE 140
LEU 141
ASN 142
HIS 172

Doravirine

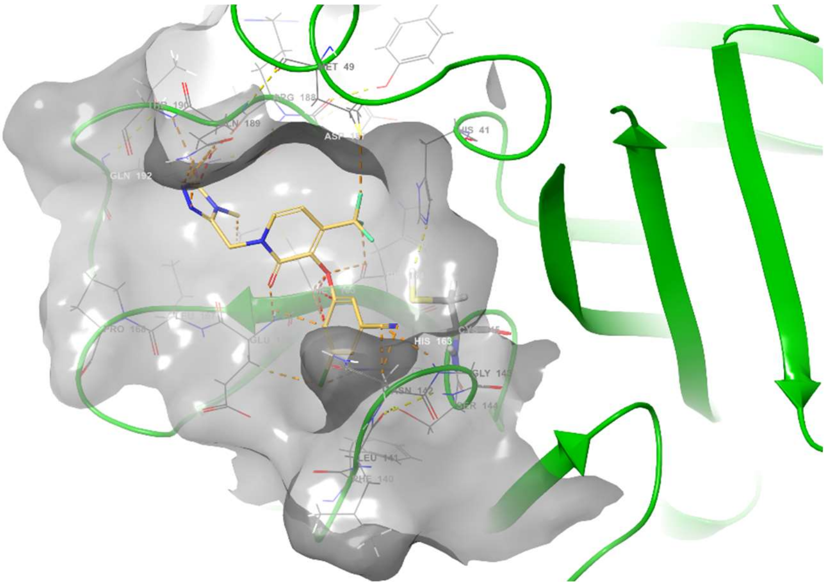

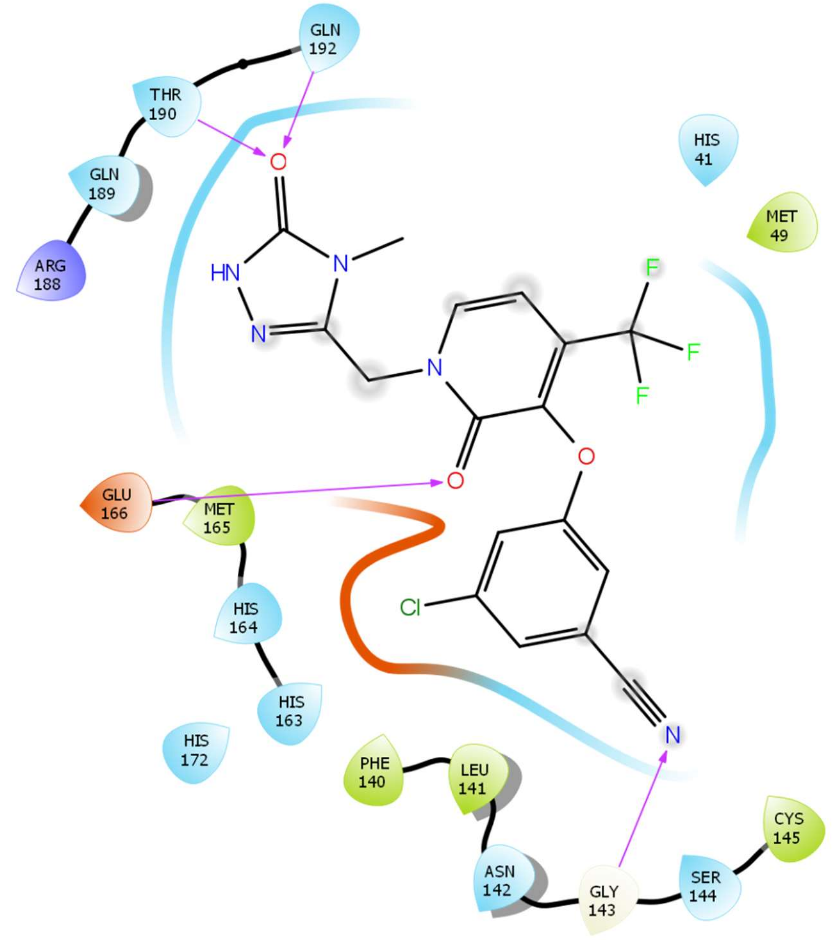

Valbenazine

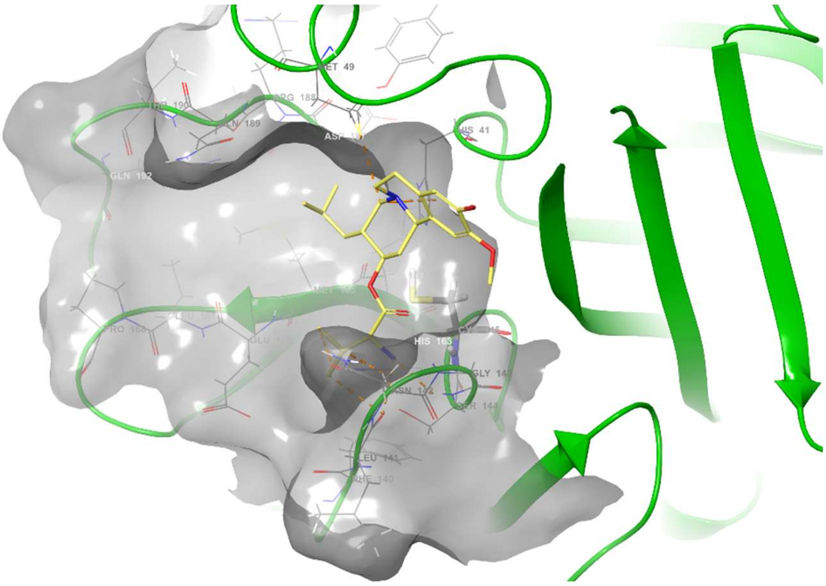
GLN 192
HIS 41
HIS 163
LEU 141

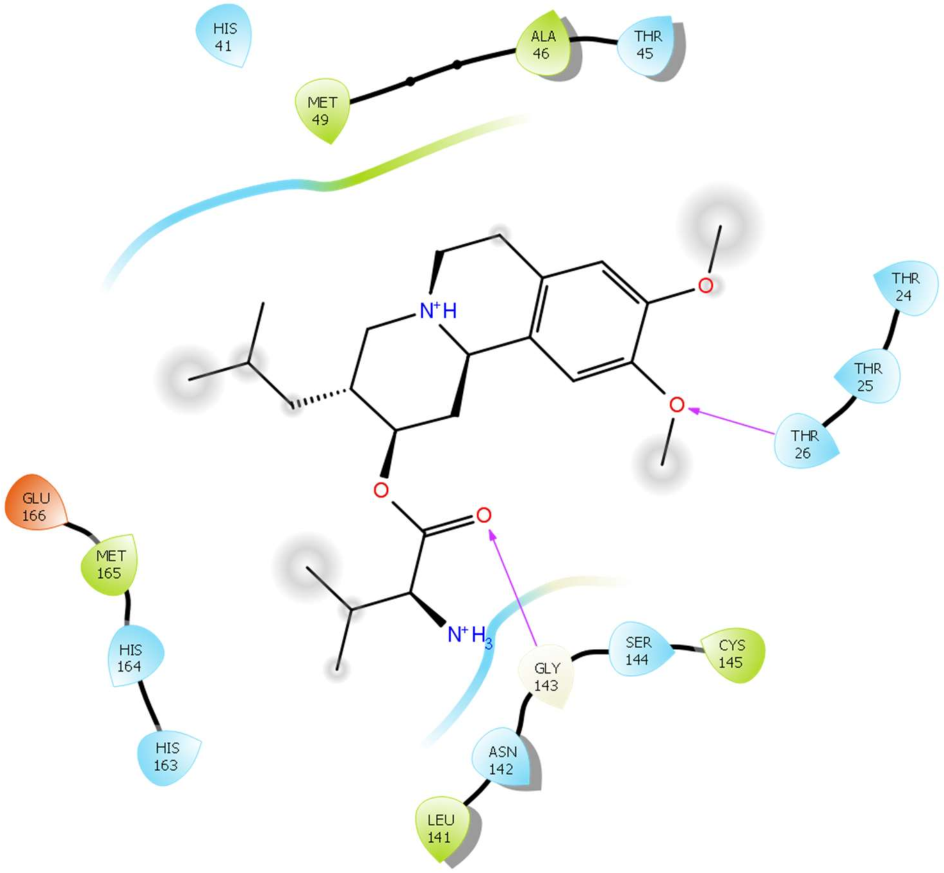
HIS 41
MET 49
ALA 46
THR 45
N⁺H
THR 24
THR 25
THR 26
GLU 166
MET 165
HIS 164
HIS 163
N⁺H3
GLY 143
SER 144
CYS 145
ASN 142
LEU 141

Arzoxifene

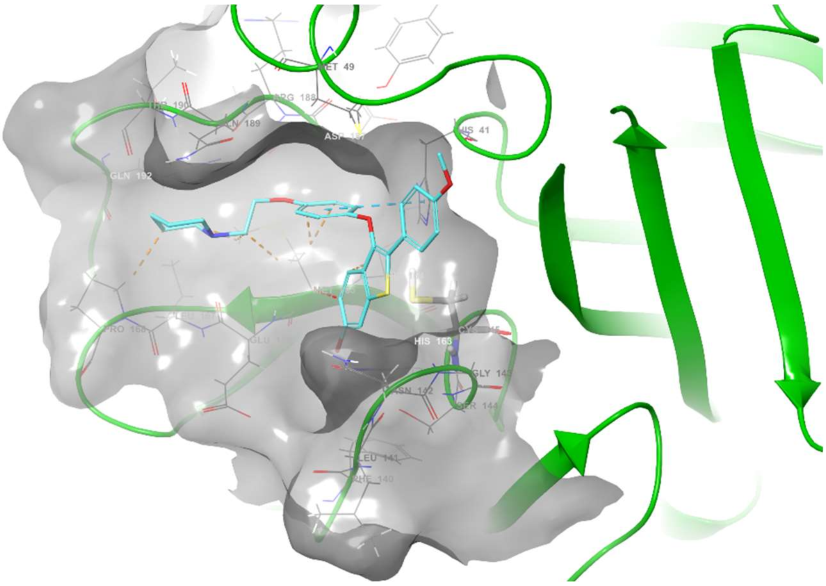

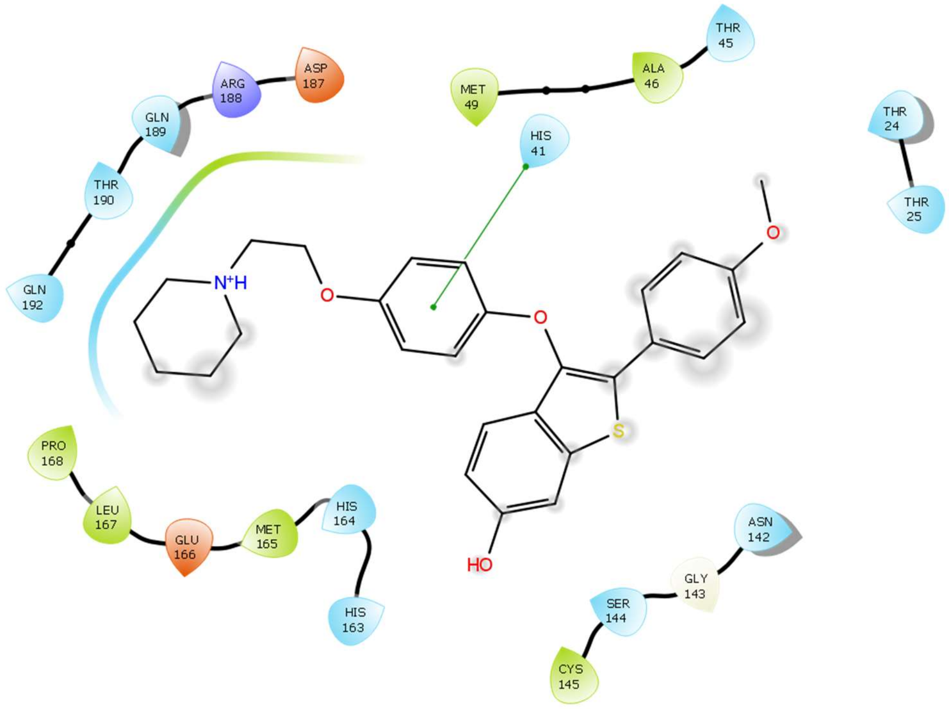
ASP 187
ARG 188
GLN 189
THR 190
GLN 192
MET 49
ALA 46
THR 45
HIS 41
THR 24
THR 25
N+H
O
O
O
S
HO
PRO 168
LEU 167
GLU 166
MET 165
HIS 164
HIS 163
ASN 142
GLY 143
SER 144
CYS 145

Risdiplam

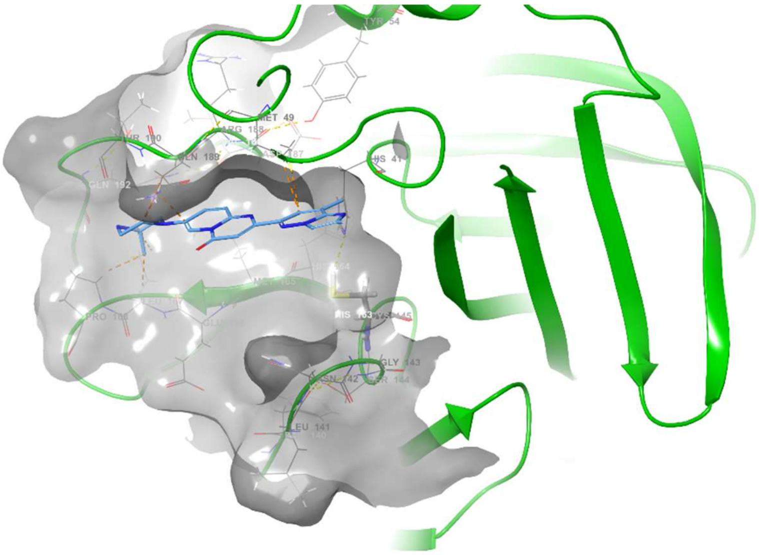

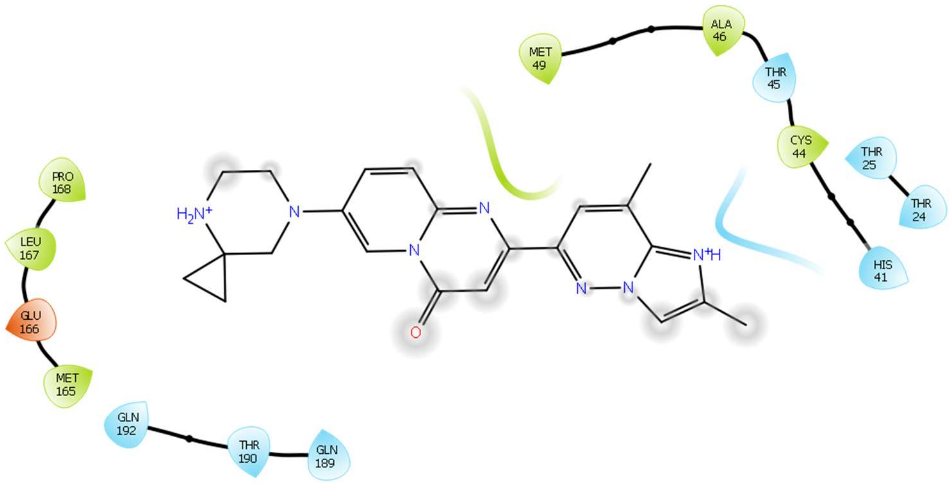
MET 49
ALA 46
THR 45
CYS 44
THR 25
THR 24
HIS 41
PRO 168
LEU 167
GLU 166
MET 165
GLN 192
THR 190
GLN 189
H2N+
N
N
N
O
N
N
N+H

Inverse Docking Fingerprint Target Case Study on Papain-Like Protease (PL^{pro}, PDB ID: 4ow0B)

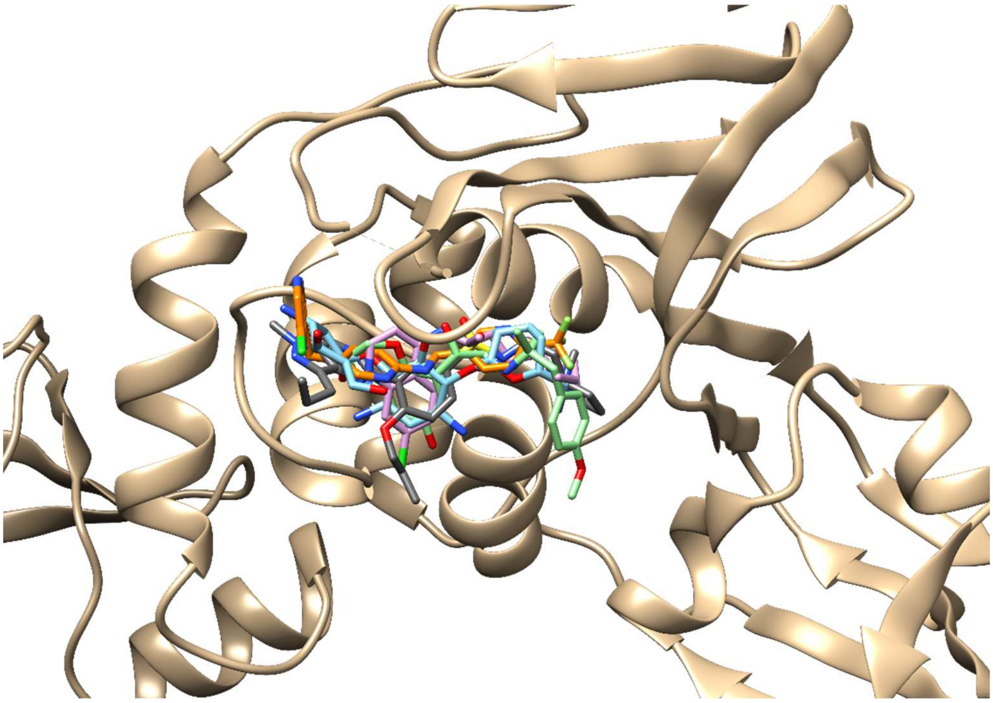

Figure S1. PL^{pro} binding pocket with docked representatives from groups with similar fingerprint profiles: dibekacin (blue), hydroxychloroquine (purple), olodaterol (green), pexidartinib (orange), and udenafil (gray).

Dibekacin

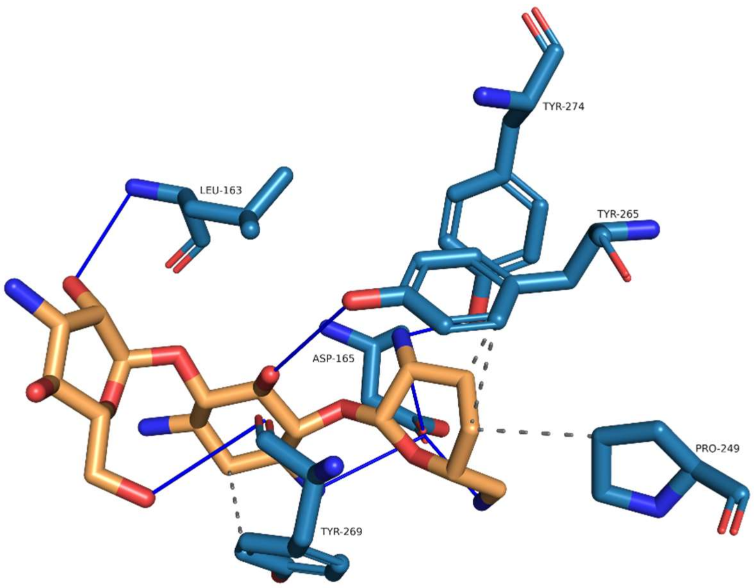

Residue	*Interaction*
PRO-249	Hydrophobic Interactions (gray dotted line)
TYR-265	Hydrophobic Interactions (gray dotted line)
TYR-269	Hydrophobic Interactions (gray dotted line)
LEU-163	Hydrogen Bonds (blue full line)
ASP-165	Hydrogen Bonds (blue full line)
TYR-265	Hydrogen Bonds (blue full line)
TYR-269	Hydrogen Bonds (blue full line)
TYR-274	Hydrogen Bonds (blue full line)

Hydroxychloroquine

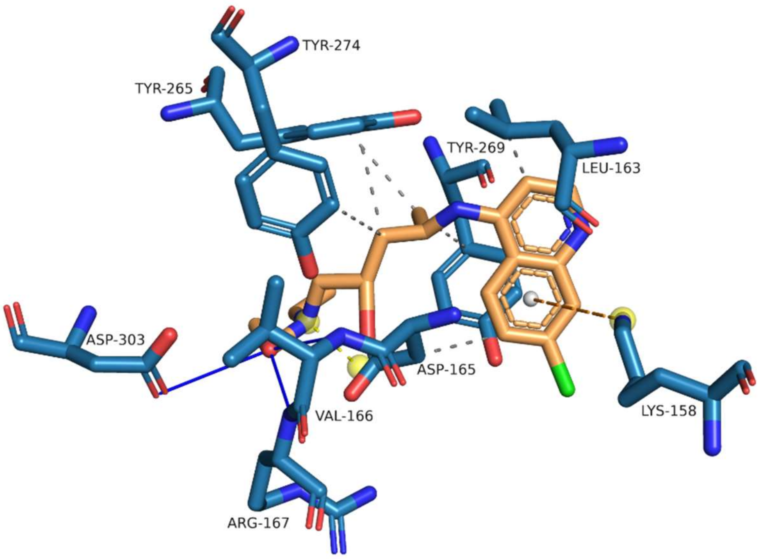

Residue	*Interaction*
LEU-163	Hydrophobic Interactions (gray dotted line)
ASP-165	Hydrophobic Interactions (gray dotted line)
TYR-265	Hydrophobic Interactions (gray dotted line)
TYR-269	Hydrophobic Interactions (gray dotted line)
TYR-274	Hydrophobic Interactions (gray dotted line)
VAL-166	Hydrogen Bonds (blue full line)
ARG-167	Hydrogen Bonds (blue full line)
ASP-303	Hydrogen Bonds (blue full line)
LYS-158	π-Cation Interactions (orange dotted line)
ASP-165	Salt Bridges (yellow dotted line)

Olodaterol

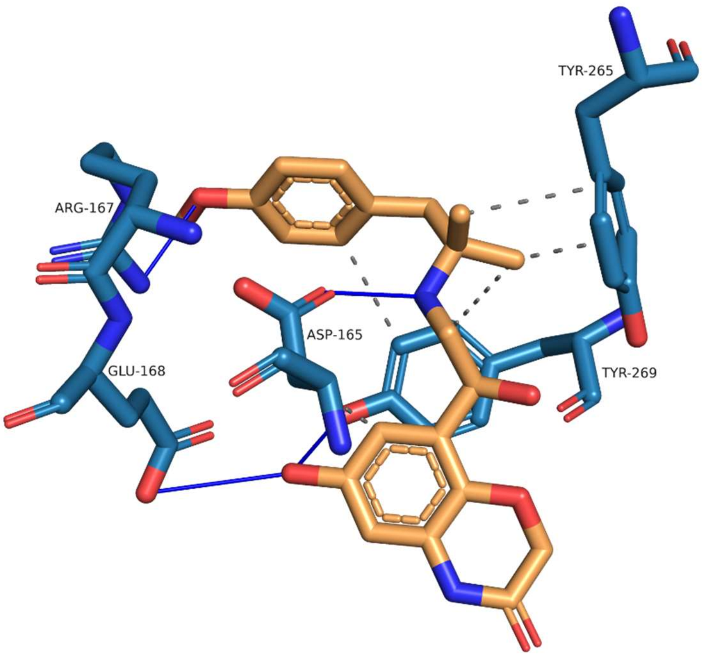

Residue	***Interaction***
ASP-165	Hydrophobic Interactions (gray dotted line)
TYR-265	Hydrophobic Interactions (gray dotted line)
TYR-269	Hydrophobic Interactions (gray dotted line)
ASP-165	Hydrogen Bonds (blue full line)
ARG-167	Hydrogen Bonds (blue full line)
GLU-168	Hydrogen Bonds (blue full line)
TYR-269	Hydrogen Bonds (blue full line)

Pexidartinib

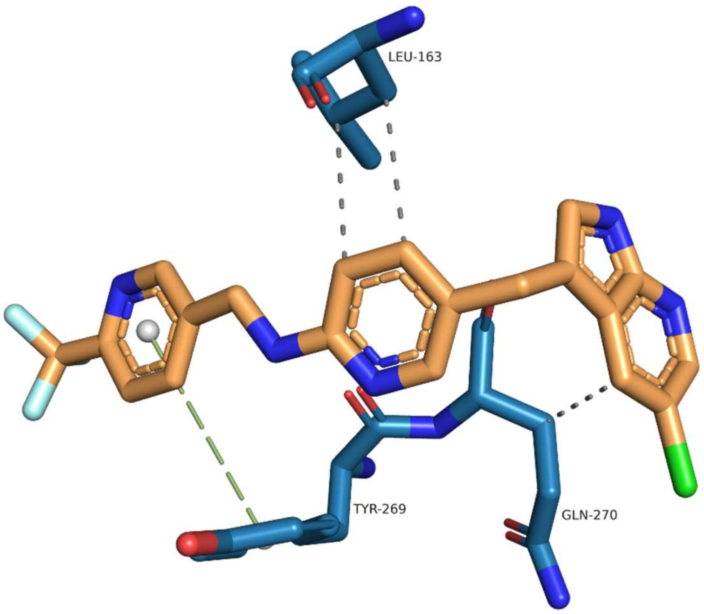

Residue	*Interaction*
LEU-163	Hydrophobic Interactions (gray dotted line)
GLN-270	Hydrophobic Interactions (gray dotted line)
TYR-269	π-Stacking (green dotted line)

Udenafil

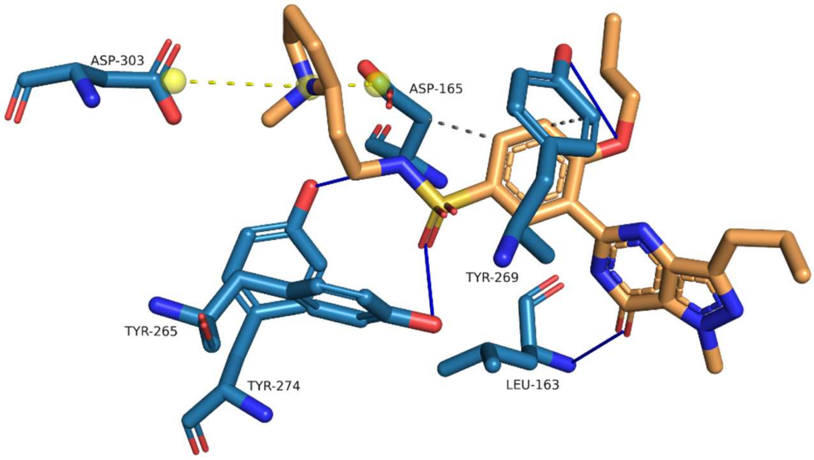

Residue	***Interaction***
ASP-165	Hydrophobic Interactions (gray dotted line)
TYR-269	Hydrophobic Interactions (gray dotted line)
LEU-163	Hydrogen Bonds (blue full line)
TYR-265	Hydrogen Bonds (blue full line)
TYR-269	Hydrogen Bonds (blue full line)
TYR-274	Hydrogen Bonds (blue full line)
ASP-165	Salt Bridges (yellow dotted line)
ASP-303	Salt Bridges (yellow dotted line)

Fingerprint Compound Case Study on Olodaterol With One Representative Target Protein From Each Class.

3C-like protease (PDB ID: 2a5kA)

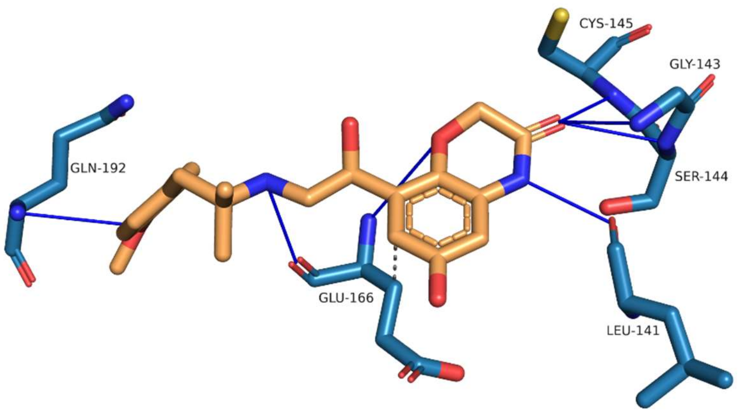

Residue	*Interaction*
GLU-166	Hydrophobic Interactions (gray dotted line)
LEU-141	Hydrogen Bonds (blue full line)
GLY-143	Hydrogen Bonds (blue full line)
SER-144	Hydrogen Bonds (blue full line)
CYS-145	Hydrogen Bonds (blue full line)
GLU-166	Hydrogen Bonds (blue full line)
GLN-192	Hydrogen Bonds (blue full line)

Papain-like protease (PDB ID: 4ow0A)

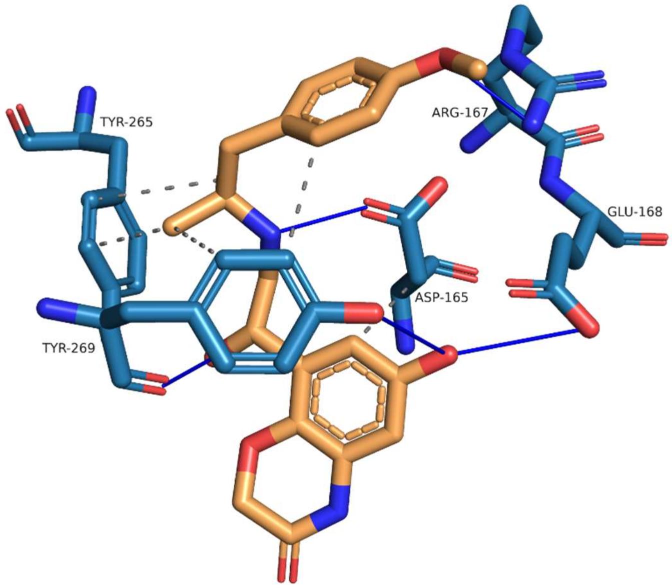

Residue	***Interaction***
ASP-165	Hydrophobic Interactions (gray dotted line)
TYR-265	Hydrophobic Interactions (gray dotted line)
TYR-269	Hydrophobic Interactions (gray dotted line)
ASP-165	Hydrogen Bonds (blue full line)
ARG-167	Hydrogen Bonds (blue full line)
GLU-168	Hydrogen Bonds (blue full line)
TYR-269	Hydrogen Bonds (blue full line)

NSP16 protein – transferase (PDB ID: 5ynpA)

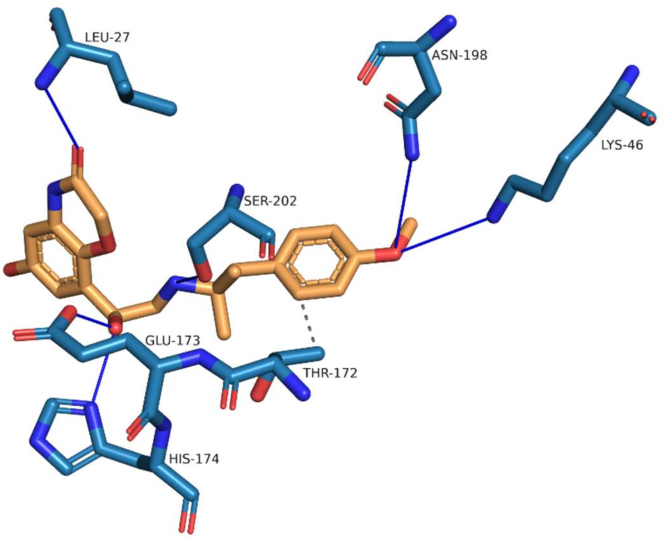

Residue	*Interaction*
THR-172	Hydrophobic Interactions (gray dotted line)
LEU-27	Hydrogen Bonds (blue full line)
LYS-46	Hydrogen Bonds (blue full line)
GLU-173	Hydrogen Bonds (blue full line)
HIS-174	Hydrogen Bonds (blue full line)
ASN-198	Hydrogen Bonds (blue full line)
SER-202	Hydrogen Bonds (blue full line)

Spike glycoprotein (PDB ID: 6l8qB)

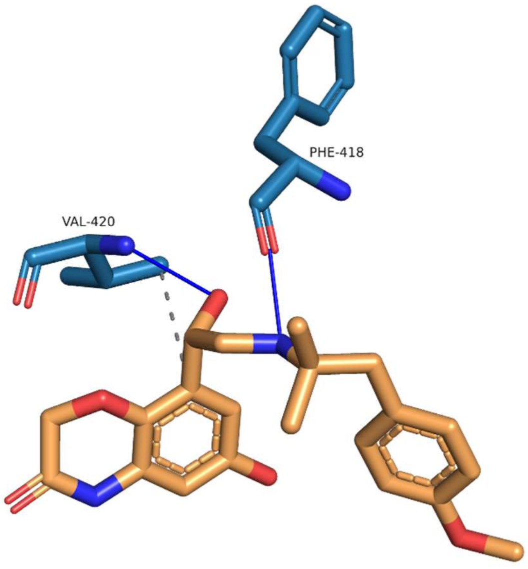

Residue	*Interaction*
VAL-420	Hydrophobic Interactions (gray dotted line)
PHE-418	Hydrogen Bonds (blue full line)
VAL-420	Hydrogen Bonds (blue full line)

Figures were prepared with PyMol, Chimera and PLIP tools.
